# Sex differences in fetal growth and immediate birth outcomes in a low-risk Caucasian population

**DOI:** 10.1186/s13293-019-0261-7

**Published:** 2019-09-09

**Authors:** Sander Galjaard, Lieveke Ameye, Christoph C. Lees, Anne Pexsters, Tom Bourne, Dirk Timmerman, Roland Devlieger

**Affiliations:** 1 KU Leuven Department of Development and Regeneration: Pregnancy, Fetus and Neonate, Gynaecology and Obstetrics, University Hospitals Leuven, Herestraat 49, 3000 Leuven, Belgium; 2 Department of Obstetrics and Gynaecology, Division of Obstetrics and Prenatal Medicine, Erasmus MC, University Medical Centre, Wytemaweg 80, 3015 CN Rotterdam, the Netherlands; 3 Department of Development and Regeneration, KU Leuven, Herestraat 49, 3000 Leuven, Belgium; 4 Centre for Fetal Care, Queen Charlotte’s and Chelsea Hospital, Imperial College Healthcare NHS Trust, Imperial College London, London, UK; 5 Department of Gynaecology and Obstetrics, Queen Charlotte’s and Chelsea Hospital, Imperial College Healthcare NHS Trust, Imperial College London, London, UK

**Keywords:** Fetal anthropometric gender differences, Prenatal growth, Birth outcomes, Perinatal and neonatal management

## Abstract

**Background:**

According to the WHO Multicentre Growth Reference Study Group recommendations, boys and girls have different growth trajectories after birth. Our aim was to develop gender-specific fetal growth curves in a low-risk population and to compare immediate birth outcomes.

**Methods:**

First, second, and third trimester fetal ultrasound examinations were conducted between 2002 and 2012. The data was selected using the following criteria: routine examinations in uncomplicated singleton pregnancies, Caucasian ethnicity, and confirmation of gestational age by a crown-rump length (CRL) measurement in the first trimester. Generalized Additive Model for Location, Scale and Shape (GAMLSS) was used to align the time frames of the longitudinal fetal measurements, corresponding with the methods of the postnatal growth curves of the WHO MGRS Group.

**Results:**

A total of 27,680 complete scans were selected from the astraia© ultrasound database representing 12,368 pregnancies. Gender-specific fetal growth curves for biparietal diameter (BPD), head circumference (HC), abdominal circumference (AC), and femur length (FL) were derived. The HC and BPD were significantly larger in boys compared to girls from 20 weeks of gestation onwards (*p* < 0.001) equating to a 3-day difference at 20–24 weeks. Boys were significantly heavier, longer, and had greater head circumference than girls (*p* < 0.001) at birth. The Apgar score at 1 min (*p* = 0.01) and arterial cord pH (*p* < 0.001) were lower in boys.

**Conclusions:**

These longitudinal fetal growth curves for the first time allow integration with neonatal and pediatric WHO gender-specific growth curves. Boys exceed head growth halfway of the pregnancy, and immediate birth outcomes are worse in boys than girls. Gender difference in intrauterine growth is sufficiently distinct to have a clinically important effect on fetal weight estimation but also on the second trimester dating. Therefore, these differences might already play a role in early fetal or immediate neonatal management.

**Electronic supplementary material:**

The online version of this article (10.1186/s13293-019-0261-7) contains supplementary material, which is available to authorized users.

## Background

Ultrasound has been an indispensable tool for diagnosis in obstetrics and fetal growth assessment for at least 4 decades [1–3]. Clinical management in pregnancies is increasing based on ultrasound measurements derived in the first trimester and on the recognition of pathological fetal growth, which depends on reliable, standardized growth curves [4]. Although it is widely known that boys are slightly larger than girls in the first trimester and at birth, there has been no consideration of fetal gender in the development and interpretation of fetal growth curves [5–8]. This gender dichotomy seems important since there is clear evidence that gestation-specific neonatal outcomes are worse in boys, indicating the vulnerability of the male embryo and fetus [9, 10].

Many charts have been published on fetal growth using different methodologies from the early 1990s until early in this decade, after which new (dating) protocols emerged [11]. Most normal ranges were designed from cross-sectional data [12–19], which by their nature may represent fetal size at a given point but do not directly infer growth. To derive information on fetal growth, statistical strategies using repeat measurements are required but longitudinal methodologies are utilized more rarely [20, 21]. Given these complexities, the World Health Organization (WHO) Multicentre Growth Reference Study (MGRS) Group recommended Generalized Additive Model for Location, Scale and Shape (GAMLSS) for the construction of the WHO Growth Standards [22, 23]. Most recently, growth charts have been developed in the regions of Europe and the USA and customization based on ethnicity is reported [11, 12, 18, 19, 24].

Our aim was to develop gender-specific longitudinal first, second, and third trimester normal growth reference curves within a low-risk Caucasian population with a robust WHO-endorsed longitudinal statistical methodology. Further, we aimed to test the validity of these curves by comparing the estimated fetal weights derived from these charts to actual birth weight, and determine whether there were gender differences in fetal growth trajectories and immediate birth outcomes.

## Methods

**Fig. 1:**
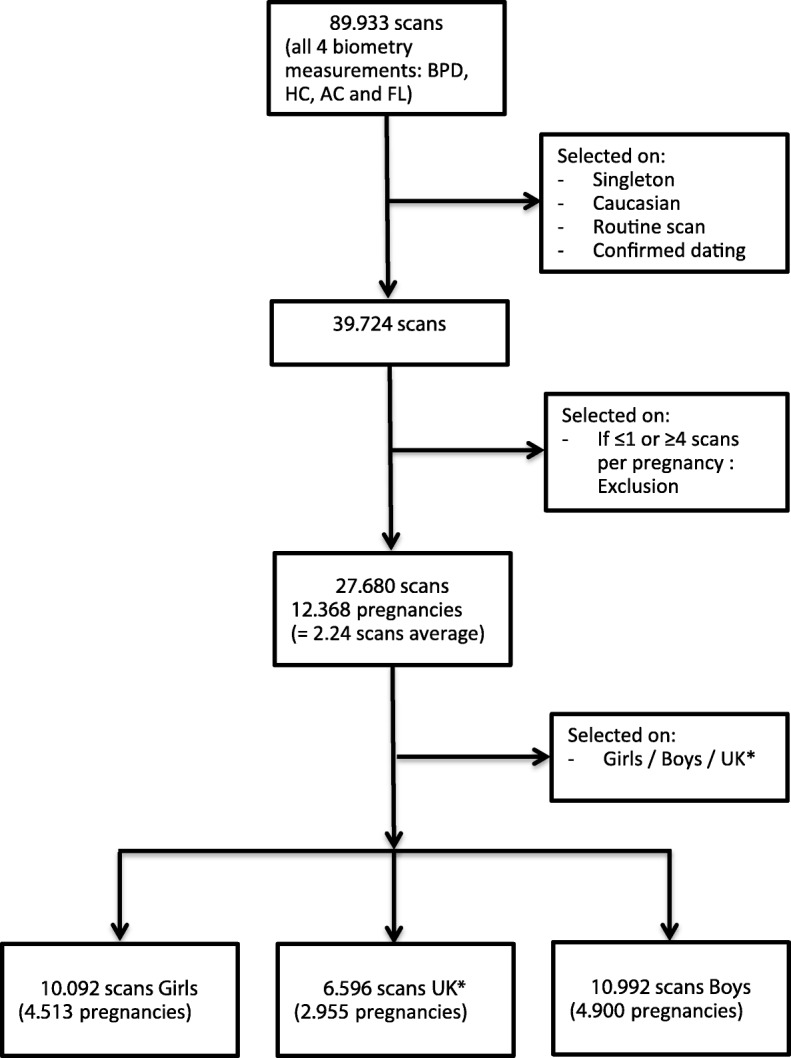
Flowchart on the selection procedure for normal routine fetal ultrasound scans between 2002 and 2012. *UK, unknown gender

This was an observational longitudinal cohort study of first, second, and third trimester fetal biometry ultrasound examinations performed during 2002–2012 in the University Hospital Leuven. The study was approved by the ethics committee of the University Hospitals KU Leuven. The data was selected from the astraia© ultrasound database with the following criteria (Fig. 1): indication “routine fetal growth” (level 1 and 2 ultrasound scanning for fetal anomalies, excluded), singleton pregnancy, ethnicity “Caucasian,” and gestational age confirmed by a crown-rump length (CRL) measurement (3–83 mm) in the first trimester [25]. Only pregnancies with at least two or maximum three scans (first, second, and third trimester) were selected, representing a routine of care scheme for a low-risk population. The measurements were performed with the following ultrasound machines (with time period of usage): Kretz Voluson 730 (2002–2006), ESAOTE Technos (2002–2006), Acuson Sequoia (2002–2007), General Electric Voluson® 730 Expert (GE Healthcare Medical Systems, Kretztechnik, Zipf, Austria, 2007–2012), General Electric Voluson E8 (GE Healthcare Medical Systems, Kretztechnik, Zipf, Austria, 2007–2012). The first three devices were equipped with a 4–8-MHz curved linear array probe. The GE Voluson E730 and GE Voluson E8 used a curved 4–8-MHz volumetric 3D abdominal probe. All growth data were immediately stored in an electronic database (astraia© Software Inc., Munich, Germany). Fetal measurements were based on the following two-dimensional biometric parameters: biparietal diameter (BPD), head circumference (HC), abdominal circumference (AC), and femur length (FL), as designated in the guideline descriptions (Additional file 1) [26]. Only the complete fetal datasets (all four measurements) were analyzed. Neonatal data from the included patients were extracted from their birth files for gestational age at delivery, gender, birth weight, birth length, head circumference, Apgar scores (AS) for the first and fifth minute after birth, umbilical cord arterial pH, and base excess (BE) measurement. Only the gender-specific neonatal datasets were analyzed.

### Statistical analysis

Outliers in BPD, HC, AC, or FL were removed from the data. Generalized Additive Models for Location, Scale and Shape (GAMLSS; www.gamlss.org) was applied to construct the growth curves for all four fetal routine fetal biometry measurements: BPD, HC, AC, and FL, by the use of the R package software [22, 23]. We assessed several distributions: Box-Cox-t, Box-Cox Cole and Green, and Box-Cox power exponential. Goodness-of-fit of the models was assessed with QQ plots, Akaike Information Criteria (AIC), and worm plots. The goodness-of-fit was investigated covering the gestational age 12–40-week period and for substrata of this period. GAMLSS smoothed the antenatal growth curves for BPD, HC, AC, FL, and estimated fetal weight (EFW). For the EFW, the Hadlock-3 formula [Log_10_ EFW = 1.3350.0034 (AC) (FL) + 0.0316 (BPD) + 0.0457 (AC) + 0.1623 (FL)] was used [11]. The 5th, 10th, 50th, 90th, and 95th percentiles were plotted with grid lines. The whole analysis was done three times: for all pregnancies, for boys, and for girls. SAS 9.4 was used for merging the fetal database with the neonatal database and analyzing the neonatal data (Mann-Whitney test).

## Results

Between 2002 and 2012, 89,933 scans were selected. After restricting to a low-risk population, a total of 27,680 scans remained representing 12,368 pregnancies (Fig. 1). The mean maternal BMI was 23.8 kg/m^2^ (std. 4.8), 6.6% of the women smoked. Gender-specific birth datasets could be ascertained in 76.1% of the cases and are outlined in Table 1. In total, we had 4900 boys and 4513 girls, representing respectively 10,992 and 10,092 scans. The mean birth weight, birth length, and head circumference were significantly (*p* < 0.001) different for boys (3450 g, 50.9 cm, 34.9 cm) as compared to girls (3329 g, 50.1 cm, 34.3 cm). A low 1-min AS (≤ 5) was more common in boys (3.8%) as compared to girls (2.9%) (*p* = 0.01) as was a low 5-min AS (≤ 7) for boys (3.2%) compared to girls (2.3%; Table 1) (*p* = 0.009). The arterial umbilical cord pH was lower in boys compared to girls (*p* < 0.001). There was no difference in asphyxia, defined as a pH < 7.10, in boys (0.9%) compared to girls (1.0%, *p* = 0.90), and abnormal BE (< − 10 mEq/L) was the same for both sexes. There was no difference in preterm birth (< 37 weeks) for girls (5.7%) and boys (6.5%, p = 0.14; Table 2) which occurred in 6% of the pregnancies overall. In the preterm group, boys were heavier (*p* = 0.003), longer (*p* = 0.005), and had larger head circumferences (*p* = 0.006). The immediate outcome of AS and pH were also different in boys and girls, although not statistically different due to the smaller preterm group (Table 2). The term group is outlined separately in Additional file 2.

**Table 1:** Neonatal data for boys, girls, and combined in term and preterm pregnancies

	Total (*N* = 9413)	Boys (*N* = 4900, 52.1%)	Girls (*N* = 4513, 47.9%)	*p* value*
GA at birth (wks, 0/7d), mean ± std	39wks2/7 ± 1wks5d	39wks2/7 ± 1wks5d	39wks3/7 ± 1wks5d	0.02
Birth weight (g), mean ± std	3392 ± 512	3450 ± 515	3329 ± 502	< 0.001
Length (cm), mean ± std	50.5 ± 2.4	50.9 ± 2.4	50.1 ± 2.4	< 0.001
HC (cm), mean ± std	34.6 ± 1.6	34.9 ± 1.6	34.3 ± 1.5	< 0.001
1-min AS ≤ 5	3.4%	3.8%	2.9%	0.01
5-min AS ≤ 7	2.8%	3.2%	2.3%	0.009
pH Umb Art, mean ± std	7.27 ± 0.07	7.269 ± 0.072	7.274 ± 0.075	< 0.001
pH < 7.10, mean ± std	1.9% (177), 7.04 ± 0.06	0.9% (82), 7.041 ± 0.059	1.0% (95), 7.042 ± 0.062	0.90
BE < − 10 mEq/L, mean ± std	1.4% (132), − 13.46 ± 4.01	0.8% (71), − 13.8 ± 4.2	0.6% (61), − 13.1 ± 3.7	0.30

Neonatal demographic data available in 9413 (76%) of the selected cases “2002–2012.” *p value represents difference in boys vs girls

*GA* gestational age, *wks* weeks, *d* days, *g* gram, *cm* centimeter, *HC* head circumference, *AS* Apgar scores, *pH* pondus hydrogenium, *Umb Art* umbilical artery, *BE* base excess, *std* standard deviation

**Table 2:** Neonatal data for boys, girls, and combined in preterm (< 37 weeks) pregnancies

	Total (*N* = 576)	Boys (*N* = 317, 55.0%)	Girls (*N* = 259, 45.0%)	*p* value*
GA at birth (wks, 0/7d), mean ± std	34wks6/7 ± 2wks2/7	35wks0/7 ± 2wks1/7	34wks5/7 ± 2wks3/7	0.19
Birth weight (g), mean ± std	2481 ± 637	2553 ± 604	2392 ± 665	0.003
Length (cm), mean ± std	46.3 ± 4.2	46.8 ± 4.1	45.7 ± 4.3	0.005
HC (cm), mean ± std	32.5 ± 2.5	32.8 ± 2.4	32.2 ± 2.6	0.006
1-min AS ≤ 5	8.1%	8.6%	7.4%	0.62
5-min AS ≤ 7	7.9%	8.9%	6.7%	0.32
pH Umb Art, mean ± std	7.29 ± 0.07	7.285 ± 0.070	7.289 ± 0.076	0.50

Neonatal demographic data available in 576 preterm cases “2002–2012.” *p value represents difference in boys vs girls

*GA* gestational age, *wks* weeks *d* days, *g* gram, *cm* centimeter, *HC* head circumference, *AS* Apgar scores, *pH* pondus hydrogenium, *Umb Art* umbilical artery, *std* standard deviation

**Fig. 2:**
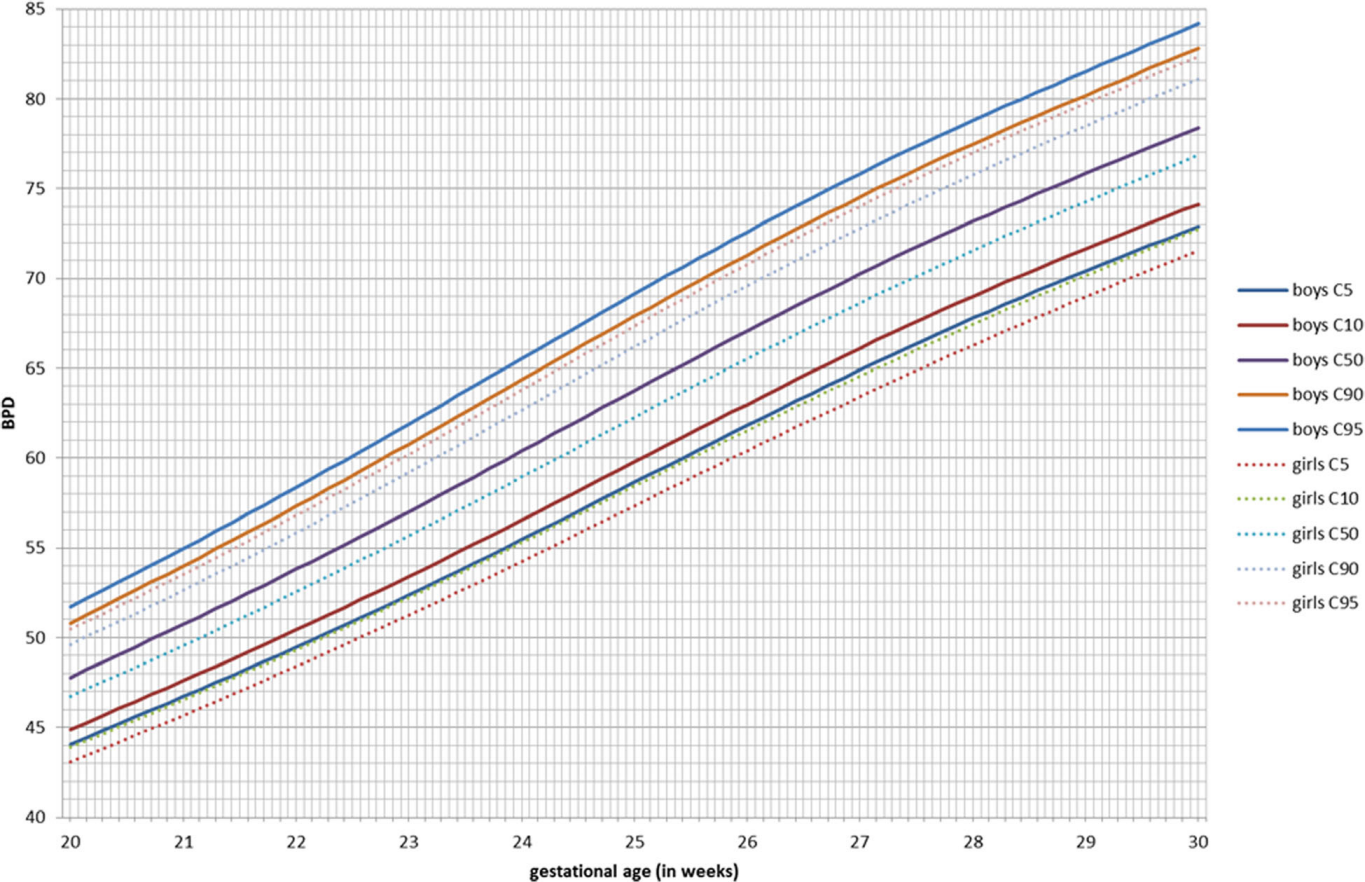
Biparietal diameter (BPD) in millimeters for boys and girls from 20 to 30 weeks of gestation for percentiles 5, 10, 50, 90, and 95

**Fig. 3:**
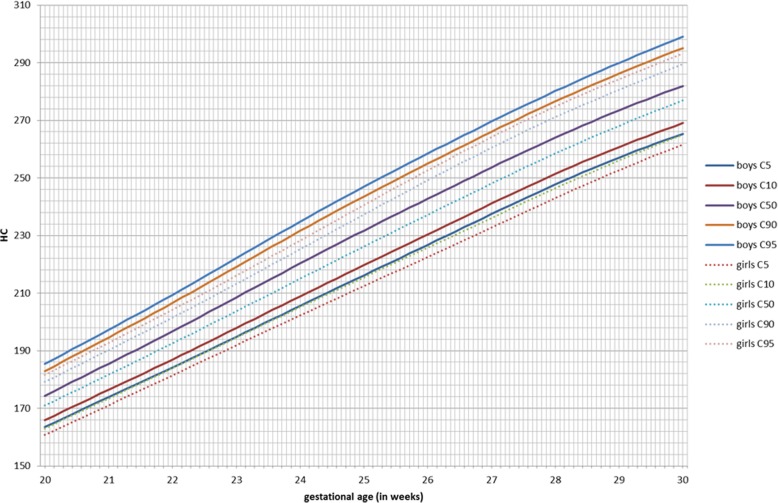
Head circumference (HC) in millimeters for boys and girls from 20 to 30 weeks of gestation for percentiles 5, 10, 50, 90, and 95

**Fig. 4:**
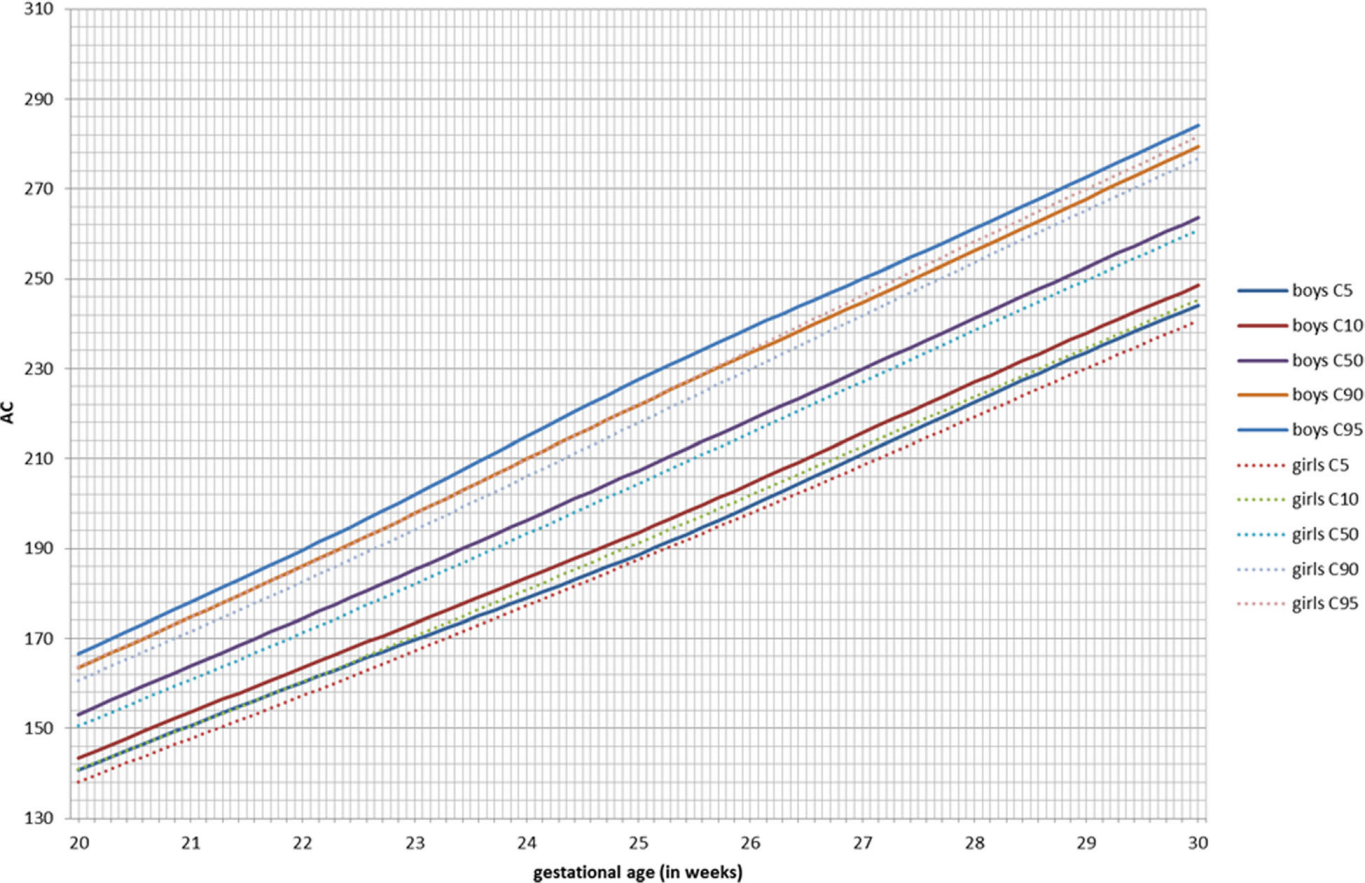
Abdominal circumference (AC) in millimeters for boys and girls from 20 to 30 weeks of gestation for percentiles 5, 10, 50, 90, and 95

**Fig. 5:**
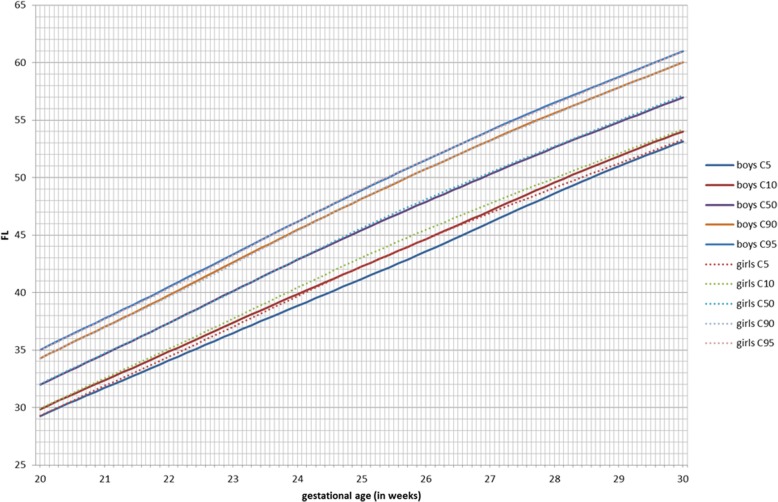
Femur length (FL) in millimeters for boys and girls from 20 to 30 weeks of gestation for percentiles 5, 10, 50, 90, and 95

GAMLSS longitudinal fetal antenatal growth curves for BPD, HC, AC, and FL from 12 to 40 weeks were developed for boys, girls, and combined (Additional file 3). For each parameter, the 5th, 10th, 50th, 90th, and 95th centiles were constructed. Actual values for these centiles and grid curves are outlined in Additional file 4. Comparing the two gender growth trajectories and their percentiles, for BPD, there was a significant (*p* < 0.001) difference for all percentiles in boys having higher BPD measurements (Fig. 2, Table 3). At 24 weeks, the 50th percentile BPD for boys (60.4 mm) is significantly higher as compared to girls (58.9 mm, *p* < 0.001; Additional file 5). This corresponds to a difference of three gestational days. The boys’ 5th percentile aligns with the 10th percentile of the girls, and the 90th percentile aligns with the 95th percentile of the girls. For HC, these differences were even more pronounced (*p* < 0.001; Additional file 5). The prenatal difference of HC of boys at the 95th percentile increases to + 6.5 mm at 35 weeks, but it is already present at 2 weeks of gestation (+ 3.8 mm; Fig. 3, Table 4). The neonatal head circumference confirmed this difference of + 6 mm as being significant between boys and girls (*p* < 0.001; Table 1). Generally, prenatal AC measurements were significantly higher in boys than in girls, but less demonstrable across the total gestational period than for BPD and HC (Fig. 4). For FL, there was no significant difference between boys and girls in their antenatal growth percentiles (Fig. 5). The EFW was different in boys throughout the gestational age at different percentiles compared to girls, except for the 40 weeks measurement (Table 5). Girls reach the 500 g EFW 1 day later (22wks3/7) as compared to the boys (22wks2/7; Additional file 5). At the 50th percentile at 24 weeks, boys are estimated to be 21 g heavier compared to girls (*p* = 0.02; Additional file 5).

**Table 3:** BPD reference values for boys and girls from 12–40 weeks

GA	Boys (BPD)					Girls (BPD)				
	C5	C10	C50	C90	C95	C5	C10	C50	C90	C95
12	16.7	17.3	19.4	21.6	22.3	16.5	17.1	19.2	21.3	21.9
13	20.4	21.0	23.2	25.6	26.3	20.1	20.7	22.9	25.1	25.7
14	24.3	25.0	27.3	29.7	30.4	24.0	24.6	26.8	29.1	29.7
15	28.2	28.9	31.3	33.8	34.5	27.8	28.5	30.8	33.1	33.7
16	32.0	32.6	35.1	37.6	38.3	31.4	32.1	34.5	36.8	37.5
17	35.3	36.0	38.5	41.0	41.8	34.7	35.4	37.9	40.3	41.1
18	38.3	39.0	41.6	44.3	45.1	37.8	38.5	41.0	43.6	44.4
19	41.3	42.0	44.7	47.6	48.4	40.5	41.3	44.0	46.7	47.5
20	44.0	44.9	47.8	50.8	51.7	43.1	43.9	46.7	49.6	50.5
21	46.7	47.6	50.7	54.0	55.0	45.7	46.5	49.6	52.6	53.5
22	49.5	50.4	53.8	57.3	58.4	48.4	49.3	52.5	55.8	56.8
23	52.4	53.4	57.0	60.8	61.9	51.2	52.2	55.7	59.2	60.2
24	55.5	56.6	60.4	64.3	65.5	54.2	55.3	58.9	62.7	63.8
25	58.6	59.8	63.8	67.9	69.1	57.3	58.4	62.3	66.2	67.3
26	61.8	63.0	67.1	71.3	72.6	60.4	61.5	65.5	69.6	70.8
27	64.9	66.1	70.2	74.5	75.8	63.4	64.6	68.6	72.8	74.0
28	67.8	69.0	73.2	77.5	78.8	66.3	67.4	71.5	75.8	77.0
29	70.4	71.6	75.8	80.2	81.5	69.0	70.2	74.3	78.5	79.7
30	72.9	74.1	78.4	82.8	84.2	71.6	72.7	76.9	81.1	82.4
31	75.3	76.6	81.0	85.5	86.9	74.0	75.2	79.4	83.6	84.9
32	77.6	78.9	83.3	87.9	89.4	76.2	77.4	81.7	86.0	87.3
33	79.5	80.9	85.4	90.1	91.6	78.1	79.4	83.7	88.2	89.5
34	81.2	82.6	87.3	92.1	93.7	79.8	81.1	85.6	90.2	91.6
35	82.5	84.0	88.9	93.9	95.5	81.3	82.7	87.3	92.1	93.5
36	83.6	85.1	90.3	95.6	97.3	82.7	84.1	88.9	93.9	95.3
37	84.6	86.2	91.7	97.4	99.2	84.1	85.5	90.5	95.6	97.2
38	85.7	87.4	93.2	99.3	101.2	85.5	87.0	92.1	97.4	99.0
39	87.0	88.8	94.9	101.2	103.3	86.9	88.4	93.7	99.3	100.9
40	88.4	90.3	96.6	103.2	105.3	88.2	89.8	95.3	101.1	102.8

Reference values in millimeters for fetal biparietal diameter (BPD) for boys and girls for each gestational week for the median and 5th, 10th, 90th, and 95th centiles

*GA* gestational age

**Table 4:** HC reference values for boys and girls from 12 to 40 weeks

GA	Boys (HC)					Girls (HC)				
	C5	C10	C50	C90	C95	C5	C10	C50	C90	C95
12	61.3	63.3	70.3	77.4	79.4	61.9	63.6	69.7	76.1	78.1
13	74.4	76.5	83.8	91.1	93.2	74.3	76.1	82.6	89.5	91.6
14	88.2	90.3	97.8	105.3	107.5	87.3	89.2	96.1	103.3	105.4
15	101.9	104.1	111.6	119.2	121.4	100.6	102.6	109.6	117.0	119.2
16	115.2	117.4	124.9	132.6	134.8	113.6	115.6	122.8	130.4	132.6
17	128.0	130.2	137.8	145.6	147.8	126.3	128.3	135.7	143.3	145.6
18	140.3	142.5	150.3	158.2	160.5	138.5	140.7	148.2	156.0	158.3
19	152.2	154.5	162.6	170.7	173.1	150.1	152.3	160.0	168.0	170.4
20	163.4	165.8	174.3	182.9	185.4	160.7	163.0	171.0	179.2	181.6
21	173.9	176.4	185.5	194.6	197.3	171.1	173.4	181.7	190.2	192.8
22	184.2	187.0	196.8	206.6	209.5	181.5	183.9	192.6	201.5	204.2
23	194.8	197.9	208.5	219.1	222.2	191.9	194.5	203.7	213.2	216.0
24	205.6	208.9	220.3	231.6	234.8	202.3	205.1	215.0	225.2	228.3
25	216.2	219.7	231.7	243.6	247.0	212.5	215.5	226.2	237.3	240.6
26	226.8	230.4	242.8	255.0	258.5	222.5	225.8	237.2	249.1	252.7
27	237.4	241.1	253.6	266.0	269.6	232.7	236.1	248.0	260.5	264.2
28	247.7	251.4	264.0	276.5	280.2	243.0	246.4	258.5	271.1	274.8
29	257.1	260.7	273.4	286.2	290.0	252.7	256.1	268.1	280.6	284.3
30	265.3	269.0	282.0	295.1	299.0	261.6	265.0	277.0	289.5	293.2
31	273.0	276.8	290.0	303.7	307.7	269.6	273.1	285.5	298.3	302.1
32	280.2	284.0	297.6	311.9	316.3	276.5	280.2	293.2	306.7	310.8
33	286.5	290.4	304.4	319.4	324.1	282.6	286.4	300.1	314.4	318.7
34	292.1	296.1	310.3	326.0	331.0	288.0	292.0	306.2	321.1	325.5
35	297.7	301.6	315.9	332.2	337.5	292.6	296.7	311.3	326.5	331.0
36	302.9	306.8	321.2	338.2	343.9	296.6	300.7	315.5	330.9	335.5
37	307.3	311.3	326.1	344.1	350.4	301.0	305.2	320.1	335.8	340.4
38	311.9	316.0	331.1	350.4	357.5	306.5	310.7	325.9	341.7	346.4
39	317.3	321.4	336.7	357.1	365.2	312.8	317.1	332.4	348.5	353.3
40	323.5	327.6	342.9	364.2	373.1	319.4	323.7	339.2	355.4	360.2

Reference values in millimeters for fetal head circumference (HC) for boys and girls for each gestational week for the median and 5th, 10th, 90th, and 95th centiles

*GA* gestational age

**Table 5:** EFW reference values for boys and girls from 12 to 40 weeks

GA	Boys (EFW)					Girls (EFW)				
	C5	C10	C50	C90	C95	C5	C10	C50	C90	C95
12	50	51	57	63	65	49	50	55	61	63
13	60	62	69	78	81	61	62	69	76	79
14	77	80	89	101	105	79	82	90	101	104
15	104	107	120	136	142	103	106	117	131	136
16	131	136	152	172	179	128	132	147	165	171
17	159	165	185	208	216	159	164	183	206	213
18	199	205	230	260	269	198	204	229	257	266
19	248	256	287	324	336	242	250	281	316	328
20	298	308	346	391	406	290	299	337	380	394
21	352	364	410	465	482	342	354	398	450	466
22	414	429	486	552	574	402	416	470	531	551
23	488	506	576	658	685	475	493	557	631	655
24	574	596	681	781	813	561	582	660	750	779
25	671	698	799	917	955	658	684	777	884	919
26	779	810	928	1065	1110	765	795	905	1032	1073
27	901	938	1073	1230	1280	883	918	1046	1193	1241
28	1038	1080	1233	1410	1467	1014	1054	1201	1369	1424
29	1188	1235	1407	1605	1669	1158	1204	1369	1558	1620
30	1348	1400	1593	1814	1886	1314	1366	1550	1761	1831
31	1511	1571	1788	2035	2116	1478	1536	1743	1979	2059
32	1672	1739	1985	2265	2357	1638	1704	1937	2206	2298
33	1825	1902	2182	2504	2610	1788	1863	2128	2437	2544
34	1970	2058	2380	2752	2876	1927	2012	2314	2671	2796
35	2108	2209	2577	3007	3152	2060	2156	2500	2912	3059
36	2244	2358	2775	3267	3434	2193	2301	2689	3163	3336
37	2379	2506	2974	3530	3720	2330	2452	2887	3428	3629
38	2513	2655	3174	3795	4009	2475	2610	3095	3707	3940
39	2647	2802	3373	4060	4299	2626	2774	3310	3999	4265
40	2776	2946	3570	4325	4591	2780	2943	3531	4300	4605

Reference values in grams for estimated fetal weight (EFW) for boys and girls for each gestational week for the median and 5th, 10th, 90th, and 95th centiles

*GA* gestational age

## Discussion

In this study, we have constructed antenatal growth and estimated fetal weight charts, with a strict and clearly defined selection protocol in a normal Caucasian population and separately for boys and girls. Boys have significantly larger late -second and third trimester HC, BPD, and AC measurements than girls. For FL, there are no differences. The implication of these findings is that a boy and a girl at exactly 24 weeks of gestation might, based on the current late second trimester dating protocols with head measurements, be assigned a gestation as much as 3-day difference and an EFW difference of 21 g at 24 weeks favoring the boys. These antenatal differences were confirmed at birth with boys being significantly heavier, longer, and having larger head circumferences as compared to girls. The 1- and 5-min AS and cord pH was lower in boys. The dating and weight estimation differences could potentially be taken into account in determining prenatal and immediate perinatal viability management in terms of timing the administration of maternal steroids for fetal lung maturation, decisions for delivery, and possible resuscitation. Also, in the post-term period management in pregnancy, these gender differences could also potentially influence decisions including the timing of labor inductions, affecting an even larger population. Consequently, if second trimester dating of the pregnancy has been undertaken, girls are potentially put at risk of stillbirth in the post-term period by assuming the gestational maturity to be less than it is [27].

In one cross-sectional study, a difference has been shown between fetal head measurements for both boys and girls, although the curves were constructed with the older linear regression models [28]. They also confirmed the birth weight difference but did not report information on neonatal head circumference or other outcomes (AS, cord pH). Another unselected multi-ethnic combined cross-sectional and longitudinal population study also found differences in fetal head and abdomen measurements using statistical methods current at that time; however, no birth outcomes were available [29].

While it has been demonstrated that gestation-specific neonatal outcomes are worse in boys than in girls [9, 10], what had not been previously appreciated in a routine population is that boys have lower Apgar scores at both 1 and 5 min and lower cord pH values at delivery than girls. These results underline male vulnerability in the perinatal period. In a recent published elegant report on neonatal outcome in appropriately grown term babies, gender differences were demonstrated in terms of lower Apgar scores at 5 min and higher rates of instrumental deliveries for failure to progress in labor for boys [30]. This concerned a multi-ethnic retrospective cohort from one center and birth data specified for both genders. They demonstrated a birth weight difference of 135 g at term, comparing closely with the 121 g that we report, but their data lacked other anthropometric data (birth length and head circumference) and antenatal growth data. It is of course possible that neonatal outcomes are worse because immediate birth outcomes are worse. Whether this is an attribute of being male per se, or some effect of fetal size on delivery, cannot be explained from their results or ours. We can demonstrate that the gender differences in fetal anthropometry starting from 20 weeks onwards affect fetal dating and the estimated fetal weight. In our preterm sub-analysis, the birth weight differences between boys and girls are also present in absolute mean differences (∆birth weight 161 g, ∆birth length 0.8 cm, ∆HC 0.6 cm), and there are noticeable differences between AS and umbilical cord pH (Table 2), although not statistically significant due to smaller numbers. One hypothesis is that either the differences in biometry are relatively more important in the (full-grown) male fetus interacting with maternal pelvic limitations causing more labor dystocia for boys, and hence lower AS. Alternatively, other fetal gender-specific factors can influence the birth process and compromising the immediate birth outcomes. Gender-specific body composition at birth has been reported, where the male infant has more fat mass and lean body mass than the female infant, especially in well-nourished mothers [31]. This phenomenon has been associated with gender-different intrauterine physical adaptations to an enhanced nutrient supply from the mother. The male infant body composition has been more subject to maternal influences as higher pre-gestational BMI and excessive gestational weight gain [32]. Lastly, the lung maturation of the male fetus proceeds slower than in the female fetus, possibly contributing to a higher rate of low AS in the term grown fetus. In animal studies, lung fluid secretion is inhibited and the lung fluid absorption initiated by adrenalin infusions at birth [33]. And preterm asphyxiated male infants have lower adrenaline levels than female infants, again putting the boys at higher risk [34]. Whether in the term infant this will be similar is unknown.

### Strength and weakness

Our antenatal growth curves are unique in that all four fetal growth parameters (BPD, HC, AC, and FL) were measured in standardized circumstances in accordance with international guidelines [26]. Longitudinal growth charts were constructed for each parameter, with the WHO advocated GAMLSS method used [22, 23]. GAMLSS can combine longitudinal data with a cross-sectional component and can construct centiles in a way that they are constrained and do not cross. Further, in using the GAMLSS analysis statistics, one could, by synchronizing the statistical methods of the WHO, align the biometry measurements with the neonatal and pediatric charts [22, 23]. With the available neonatal data, we could discriminate different growth curves for boys and girls for all four fetal growth parameters and hence the EFW. Since the introduction of ultrasound in antenatal care, many reports on fetal growth curves have been published [11–21]. Recognizing pathological fetal growth depends on reliable, standardized growth curves [35]. Discrepancies between the curves have often been attributed to the differences in methodology and population selection [36]. A recent report reviewed fetal growth charts, demonstrating the wide variations of methodologies on how these charts have been constructed concluding that there were many grounds for bias in the growth curves that are currently used [37]. Particularly in “inclusion/exclusion criteria,” “ultrasound quality control measures,” and “gestational dating protocols,” many ambiguities existed. Standardization of the methodologies with a checklist was recommended to define a high-quality study [37]. When we compare our growth charts to the requirements, these would be compliant for the combination of a high-quality control score, longitudinal design, sample size, and the fact that all four parameters (BPD, HC, AC, and FL) were examined (Additional file 6). All growth measurements were reviewed by certified staff members, judging all the scanned images as to whether they adhered to the protocol described. We also incorporated a strict protocol on pregnancy dating. Only pregnancies that had a first trimester confirmation scan on gestational age were included: crown-rump length (CRL) measurement between 3 and 83 mm (gestational age ≥ 5^+0^ and < 14^+0^ weeks) [4, 25]. In Belgium, in routine obstetrical care, every pregnant woman will be offered a first, second, and third trimester ultrasound scan with fetal growth measurements. In many countries, the third trimester scan is not part of the routine care for low-risk pregnancies [38]. Also, to measure the four fetal growth parameters in the first trimester is not a routine care and allowed us to define “fetal growth” through serial measurements, instead of “fetal size,” as defined through cross-sectional measurements [12–19, 39]. Furthermore, we were able to eliminate aberrant fetal growth and extreme maternal influences by excluding fetal anomalies (level 1 and 2 indications) and including only the mothers enrolled to a routine obstetric care scheme [40]. Finally, a population-based cohort was generated with a significant sample size over a period of 11 years. The description of a routine population could also be supported by our neonatal data. Neonatal data was complete for 76% in our cohort. The rate of premature birth was 6%, which is consistent with the European nationally accepted norms. In our population selection, we further customized the charts for one maternal and one fetal factor. We selected on ethnicity “Caucasian” and the fetal gender. Other ethnicity-derived customized growth curves have arisen in response to the early reference charts from mainly Europe and the USA [18, 19]. Ethnicity was reported to have a discriminative influence on fetal growth [24, 41]. The aim of the INTERGROWTH-21st study was to construct prescriptive instead of descriptive curves using the same statistical methods as used in our study (GAMLLS) [42]. The study population comprised 35% of the pregnant population, recruited highly selected healthy, educated (> 75% of a local level), non-obese (BMI 18–30 kg/m^2^), non-smoking women, 18–35 years of age and recruited in selected institutes. This highly qualitative study (Additional file 6) represents a fascinating investigation of the physiology of fetal growth, concluding that optimal growth potential can be attained irrespective of the ethnicity in a selected population, which is in contradiction with the previous studies. Unfortunately, it lacks information on fetal gender differences; not all measurements were longitudinal, and the derived charts are by their selective nature manifestly not representative of a general population, regardless of the ethnicity concerned. Our current study adds these advantages. Girls and boys both have different neonatal growth curves, assuming there is a discriminative effect of the gender on their growth trajectories. In more than three quarters of our cohort, complete neonatal data was registered, including gender registration. Therefore, we focused on developing two separate fetal growth charts, both for boys and girls. Comparing the extremes of growth (< p5 and > p95), the female fetus is considered wrongfully small or non-macrosomic and the male fetus vice versa when compared to the INTERGROWTH-21st curves (Table 6). Fetal gender, unlike maternal ethnicity, is not commonly known in the first trimester but it is from the 20 weeks’ scan onwards (“anomaly” scan). From a clinical point of view, it seemed therefore relevant to start discriminating these curves from 20 weeks of gestation onwards.

**Table 6:** Cross-sectional gestational age comparison of INTERGROWTH-21st and gender-specific (M/F) fetal head measurements at 5th and 95th percentiles

GA	C5	IG	F	M	C95	IG	F	M
BPD								
14	24.2	26.8	24.0	24.3	30.2	32.5	29.7	30.4
15	28.2	29.6	27.8	28.2	34.5	35.6	33.7	34.5
16	31.5	32.5	31.4	32.0	38.0	38.8	37.5	38.3
20	43.5	44.7	43.1	44.0	51.3	52.2	50.5	51.7
24	54.7	57.0	54.2	55.5	64.8	65.7	63.8	65.5
30	72.2	73.9	71.6	72.9	83.6	84.0	82.4	84.2
34	80.6	82.4	79.8	81.2	92.9	93.4	91.6	93.7
37	84.7	86.7	84.1	84.6	98.2	98.4	97.2	99.2
HC								
14	87.6	88.7	87.3	88.2	108.0	107.1	105.4	107.5
15	101.8	100.6	100.6	101.9	123.3	120.1	119.2	121.4
16	113.9	112.6	113.6	115.2	135.3	133.2	132.6	134.8
20	161.8	160.2	160.7	163.4	184.4	184.7	181.6	185.4
24	203,9	205.4	202.3	205.6	233.3	232.7	228.3	234.8
30	263.4	263.2	261.6	265.3	297.2	293.6	293.2	299.0
34	290.2	291.5	288.0	292.1	328.9	324.7	325.5	331.0
37	302.9	305.7	301.0	307.3	346.0	342.7	340.4	350.4

Reference values in millimeters for fetal biparietal diameter (BPD) and head circumference (HC) for gestational landmark weeks for the 5th and 95th centiles. Total group (C5 and C95), INTERGROWTH-21st (IG) and fetal gender current study (F/M)

*GA* gestational age (in weeks)

Some limitations on constructing these charts have to be addressed. The study was performed in a university teaching hospital, a large tertiary referral center, not necessarily reflecting a routine setting. This center, on the other hand, also has a regional remit for routine obstetric care for low-risk pregnancies, but the included cases were not selected on maternal morbidity nor on parental characteristics. Some maternal characteristics (e.g., smoking occurred in 6.6%) were not excluded in the selected cohort, deliberately to prevent “super-normalization” of the cohort. But artificial conception was excluded for intracytoplasmic sperm injection, since this is a level 1 ultrasound indication. Finally, it is expected that within this large time period, some women with subsequent pregnancies were included more than once for this cohort.

### Implications for clinical practice

Our fetal growth curves for the Caucasian population resemble predictive growth curves with the gender specified which can discern aberrant from normal fetal growth. The longitudinal aspect and large cohort, covering the full trimesters, have not been reported before in the Caucasian population. The neonatal data gave us the opportunity to customize for the fetal gender. There was a marked difference between fetal boys and girls in their growth trajectory for fetal head measurements and to a lesser extent the abdominal circumferences. Also for the estimated fetal weight, there was a difference. This gender differentiation is important in antenatal and perinatal care. Prenatal ultrasound is used not only to define fetal growth, but also gestational age. Both growth and fetal age are important in defining the time point of fetal viability and the optimization of the timing of obstetrical interventions, e.g., medical elective birth or administration of corticosteroids for fetal lung maturation in cases of threatened premature birth. Second trimester dating depends on fetal growth parameters and particular on the fetal head measurement. Our results suggest a gender-specific approach in counseling future parents on important issues when fetal viability starts and when is the best time point to start obstetrical interventions.

The gender differences are further demonstrated by the immediate birth outcomes for males: different anthropometry (heavier, longer, and bigger heads), lower AS, and lower cord pH. The significant lower AS and umbilical cord pH in boys underline the fetal male vulnerability, although in the asphyxia group (pH < 7.10), there was no predominance by males, stating that boys do not have a higher risk of acidemia at birth in a routine population. Therefore, one can argue on the clinical importance of the pH findings (and perhaps also the AS) in our study.

## Conclusion

In summary, we present fetal growth curves with the latest statistical tools in a large, routine pregnant population with state-of-the-art ultrasound technology. The data covers the pregnancy period from 12 weeks onwards, and there were differences between boys and girls for the fetal head and fetal abdomen measurements and the estimated fetal weight. Also, the immediate neonatal outcome demonstrated gender differences favoring the girls. This could give caretakers the opportunity to take into account a gender-tailored approach in life decision care both at the margins of viability and post-term.

## Additional files

Additional file 1: Ultrasound protocol guidelines [26]. (DOCX 12 kb)

Additional file 2: **Table S1.** Neonatal data for boys, girls and combined in term (≥ 37 weeks) pregnancies. (DOCX 23 kb)

Additional file 3: Boys vs Girls combined scatterplots and curves. (DOCX 816 kb)

Additional file 4: Boys vs Girls combined gridcurves and reference values. (DOCX 4051 kb)

Additional file 5: Boys vs Girls combined centiles and real values. (DOCX 35 kb)

Additional file 6: **Table S2.** Fetal charts characteristics, quality control. (DOCX 17 kb)

## Data Availability

The datasets used and/or analyzed during the current study are available from the corresponding author on reasonable request.
