# Supplementary material for: Sex differences in fetal growth and immediate birth outcomes in a low-risk Caucasian population
Source: Biol Sex Differ. 2019 Sep 9;10:48. doi: 10.1186/s13293-019-0261-7 (PMC6734449; doi:10.1186/s13293-019-0261-7)
Supplement: Supplementary file 1 — Ultrasound protocol guidelines [26]. (DOCX 12 kb) [file 13293_2019_261_MOESM1_ESM.docx]

**Ultrasound protocol guidelines**

All operators performed the measurements as single observations in accordance to the following guidelines, subsequently defined by the International Society for Ultrasound in Obstetrics and Gynecology (ISUOG, [www.isuog.org](http://www.isuog.org)).^26^ The **BPD** measurement was performed in the transverse plane of the skull at the level where the continuous midline echo is interrupted anteriorly by the *septum cavum pellucidum* and centrally by the thalami. Ideally, an angle of insonation of 90° on the midline echo is used. Measurements were performed from the proximal edge of the skull closest to the transducer to the proximal edge of the deep border (i.e. outer-inner edge of skull) with the calipers. The **HC** measurement was performed in the same plane. If the ultrasound equipment had an ellipse measurement capacity, then the HC could be measured directly by placing the ellipse around the outer edge of the skull. Alternatively, the HC could be calculated from the BPD and the Occipito-Frontal Diameter (OFD) by using the equation HC=1.62x(BPD+OFD). This formula required the placement of the calipers for BPD at leading edges and for OFD middle of the bone echo at forehead and occiput. The **AC** measurement was performed in the transverse plane of the fetal abdomen at the level of the stomach, as circular as possible. The umbilical vein should be visible at the level of the portal sinus. The kidneys should not be visible. The AC was measured at the outer surface of the skin line using ellipse calipers or it could be calculated from linear measurements made perpendicular to each other, usually the Anterior-Posterior Abdominal Diameter (APAD) and the Transverse Abdominal Diameter (TAD). To measure the APAD the calipers were placed at the outer borders of the body outline from the posterior aspect (skin covering the spine) to the anterior abdominal wall. To measure the TAD the calipers were placed on the outer borders of the body outline, across the abdomen at the widest point. The AC was calculated by a direct ellipse measurement or using the formula AC=1.57(APAD+TAD). The **FL** was optimally imaged with both ends of the ossified metaphyses clearly visualized. The longest axis of the ossified diaphysis was measured. An angle of 45-90° between the insonating ultrasound beam and the femur were aimed for. The calipers were placed at the ends of the ossified diaphysis if it is visible avoiding the artificial triangle spurs that appear to extend the length of the femoral ends.

References

26. ISUOG Prenatal Ultrasound Screening Taskforce. Practice guidelines for performance of the routine mid-trimester fetal ultrasound scan. <http://www.isuog.org/NR/rdonlyres/EA865840-6CA3-45AC-9E99-FBAF775119A9/0/PrenatalUS_GL_Final.pdf>
