# Supplementary material for: Sex differences in fetal growth and immediate birth outcomes in a low-risk Caucasian population
Source: Biol Sex Differ. 2019 Sep 9;10:48. doi: 10.1186/s13293-019-0261-7 (PMC6734449; doi:10.1186/s13293-019-0261-7)
Supplement: Supplementary file 2 — Table S1. Neonatal data for boys, girls and combined in term (≥ 37 weeks) pregnancies. (DOCX 23 kb) [file 13293_2019_261_MOESM2_ESM.docx]

**Additional file 2 Table S1.** Neonatal data for boys, girls and combined in term (≥ 37weeks) pregnancies.

|  | **Total**  **(N= 8837)** | **Boys**  **(N= 4583, 51.9%)** | **Girls**  **(N= 4254, 48.1%)** | **P-value*** |
| --- | --- | --- | --- | --- |
|  |  |  |  |  |
| **GA at birth (wks,0/7d), mean ± std** | 39w4/7 ± 1w1/7 | 39w4/7 ± 1w1/7 | 39w5/7 ± 1w1/7 | 0.009 |
|  |  |  |  |  |
| **Birth weight (g), mean ± std** | 3450 ± 444 | 3510 ± 448 | 3385 ± 431 | <0.001 |
|  |  |  |  |  |
| **Length (cm), mean ± std** | 50.7 ± 2.0 | 51.1 ± 2.0 | 50.3 ± 2.0 | <0.001 |
|  |  |  |  |  |
| **HC (cm), mean ± std** | 34.7 ± 1.4 | 35.0 ± 1.5 | 34.4 ± 1.4 | <0.001 |
|  |  |  |  |  |
| **1 minute AS ≤5** | 3.1% | 3.5% | 2.6% | 0.01 |
|  |  |  |  |  |
| **5 minute AS ≤7** | 2.4% | 2.8% | 2.0% | 0.02 |
|  |  |  |  |  |
| **pH Umb Art, mean ± std** | 7.27 ± 0.07 | 7.268 ± 0.072 | 7.273 ± 0.075 | 0.07 |

Additional Table 1. Neonatal demographic data available in 8837 term cases ‘2002-2012. GA = gestational age; wks = weeks; d = days; g = gram; cm = centimeter; HC = Head Circumference; AS = Apgar Scores; pH = pondus Hydrogenium; Umb Art = Umbilical artery; Std. = Standard Deviation. * P-value represents difference Boys vs Girls.

**e-Table 2.** Fetal charts characteristics.

| **Study** | **Year** | **Country** | **N° women** | **N° scans** | **Weeks** | **Measurement** | **Design** | **Data Collection** | **Research?** | **Quality score (%)*** |
| --- | --- | --- | --- | --- | --- | --- | --- | --- | --- | --- |
| **Chitty et al^13-15^** | 1994 | UK | 594-649 | 425-649 | 12-42 | BPD,HC,AC,FL | C1 | Prospective | Yes | 78 |
| **Johnsen et al^20^** | 2006 | NO | 650 | 2489-2589 | 10-42 | BPD,HC,AC,FL | LL | Prospective | Yes | 67 |
| **Kurmanavicius et al^16,17^** | 1999 | CH | 6557 | 5462-6217 | 12-42 | BPD,HC,AC,FL | C1 | Retrospective | No | 65 |
| **Leung et al^19^** | 2008 | CN | 709 | 679-708 | 12-40 | BPD,HC,AC,FL | C1 | Prospective | Yes | 70 |
| **Paladini et al^18^** | 2005 | IT | 626 | 623-625 | 16-40 | BPD,HC,AC,FL | C1 | Prospective | Yes | 65 |
| **Snijders & Nicolaides^12^** | 1994 | UK | 1040 | 1040 | 14-40 | BPD,HC,AC,FL | C1 | Retrospective | No | 61 |
| **Verburg et al^21^** | 2008 | NL | 3760^ǂ^ | 20.277-22.271 | 10-40 | BPD,HC,AC,FL | LL | Prospective | Yes | 75 |
| ***Galjaard et al*** | *2015* | *BE* | *12.368* | *27.680* | *12-40* | *BPD,HC,AC,FL* | *LL* | *Retrospective* | *No* | *75** |

e-Table 2. Characteristics of the highest ranked fetal growth studies (*as categorized by Ioannou C et al. BJOG 2012; 119:1425-1439)^37^. ^ǂ^= actual number of women analyzed after exclusion. C1 = cross-sectional -each fetus measured ones-. L = Longitudinal data -each fetus measured longitudinal-. UK = United Kingdom; NO = Norwegian; CH = Suisse; CN = China; IT = Italy; NL = the Netherlands; BE = Belgium.

References

12. Snijders RJM and Nicolaides KH. Fetal biometry at 14-40 weeks’ gestation. Ultrasound Obstet Gynecol 1994; 4:34-48.

13. Chitty LS, Altman DG, Henderson A, et al. Charts of fetal size: 2. Head measurements. Br J Obstet Gynaecol 1994; 101:35-43.

14. Chitty LS, Altman DG, Henderson A, et al. Charts of fetal size: 3. Abdominal measurements. Br J Obstet Gynaecol 1994; 101:125-31.

15. Chitty LS, Altman DG, Henderson A, et al. Charts of fetal size: 4. Femur length. Br J Obstet Gynaecol 1994; 101:132-5.

16. Kurmanavicius J, Wright EM, Royston P, et al. Fetal ultrasound biometry: 1. Head reference values. Br J Obstet Gynaecol 1999; 106:126-35.

17. Kurmanavicius J, Wright EM, Royston P, et al. Fetal ultrasound biometry: 2. Abdomen and femur length reference values. Br J Obstet Gynaecol 1999; 106:136-43.

18. Paladini D, Rustico M, Viora E, et al. Fetal size for the Italian population. Normative curves of head, abdomen and long bones. Prenat Diagn 2005; 25:456-64.

19. Leung TN, Pang MW, Daljit SS, et al. Fetal biometry in ethnic Chinese: biparietal diameter, head circumference, abdominal circumference and femur length. Ultrasound Obstet Gynecol 2008; 31:321-7.

20. Johnsen SL, Wilsgaard T, Rasmussen S, et al. Longitudinal reference ranges for estimated fetal weight. Acta Obstet Gynecol Scand 2006; 85:286-97.

21. Verburg BO, Steegers EA, De Ridder M, et al. New charts for ultrasound dating of pregnancy and assessment of fetal growth: longitudinal data from a population-based cohort study. Ultrasound Obstet Gynecol 2008; 31:388-96.

37. Ioannou C, Talbot K, Ohuma E, et al. Systematic review of methodology used in ultrasound studies aimed at creating charts of fetal size. BJOG 2012; 119:1425-1439.
