# Supplementary material for: Sex differences in fetal growth and immediate birth outcomes in a low-risk Caucasian population
Source: Biol Sex Differ. 2019 Sep 9;10:48. doi: 10.1186/s13293-019-0261-7 (PMC6734449; doi:10.1186/s13293-019-0261-7)

## Additional file 3. Scatterplots and Curves: Total, Boys & Girls

**Totalgroup**

BiParietalDiameter (BPD), *Total group*


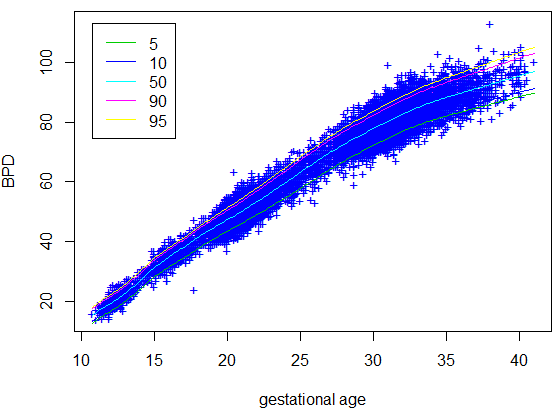


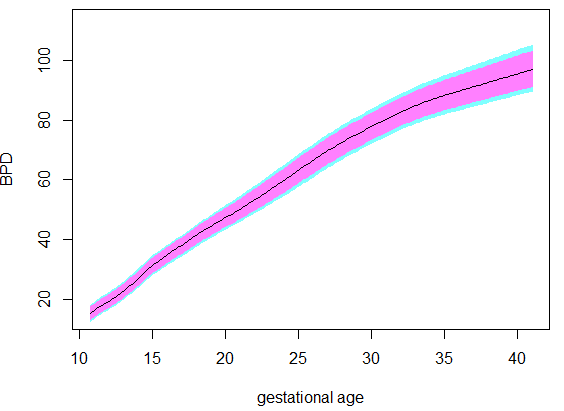


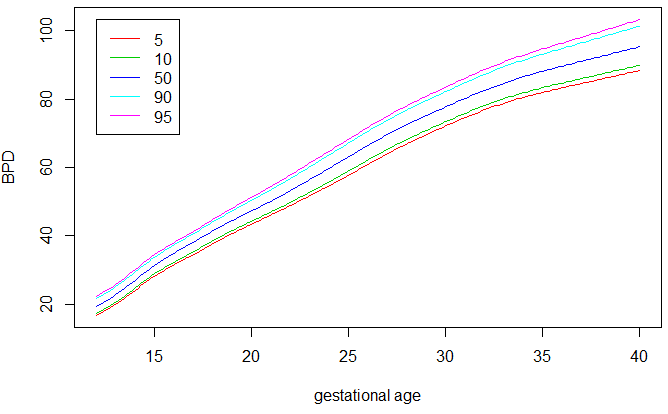


HeadCircumference (HC), *Total group*


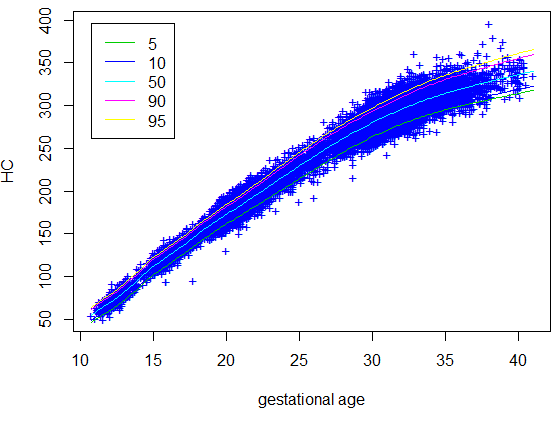


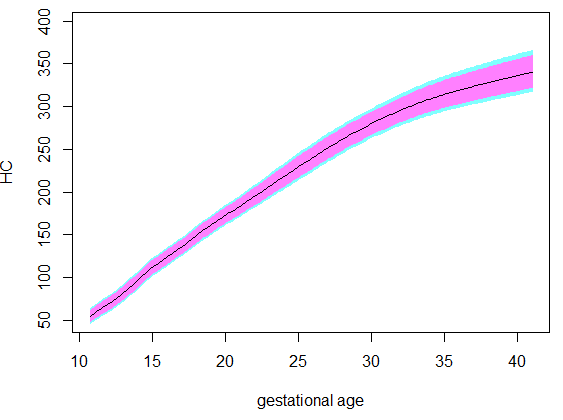


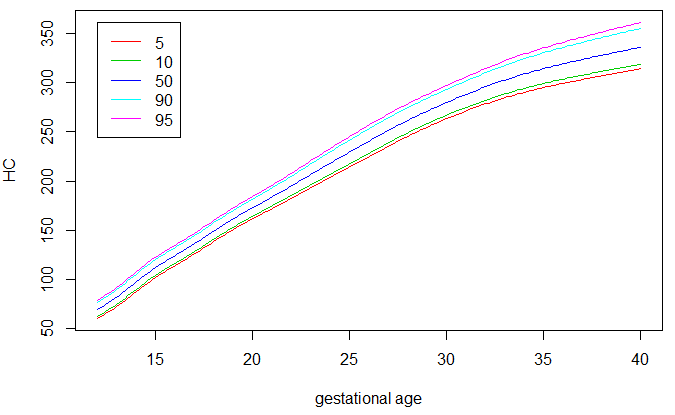


AbdominalCircumference (AC), *Total group*


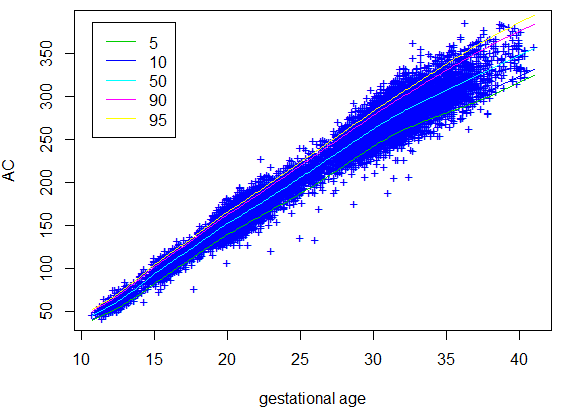


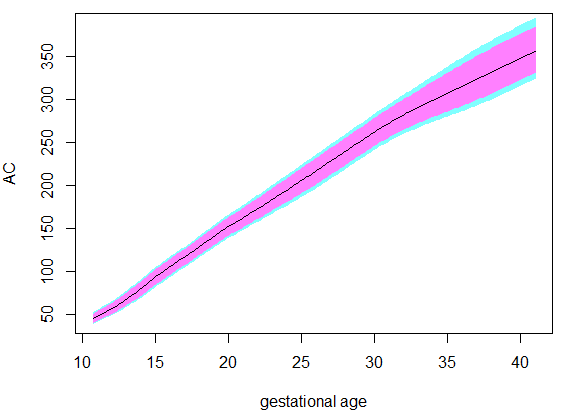


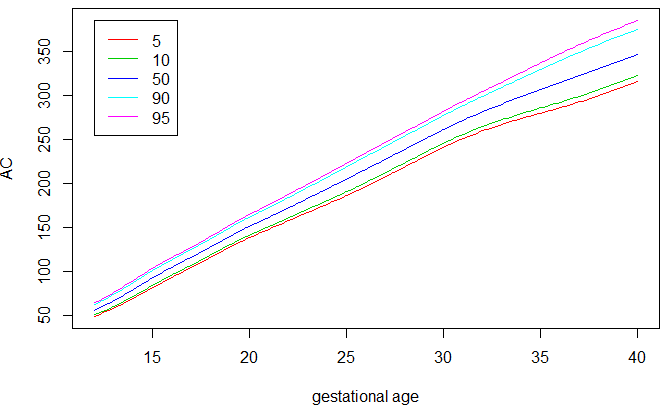


FemurLength (FL), *Total group*


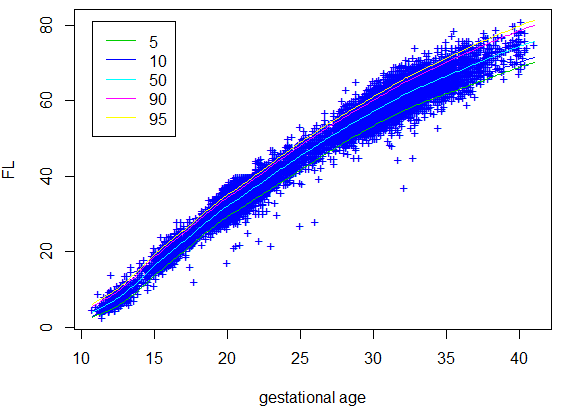


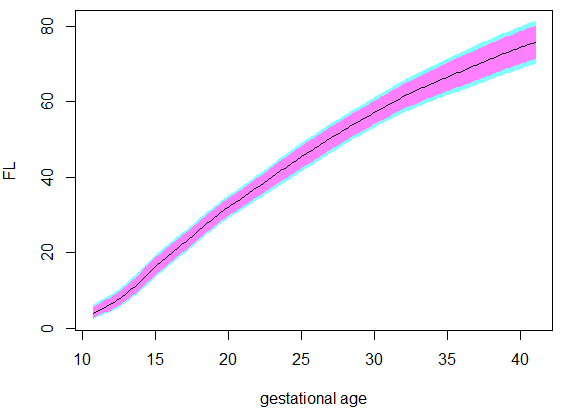


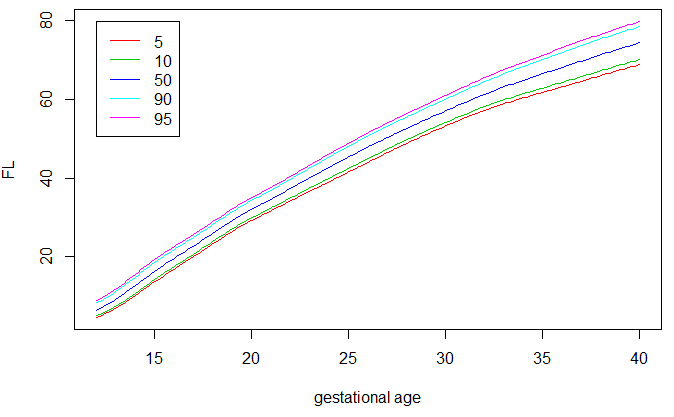


Estimated Fetal Weight (EFW) in gram, *Total group*
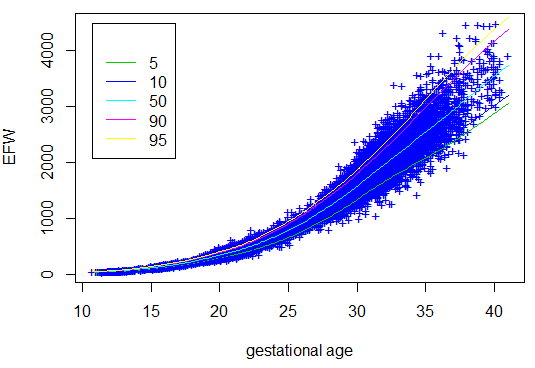

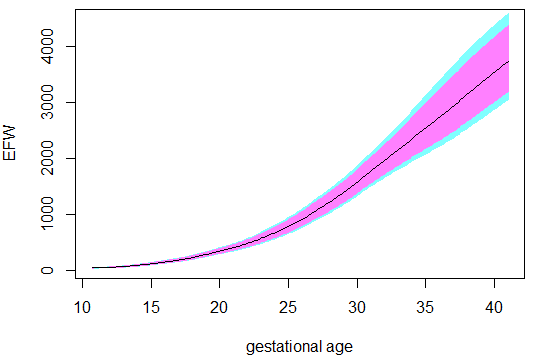

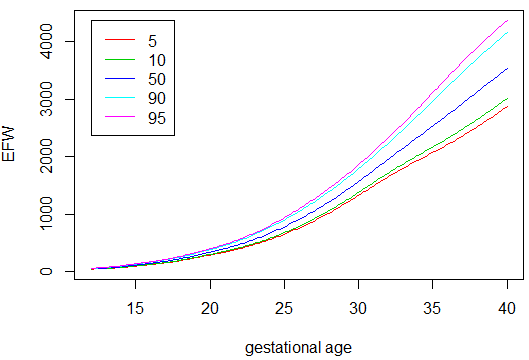


**Boys & Girls**

BiParietalDiameter (BPD), *Boys*


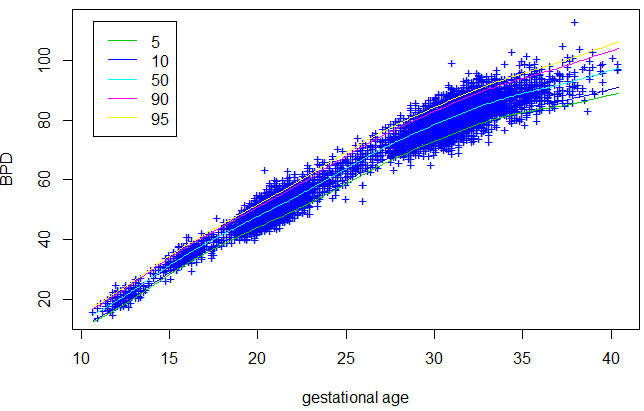


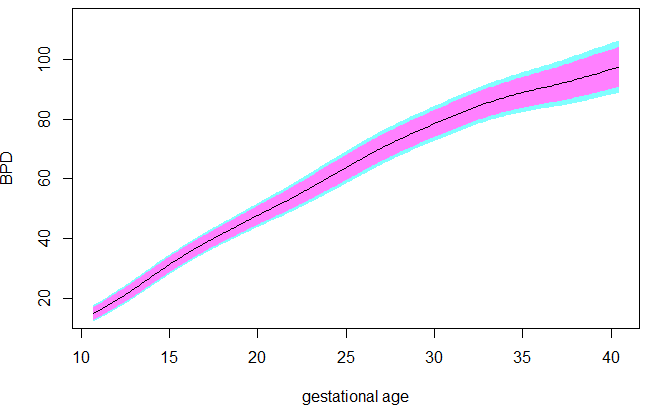


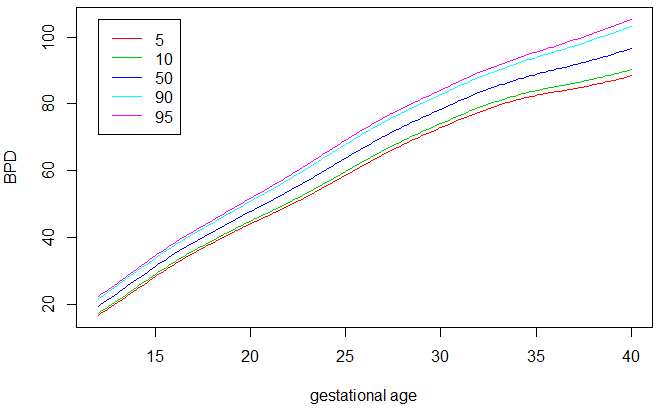


BiParietalDiameter (BPD), *Girls*


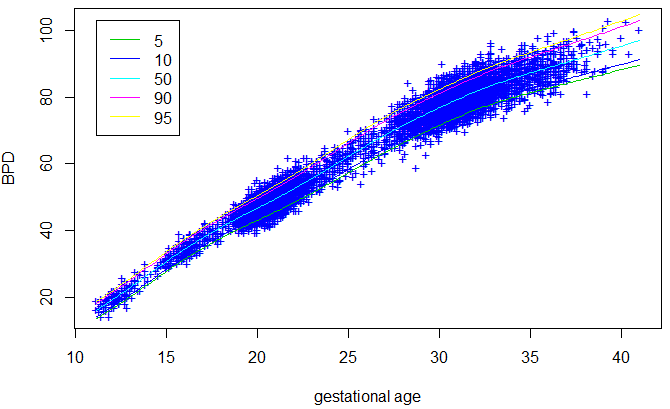


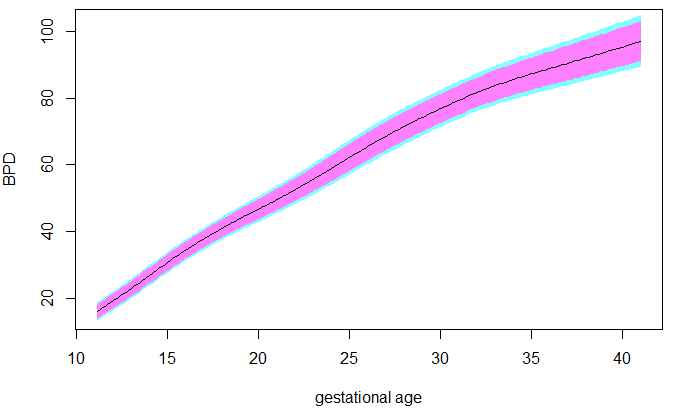


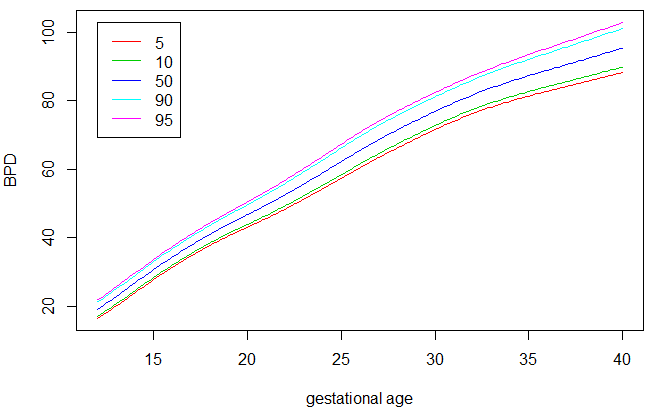


HeadCircumference (HC), *Boys*


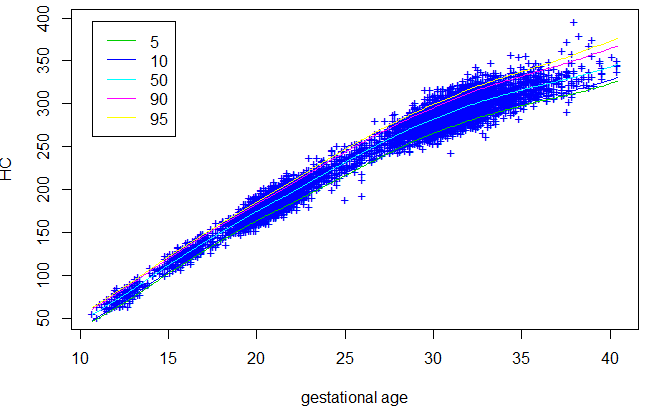


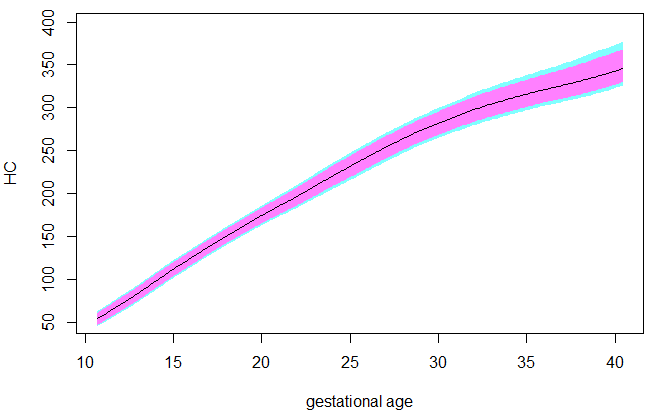


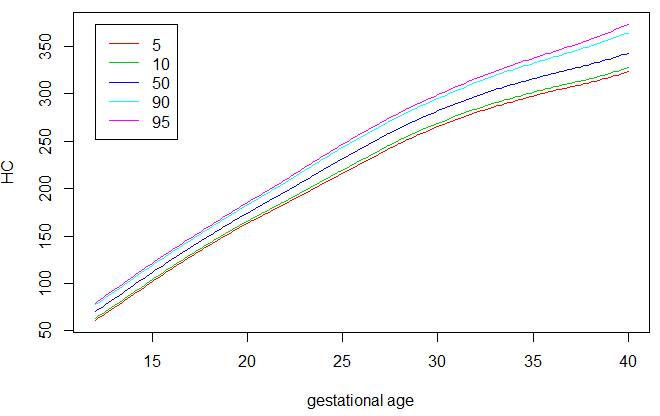


HeadCircumference (HC), *Girls*


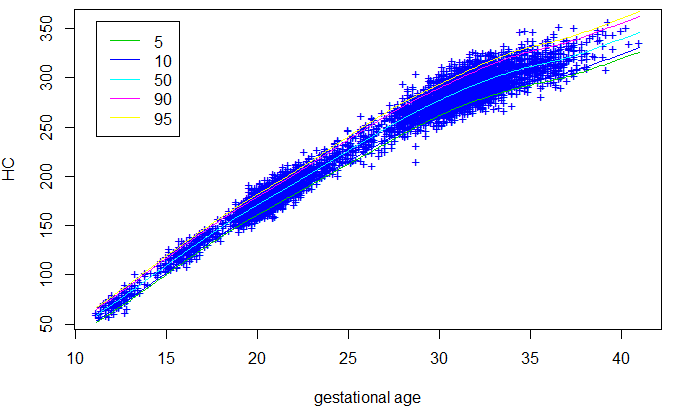


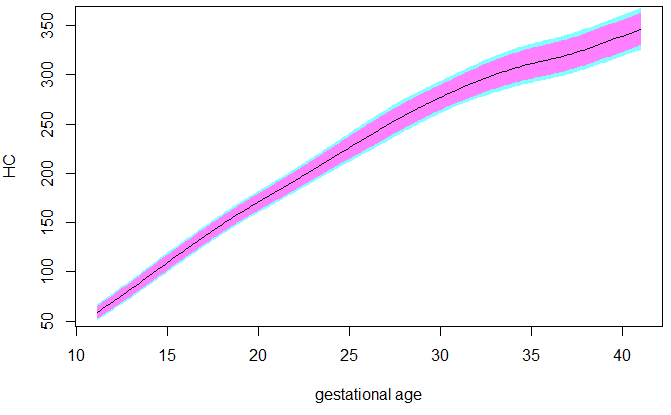


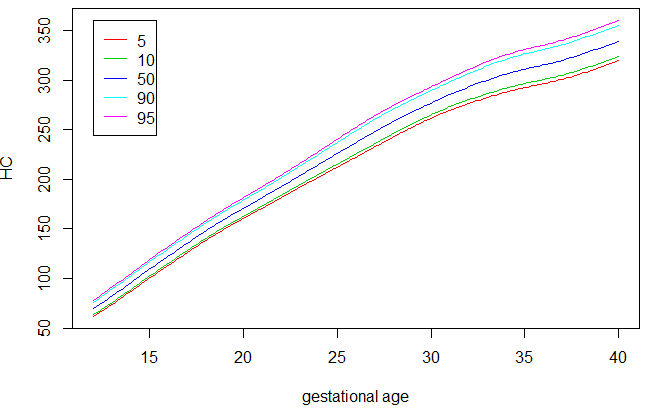


AbdominalCircumference (AC), *Boys*


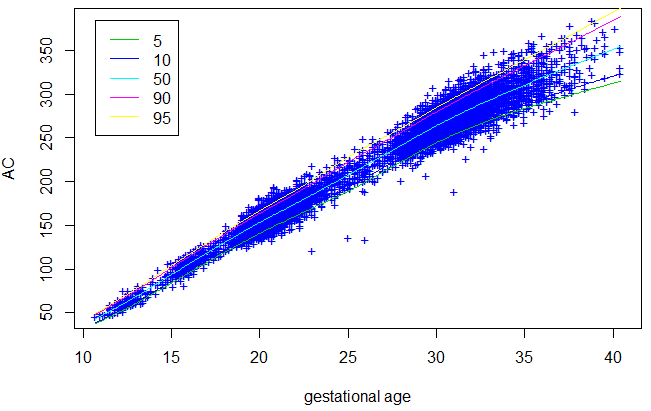


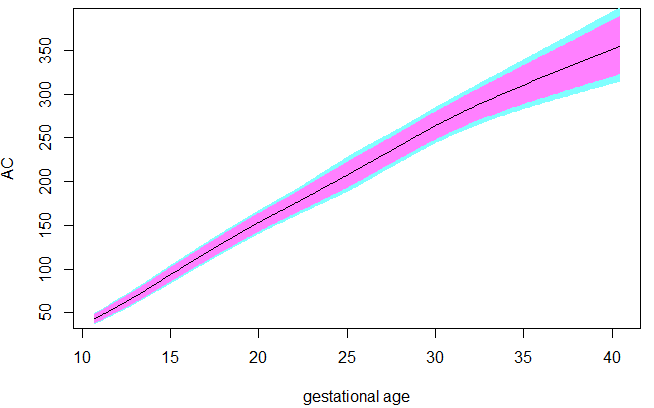


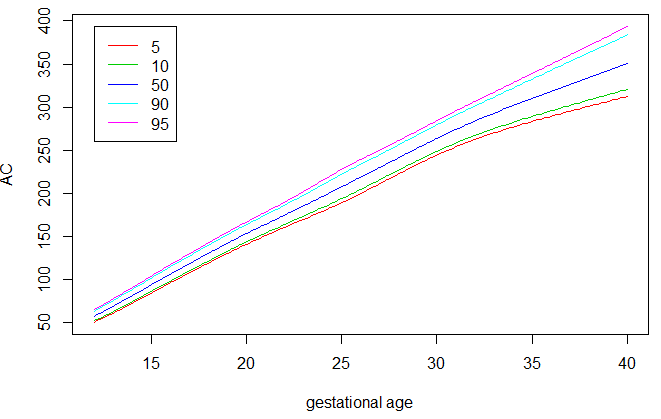


AbdominalCircumference (AC), *Girls*


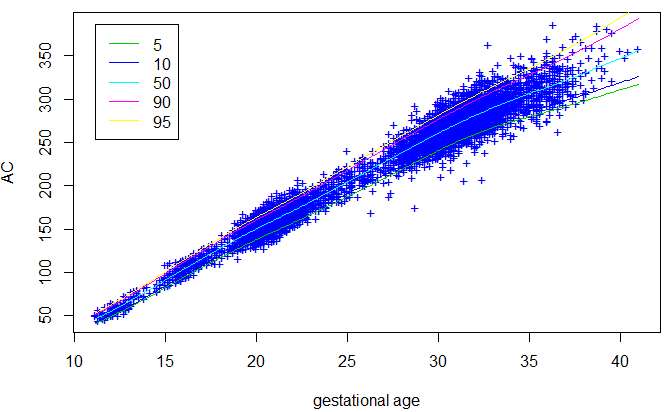


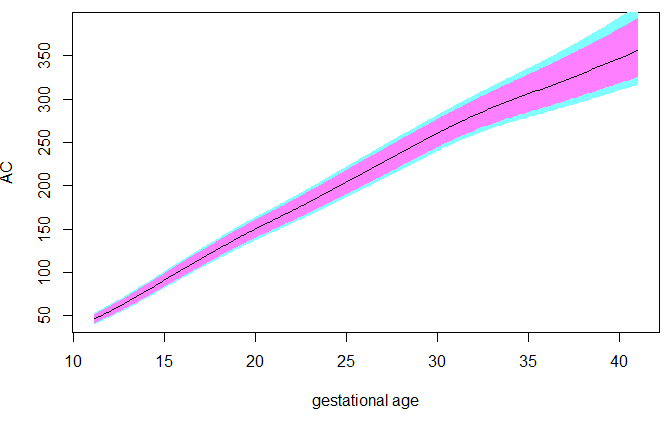


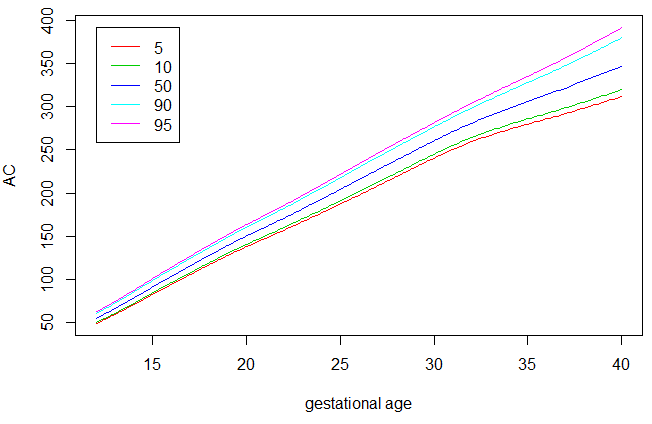


FemurLength (FL), *Boys*


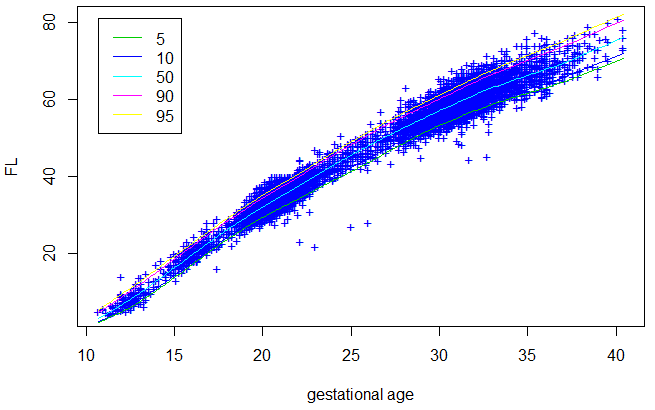


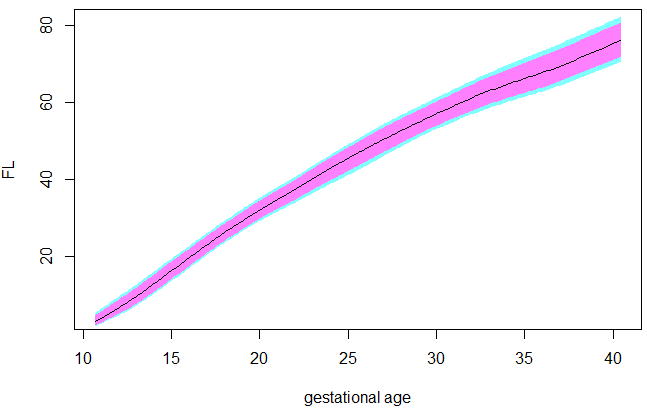


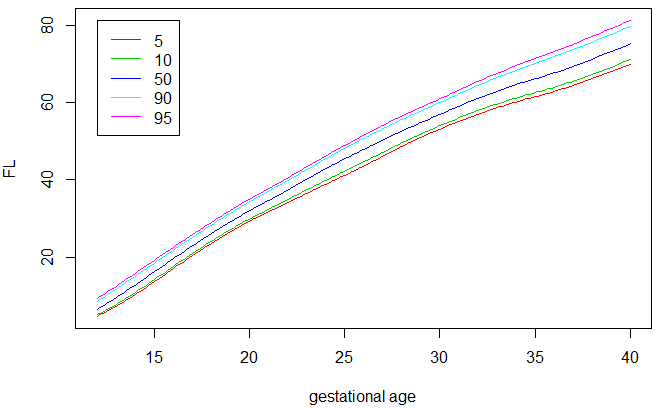


FemurLength (FL), *Girls*


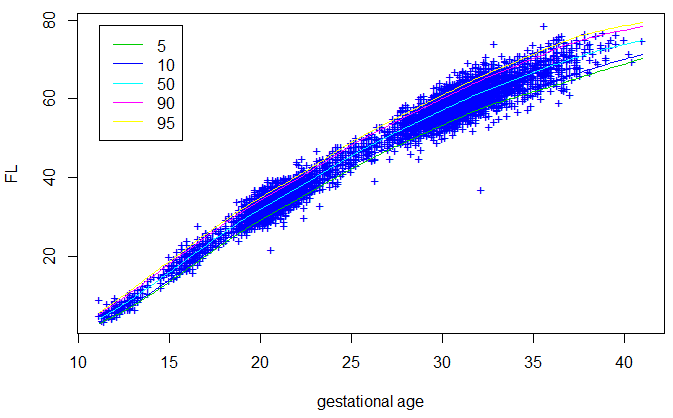


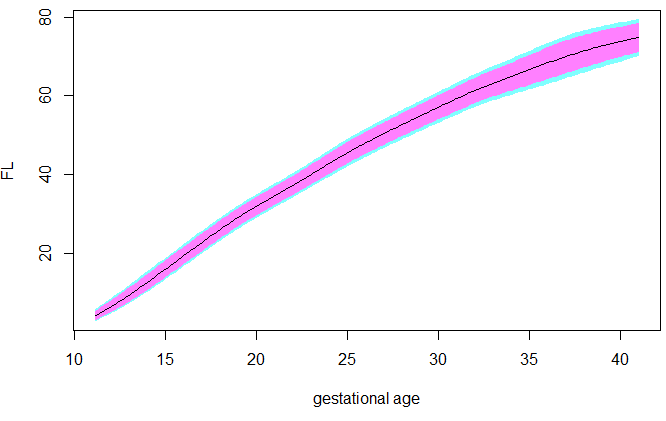


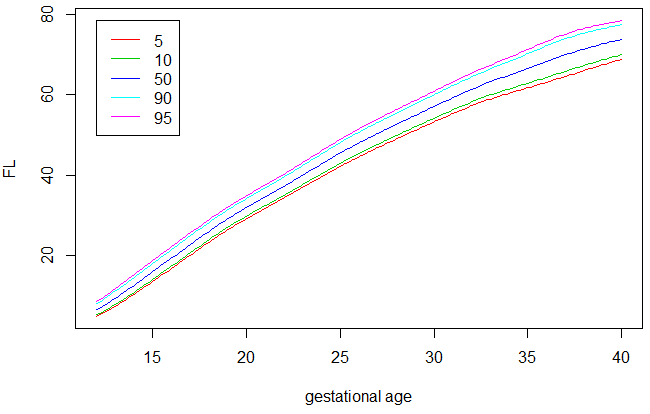


Estimated Fetal Weight (EFW), *Boys*


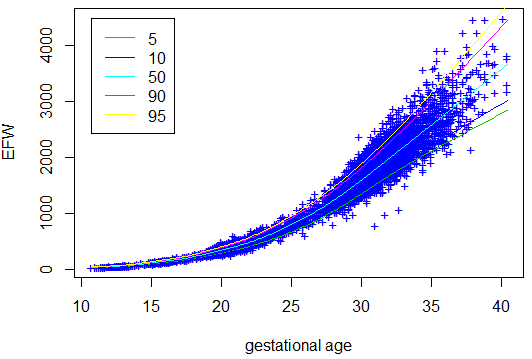

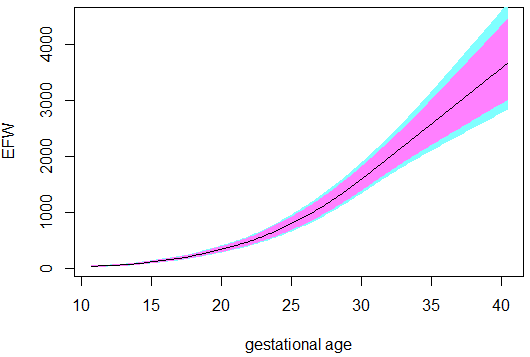

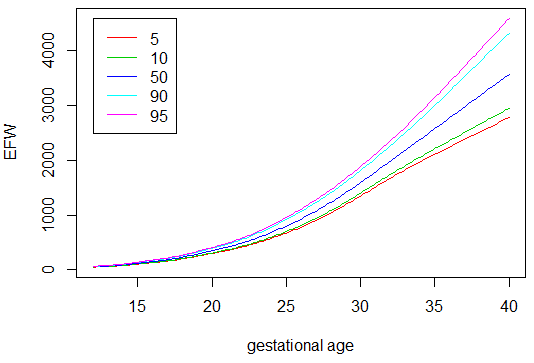


Estimated Fetal Weight (EFW), *Girls*


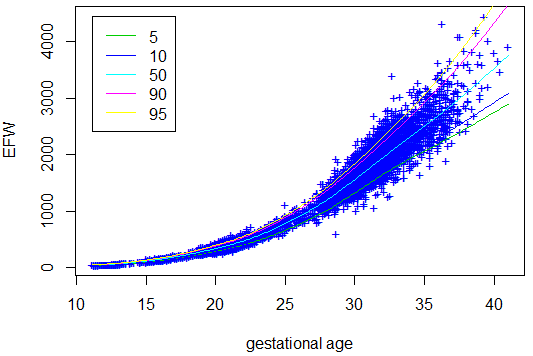

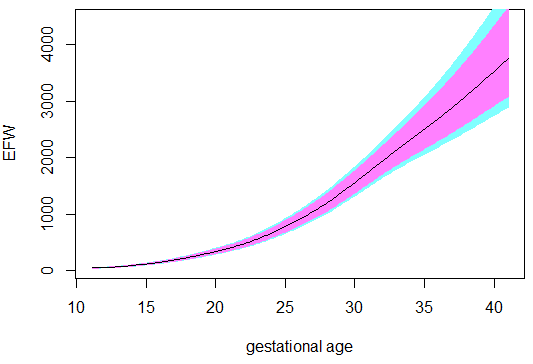

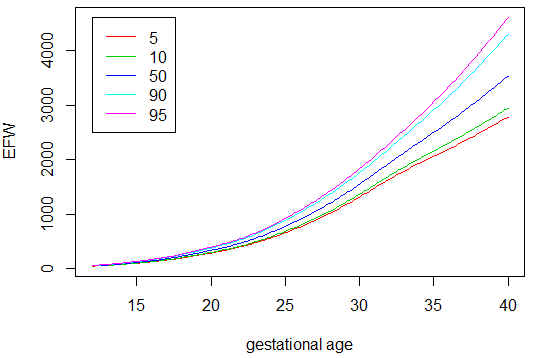

Supplement: Supplementary file 3 — Boys vs Girls combined scatterplots and curves. (DOCX 816 kb) [file 13293_2019_261_MOESM3_ESM.docx]
