# Supplementary material for: Sex differences in fetal growth and immediate birth outcomes in a low-risk Caucasian population
Source: Biol Sex Differ. 2019 Sep 9;10:48. doi: 10.1186/s13293-019-0261-7 (PMC6734449; doi:10.1186/s13293-019-0261-7)
Supplement: Supplementary file 4 — Boys vs Girls combined gridcurves and reference values. (DOCX 4051 kb) [file 13293_2019_261_MOESM4_ESM.docx]

## Additional file 4, Grid reference curves and reference tables Total, Boys & Girls

**BiParietalDiameter (BPD), *Total group***


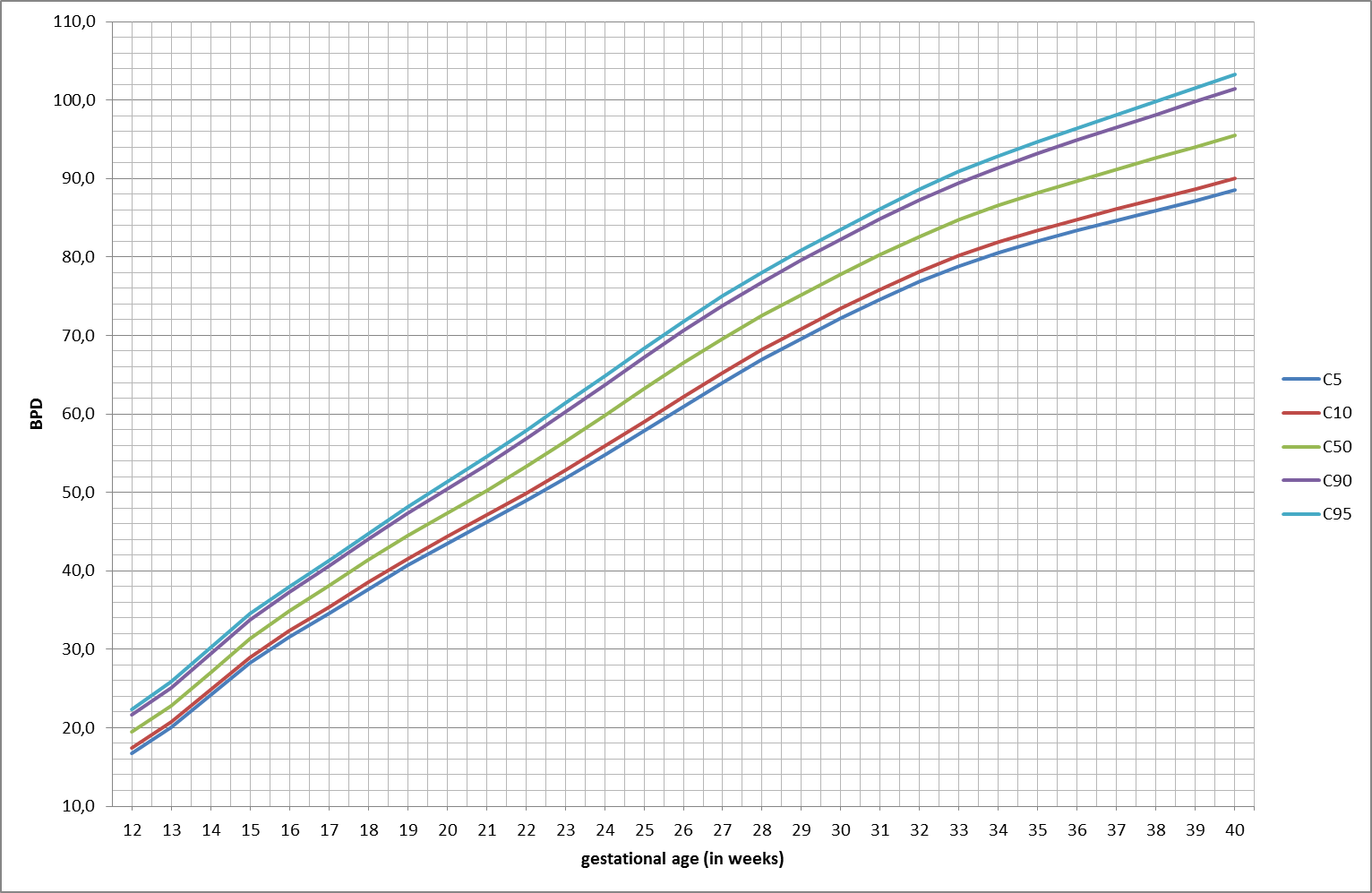


**e-Figure 1. Grid reference curve for BiParietalDiameter (BPD) in mm for total group from 12-40 weeks of gestation. Percentiles 5, 10, 50, 90 and 95.**

**HeadCircumference (HC), *Total group***


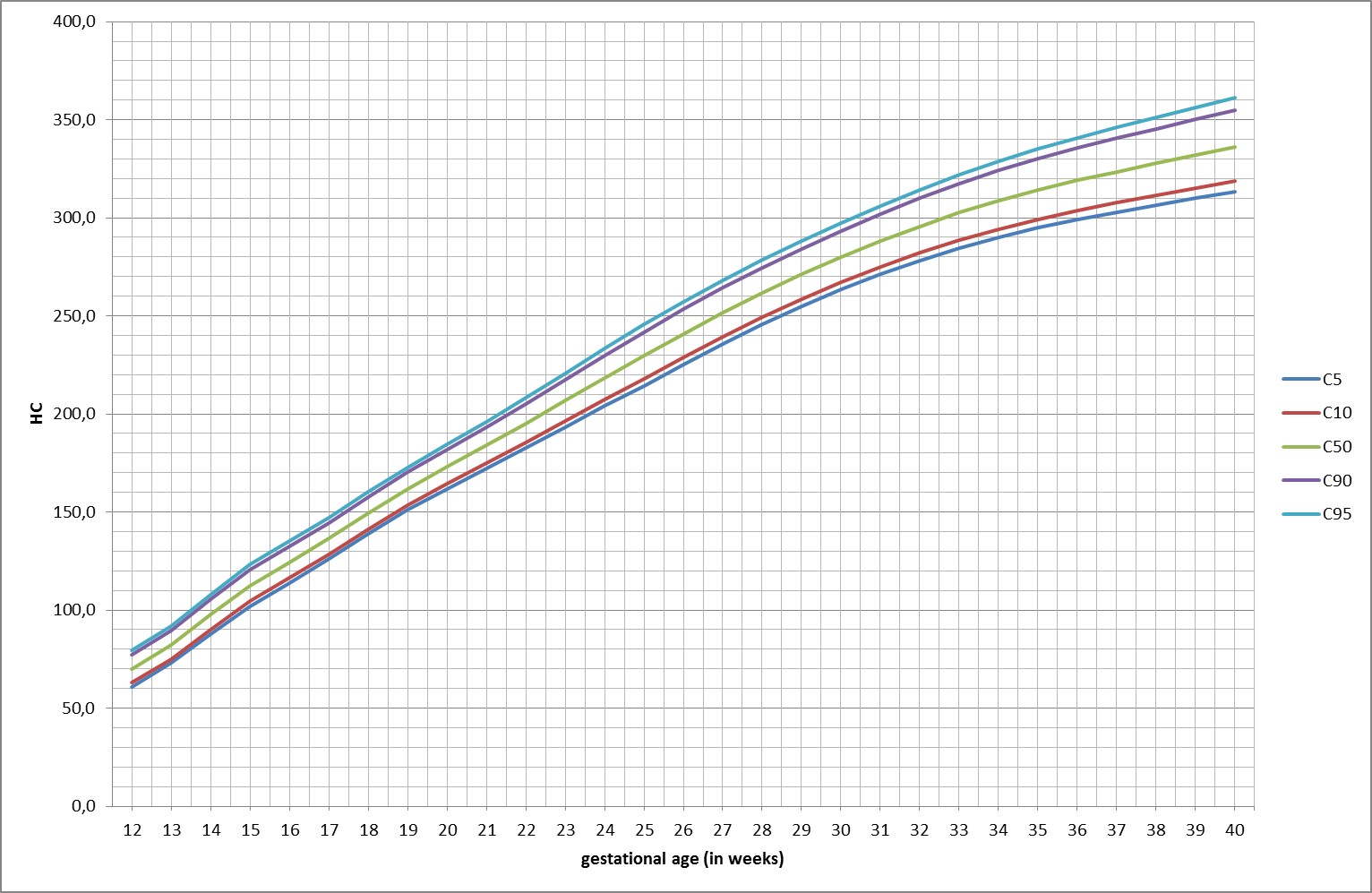


**e-Figure 2. Grid reference curve for HeadCircumference (HC) in mm for total group from 12-40 weeks of gestation. Percentiles 5, 10, 50, 90 and 95.**

**AbdominalCircumference (AC), *Total group***


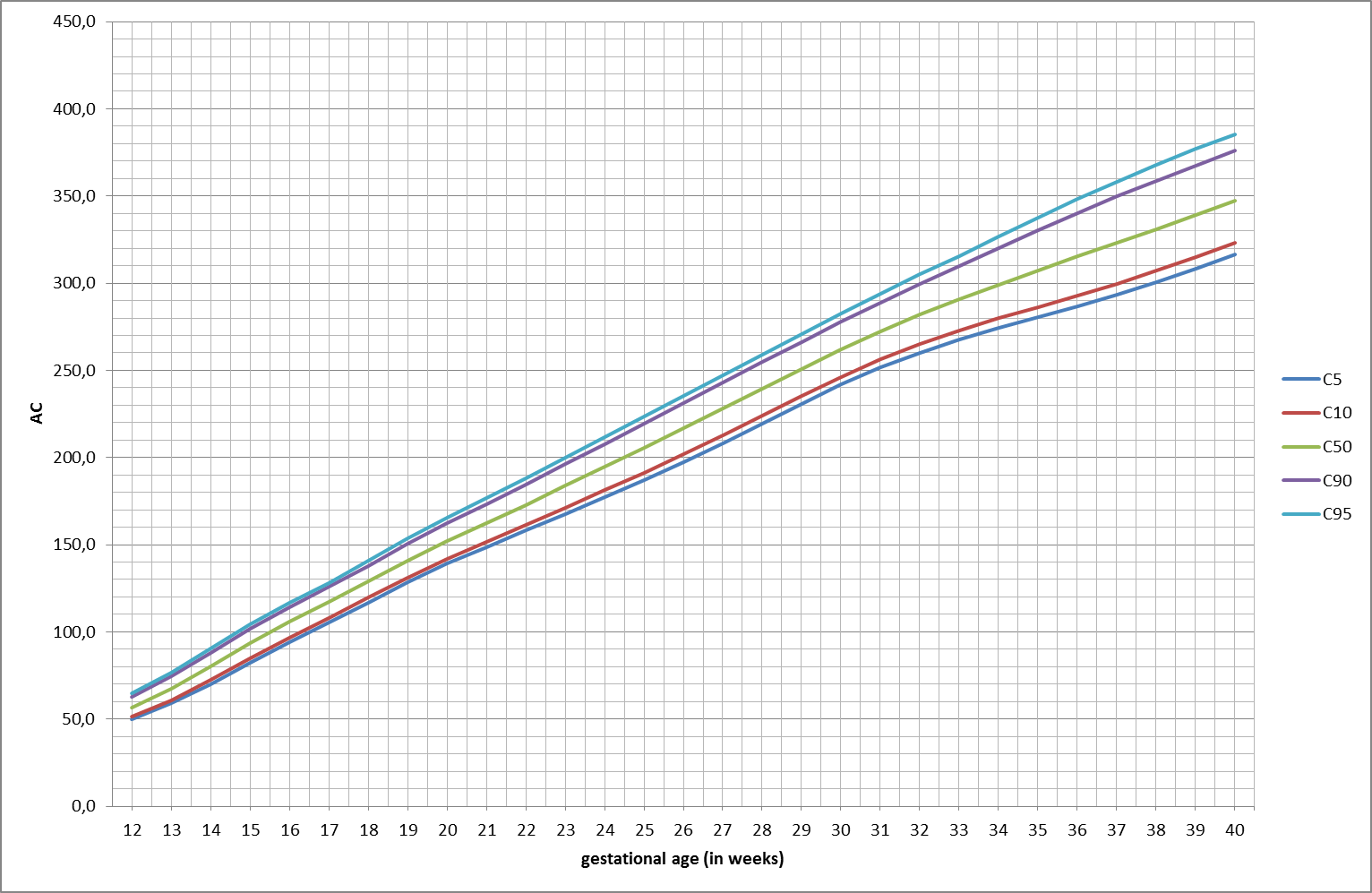


**e-Figure 3. Grid reference curve for Abdominal Circumference (AC) in mm for total group from 12-40 weeks of gestation. Percentiles 5, 10, 50, 90 and 95.**

**FemurLength (FL), *Total group***


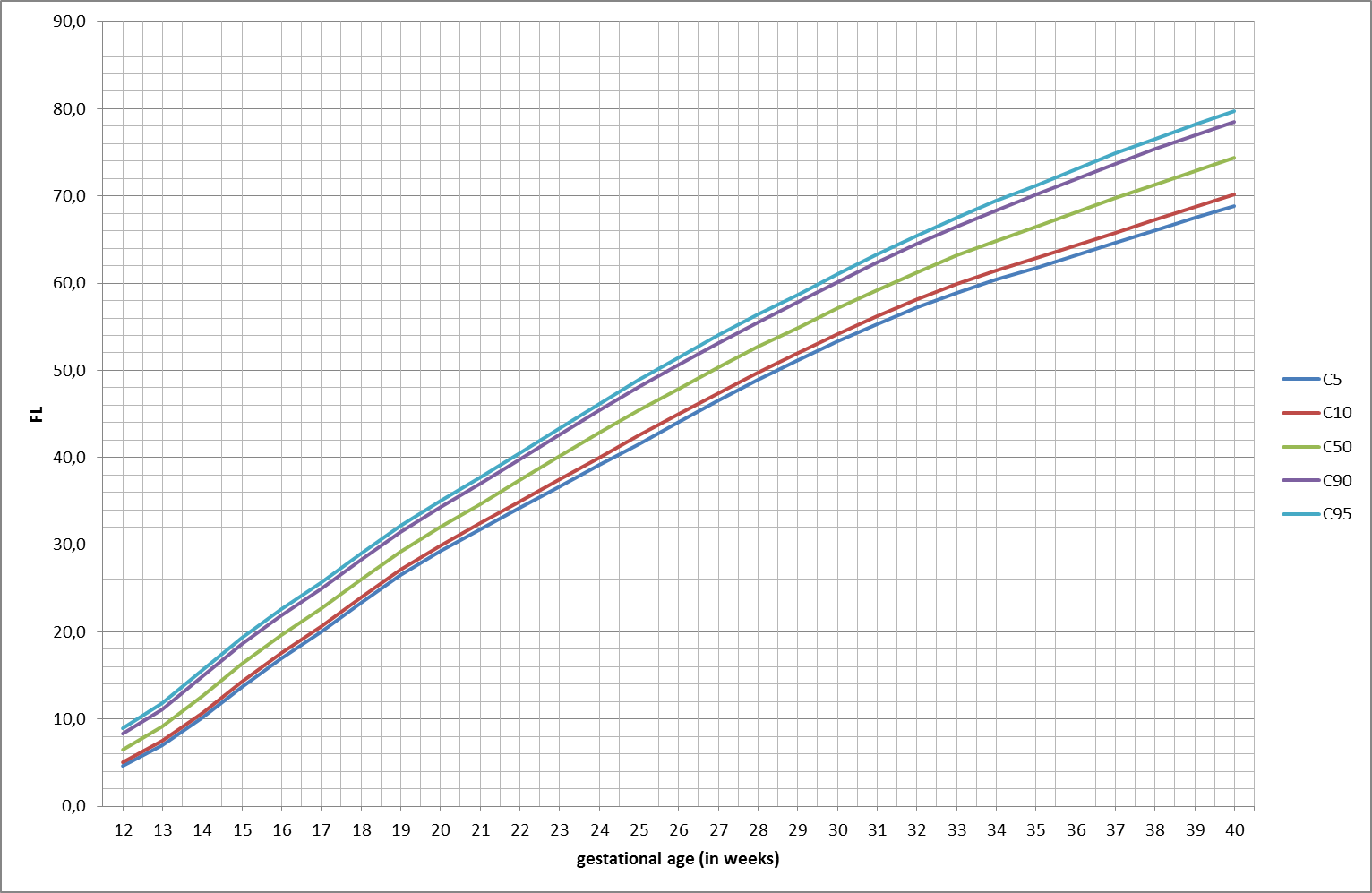


**e-Figure 4. Grid reference curve for Femur Length (FL) in mm for total group from 12-40 weeks of gestation. Percentiles 5, 10, 50, 90 and 95.**

**Estimated Fetal Weight (EFW), *Total group***

**
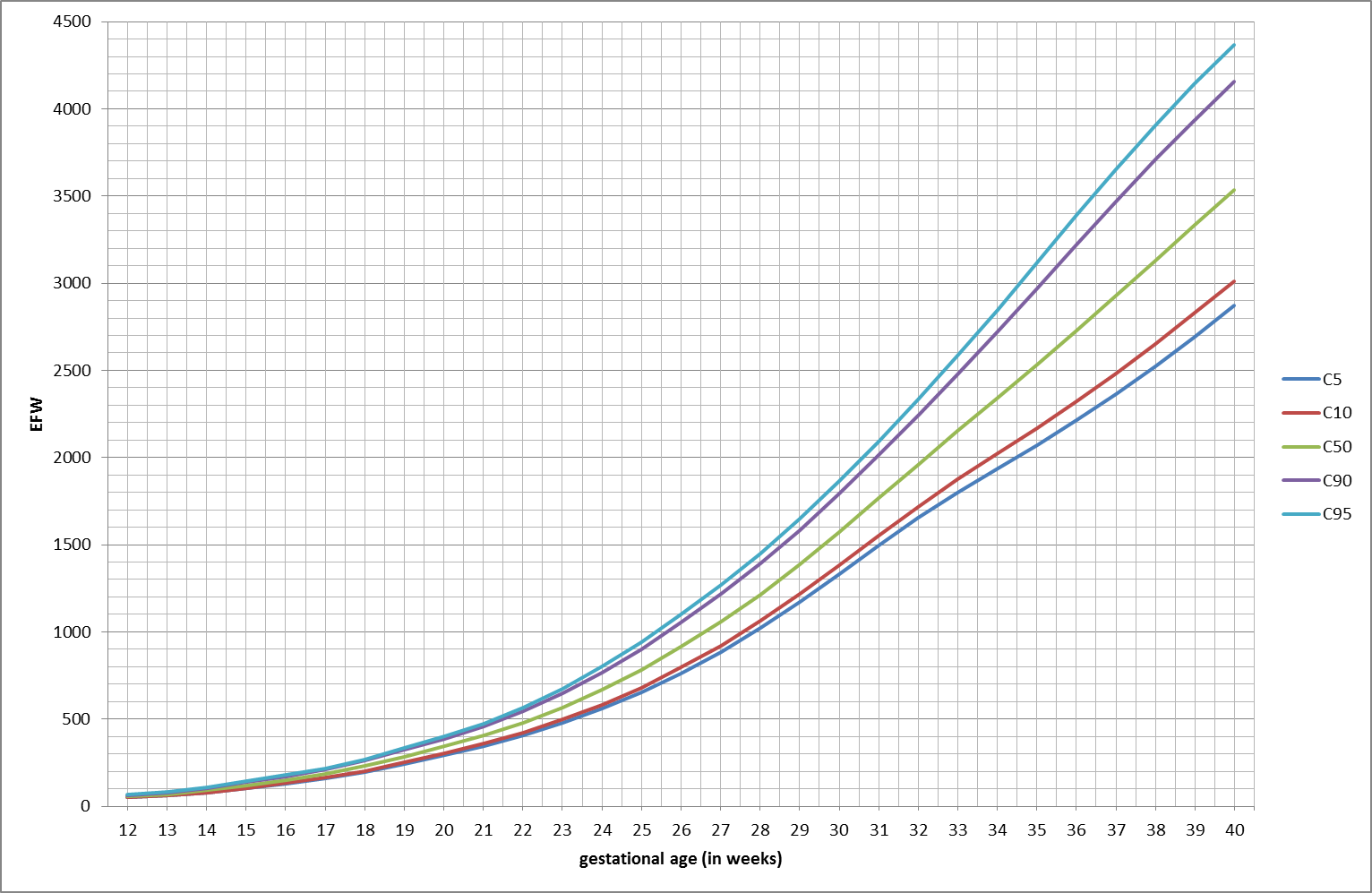
**

**e-Figure 5. Grid reference curve for Estimated Fetal Weight (EFW) in g for total group from 12-40 weeks of gestation. Percentiles 5, 10, 50, 90 and 95.**

**Reference values for BiParietalDiameter (BPD) in mm, *Total group***

GA = Gestational Age in weeks

| **GA** | **BPD** | | | | |
| --- | --- | --- | --- | --- | --- |
|  | **C5** | **C10** | **C50** | **C90** | **C95** |
| **12** | 16,8 | 17,4 | 19,5 | 21,7 | 22,4 |
| **13** | 20,1 | 20,7 | 22,8 | 25,1 | 25,8 |
| **14** | 24,2 | 24,8 | 27,1 | 29,4 | 30,2 |
| **15** | 28,2 | 28,9 | 31,4 | 33,8 | 34,5 |
| **16** | 31,5 | 32,3 | 34,9 | 37,3 | 38,0 |
| **17** | 34,5 | 35,4 | 38,1 | 40,6 | 41,3 |
| **18** | 37,6 | 38,5 | 41,4 | 44,0 | 44,7 |
| **19** | 40,7 | 41,6 | 44,5 | 47,3 | 48,1 |
| **20** | 43,5 | 44,3 | 47,3 | 50,4 | 51,3 |
| **21** | 46,2 | 47,1 | 50,2 | 53,5 | 54,5 |
| **22** | 48,9 | 49,9 | 53,3 | 56,8 | 57,9 |
| **23** | 51,8 | 52,8 | 56,5 | 60,2 | 61,3 |
| **24** | 54,7 | 55,9 | 59,8 | 63,7 | 64,8 |
| **25** | 57,8 | 59,0 | 63,2 | 67,2 | 68,3 |
| **26** | 60,9 | 62,2 | 66,5 | 70,6 | 71,8 |
| **27** | 64,0 | 65,3 | 69,6 | 73,8 | 75,0 |
| **28** | 66,9 | 68,2 | 72,5 | 76,8 | 78,1 |
| **29** | 69,7 | 70,9 | 75,2 | 79,6 | 80,9 |
| **30** | 72,2 | 73,5 | 77,8 | 82,2 | 83,6 |
| **31** | 74,7 | 75,9 | 80,3 | 84,8 | 86,2 |
| **32** | 76,9 | 78,2 | 82,7 | 87,3 | 88,7 |
| **33** | 78,9 | 80,2 | 84,8 | 89,5 | 90,9 |
| **34** | 80,6 | 81,9 | 86,6 | 91,5 | 92,9 |
| **35** | 82,1 | 83,4 | 88,2 | 93,2 | 94,7 |
| **36** | 83,4 | 84,8 | 89,7 | 94,9 | 96,5 |
| **37** | 84,7 | 86,1 | 91,2 | 96,5 | 98,2 |
| **38** | 85,9 | 87,4 | 92,6 | 98,2 | 99,9 |
| **39** | 87,2 | 88,7 | 94,1 | 99,8 | 101,6 |
| **40** | 88,5 | 90,0 | 95,5 | 101,5 | 103,3 |

e-Table 3. Reference values in mm for fetal biparietal diameter (BPD) in the total group for each gestational week for the median and 5^th^, 10^th^, 90^th^ and 95^th^ centiles. GA = gestational weeks.

**Reference values for HeadCircumference (HC) in mm, *Total group***

GA = Gestational Age in weeks

| **GA** | **HC** | | | | |
| --- | --- | --- | --- | --- | --- |
|  | **C5** | **C10** | **C50** | **C90** | **C95** |
| **12** | 60,8 | 62,8 | 69,8 | 77,0 | 79,2 |
| **13** | 72,9 | 75,0 | 82,2 | 89,6 | 91,9 |
| **14** | 87,6 | 90,0 | 97,6 | 105,4 | 108,0 |
| **15** | 101,8 | 104,4 | 112,3 | 120,5 | 123,3 |
| **16** | 113,9 | 116,5 | 124,4 | 132,5 | 135,3 |
| **17** | 126,0 | 128,5 | 136,4 | 144,5 | 147,2 |
| **18** | 138,8 | 141,2 | 149,3 | 157,5 | 160,1 |
| **19** | 150,9 | 153,4 | 161,7 | 170,2 | 172,7 |
| **20** | 161,8 | 164,3 | 172,9 | 181,8 | 184,4 |
| **21** | 172,1 | 174,7 | 183,9 | 193,2 | 196,0 |
| **22** | 182,7 | 185,4 | 195,1 | 205,1 | 208,1 |
| **23** | 193,3 | 196,4 | 206,7 | 217,4 | 220,7 |
| **24** | 203,9 | 207,2 | 218,3 | 229,7 | 233,3 |
| **25** | 214,4 | 218,0 | 229,6 | 241,7 | 245,6 |
| **26** | 224,9 | 228,6 | 240,7 | 253,2 | 257,3 |
| **27** | 235,5 | 239,2 | 251,5 | 264,3 | 268,3 |
| **28** | 245,7 | 249,3 | 261,7 | 274,6 | 278,6 |
| **29** | 254,9 | 258,5 | 271,1 | 284,2 | 288,1 |
| **30** | 263,4 | 267,0 | 279,8 | 293,1 | 297,2 |
| **31** | 271,2 | 274,9 | 288,0 | 301,8 | 305,9 |
| **32** | 278,3 | 282,1 | 295,7 | 309,9 | 314,2 |
| **33** | 284,6 | 288,6 | 302,6 | 317,4 | 321,9 |
| **34** | 290,2 | 294,3 | 308,8 | 324,2 | 328,9 |
| **35** | 295,0 | 299,3 | 314,3 | 330,2 | 335,1 |
| **36** | 299,2 | 303,6 | 319,1 | 335,5 | 340,7 |
| **37** | 302,9 | 307,6 | 323,5 | 340,5 | 346,0 |
| **38** | 306,5 | 311,4 | 327,8 | 345,4 | 351,2 |
| **39** | 309,9 | 315,1 | 331,9 | 350,1 | 356,3 |
| **40** | 313,2 | 318,6 | 336,0 | 354,8 | 361,3 |

e-Table 4. Reference values in mm for fetal head-circumference (HC) in the total group for each gestational week for the median and 5^th^, 10^th^, 90^th^ and 95^th^ centiles. GA = gestational weeks.

**Reference values for AbdominalCircumference (AC) in mm, *Total group***

GA = Gestational Age in weeks

| **GA** | **AC** | | | | |
| --- | --- | --- | --- | --- | --- |
|  | **C5** | **C10** | **C50** | **C90** | **C95** |
| **12** | 49,7 | 51,2 | 56,6 | 62,8 | 64,8 |
| **13** | 58,9 | 60,8 | 67,4 | 74,5 | 76,7 |
| **14** | 70,0 | 72,3 | 80,1 | 88,0 | 90,4 |
| **15** | 82,2 | 84,8 | 93,4 | 101,7 | 104,2 |
| **16** | 94,0 | 96,7 | 105,6 | 114,1 | 116,6 |
| **17** | 105,2 | 107,9 | 116,9 | 125,6 | 128,2 |
| **18** | 116,8 | 119,5 | 128,8 | 137,9 | 140,6 |
| **19** | 128,4 | 131,2 | 140,7 | 150,5 | 153,4 |
| **20** | 139,1 | 141,9 | 151,9 | 162,3 | 165,4 |
| **21** | 148,6 | 151,7 | 162,2 | 173,2 | 176,6 |
| **22** | 158,1 | 161,4 | 172,8 | 184,5 | 188,0 |
| **23** | 167,7 | 171,3 | 183,7 | 196,1 | 199,9 |
| **24** | 177,2 | 181,2 | 194,6 | 207,8 | 211,7 |
| **25** | 186,9 | 191,3 | 205,6 | 219,4 | 223,5 |
| **26** | 197,1 | 201,8 | 216,8 | 231,2 | 235,3 |
| **27** | 208,1 | 212,8 | 228,1 | 242,9 | 247,2 |
| **28** | 219,5 | 224,1 | 239,5 | 254,6 | 259,1 |
| **29** | 230,9 | 235,4 | 250,8 | 266,3 | 270,9 |
| **30** | 241,7 | 246,3 | 261,9 | 277,8 | 282,7 |
| **31** | 251,7 | 256,3 | 272,4 | 289,0 | 294,1 |
| **32** | 260,2 | 265,1 | 281,9 | 299,5 | 305,0 |
| **33** | 267,6 | 272,8 | 290,7 | 309,7 | 315,7 |
| **34** | 274,3 | 279,8 | 299,2 | 320,1 | 326,6 |
| **35** | 280,5 | 286,4 | 307,3 | 330,3 | 337,6 |
| **36** | 286,6 | 292,9 | 315,2 | 340,1 | 348,2 |
| **37** | 293,2 | 299,7 | 323,1 | 349,6 | 358,3 |
| **38** | 300,5 | 307,1 | 331,1 | 358,8 | 367,9 |
| **39** | 308,3 | 314,9 | 339,2 | 367,5 | 377,0 |
| **40** | 316,4 | 323,0 | 347,3 | 375,9 | 385,6 |

e-Table 5. Reference values in mm for fetal abdominal circumference (AC) in the total group for each gestational week for the median and 5^th^, 10^th^, 90^th^ and 95^th^ centiles. GA = gestational weeks.

**Reference values for FemurLength (FL) in mm, *Total group***

GA = Gestational Age in weeks

| **GA** | **FL** | | | | |
| --- | --- | --- | --- | --- | --- |
|  | **C5** | **C10** | **C50** | **C90** | **C95** |
| **12** | 4,7 | 5,1 | 6,5 | 8,3 | 9,0 |
| **13** | 7,0 | 7,5 | 9,2 | 11,1 | 11,8 |
| **14** | 10,1 | 10,7 | 12,7 | 14,9 | 15,6 |
| **15** | 13,6 | 14,2 | 16,3 | 18,6 | 19,3 |
| **16** | 16,9 | 17,5 | 19,6 | 21,9 | 22,6 |
| **17** | 20,1 | 20,7 | 22,7 | 25,0 | 25,7 |
| **18** | 23,3 | 23,9 | 26,0 | 28,2 | 28,9 |
| **19** | 26,5 | 27,1 | 29,2 | 31,4 | 32,2 |
| **20** | 29,3 | 29,9 | 32,1 | 34,3 | 35,1 |
| **21** | 31,7 | 32,4 | 34,7 | 37,0 | 37,7 |
| **22** | 34,2 | 35,0 | 37,4 | 39,7 | 40,5 |
| **23** | 36,7 | 37,5 | 40,1 | 42,6 | 43,4 |
| **24** | 39,1 | 40,0 | 42,8 | 45,4 | 46,2 |
| **25** | 41,6 | 42,5 | 45,4 | 48,1 | 48,9 |
| **26** | 44,0 | 45,0 | 47,9 | 50,7 | 51,5 |
| **27** | 46,5 | 47,4 | 50,3 | 53,2 | 54,0 |
| **28** | 48,9 | 49,8 | 52,7 | 55,5 | 56,4 |
| **29** | 51,1 | 52,0 | 54,9 | 57,8 | 58,7 |
| **30** | 53,3 | 54,2 | 57,1 | 60,1 | 61,0 |
| **31** | 55,3 | 56,2 | 59,2 | 62,3 | 63,3 |
| **32** | 57,2 | 58,1 | 61,3 | 64,5 | 65,5 |
| **33** | 58,9 | 59,9 | 63,2 | 66,5 | 67,5 |
| **34** | 60,4 | 61,4 | 64,9 | 68,4 | 69,4 |
| **35** | 61,8 | 62,9 | 66,5 | 70,1 | 71,2 |
| **36** | 63,2 | 64,3 | 68,1 | 71,9 | 73,1 |
| **37** | 64,6 | 65,8 | 69,7 | 73,7 | 74,9 |
| **38** | 66,1 | 67,3 | 71,3 | 75,4 | 76,6 |
| **39** | 67,5 | 68,8 | 72,9 | 77,0 | 78,2 |
| **40** | 68,8 | 70,1 | 74,4 | 78,5 | 79,8 |

e-Table 6. Reference values in mm for fetal femur length (FL) in the total group for each gestational week for the median and 5^th^, 10^th^, 90^th^ and 95^th^ centiles. GA = gestational weeks.

**Reference values for Estimated Fetal Weight (EFW) in gram, *Total group***

GA = Gestational Age in weeks

| **GA** | **EFW** | | | | |
| --- | --- | --- | --- | --- | --- |
|  | **C5** | **C10** | **C50** | **C90** | **C95** |
| **12** | 49 | 51 | 56 | 62 | 64 |
| **13** | 59 | 61 | 69 | 78 | 81 |
| **14** | 76 | 79 | 90 | 103 | 107 |
| **15** | 101 | 104 | 119 | 137 | 143 |
| **16** | 127 | 132 | 150 | 172 | 178 |
| **17** | 157 | 163 | 184 | 209 | 217 |
| **18** | 196 | 203 | 229 | 259 | 269 |
| **19** | 244 | 252 | 284 | 321 | 333 |
| **20** | 294 | 304 | 343 | 387 | 401 |
| **21** | 345 | 358 | 404 | 458 | 475 |
| **22** | 407 | 422 | 479 | 546 | 567 |
| **23** | 478 | 497 | 567 | 649 | 676 |
| **24** | 559 | 581 | 666 | 767 | 799 |
| **25** | 653 | 680 | 782 | 901 | 940 |
| **26** | 763 | 794 | 912 | 1052 | 1097 |
| **27** | 886 | 922 | 1057 | 1216 | 1268 |
| **28** | 1022 | 1062 | 1215 | 1393 | 1451 |
| **29** | 1170 | 1215 | 1386 | 1584 | 1648 |
| **30** | 1331 | 1381 | 1571 | 1792 | 1863 |
| **31** | 1495 | 1552 | 1765 | 2012 | 2092 |
| **32** | 1653 | 1717 | 1959 | 2240 | 2332 |
| **33** | 1800 | 1875 | 2152 | 2476 | 2583 |
| **34** | 1938 | 2024 | 2343 | 2720 | 2846 |
| **35** | 2073 | 2170 | 2535 | 2971 | 3118 |
| **36** | 2212 | 2321 | 2730 | 3222 | 3389 |
| **37** | 2361 | 2480 | 2928 | 3469 | 3653 |
| **38** | 2522 | 2650 | 3130 | 3708 | 3906 |
| **39** | 2694 | 2829 | 3332 | 3937 | 4144 |
| **40** | 2871 | 3012 | 3533 | 4156 | 4369 |

e-Table 7. Reference values in g for Estimated Fetal Weight (EFW) in the total group for each gestational week for the median and 5^th^, 10^th^, 90^th^ and 95^th^ centiles. GA = gestational weeks.

**Comparison Boys & Girls**

**BiParietalDiameter (BPD), *Boys & Girls***


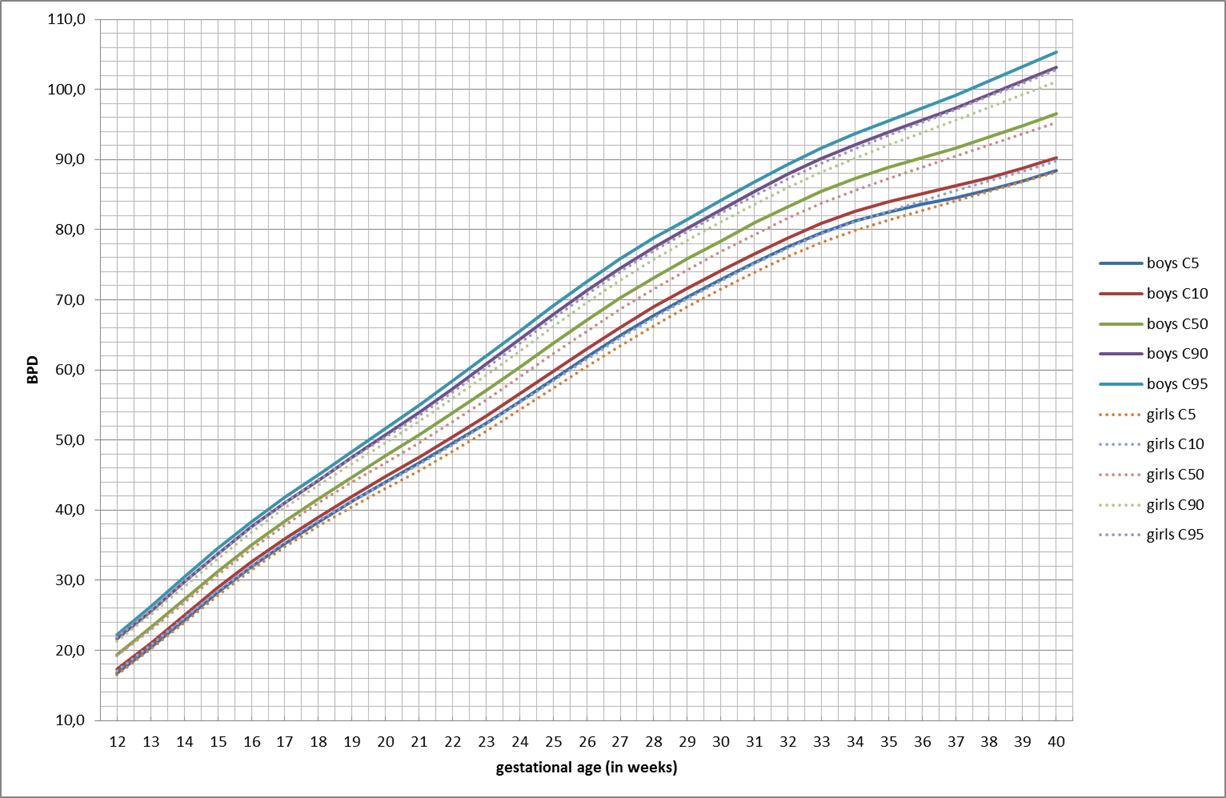


**e-Figure 6.** Grid reference curves for BiParietal Diameter (BPD) in mm for Boys and Girls from 12-40 weeks of gestation. Percentiles 5, 10, 50, 90 and 95.


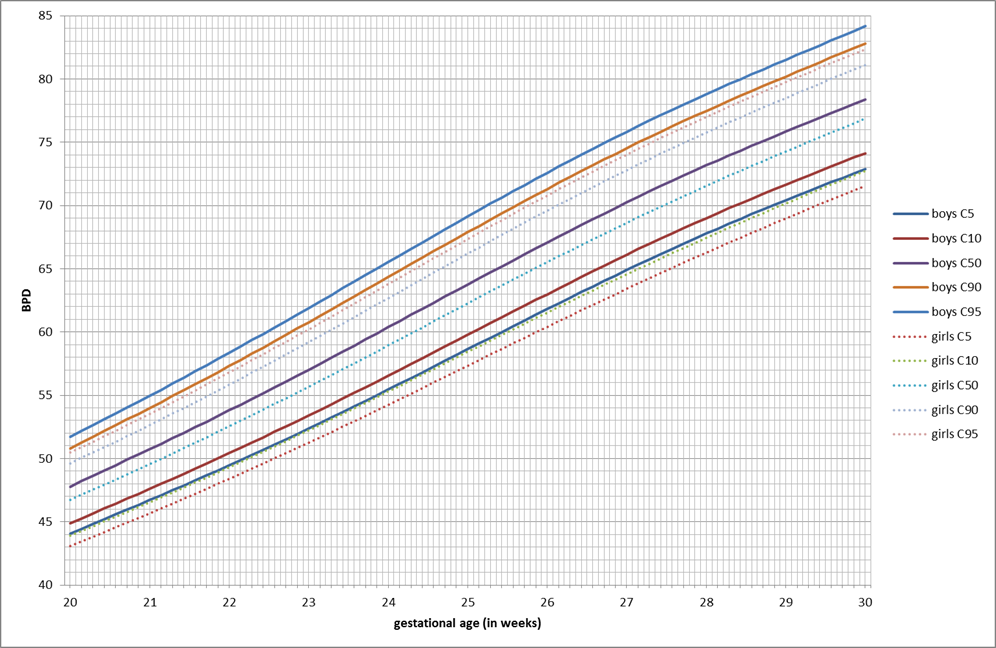


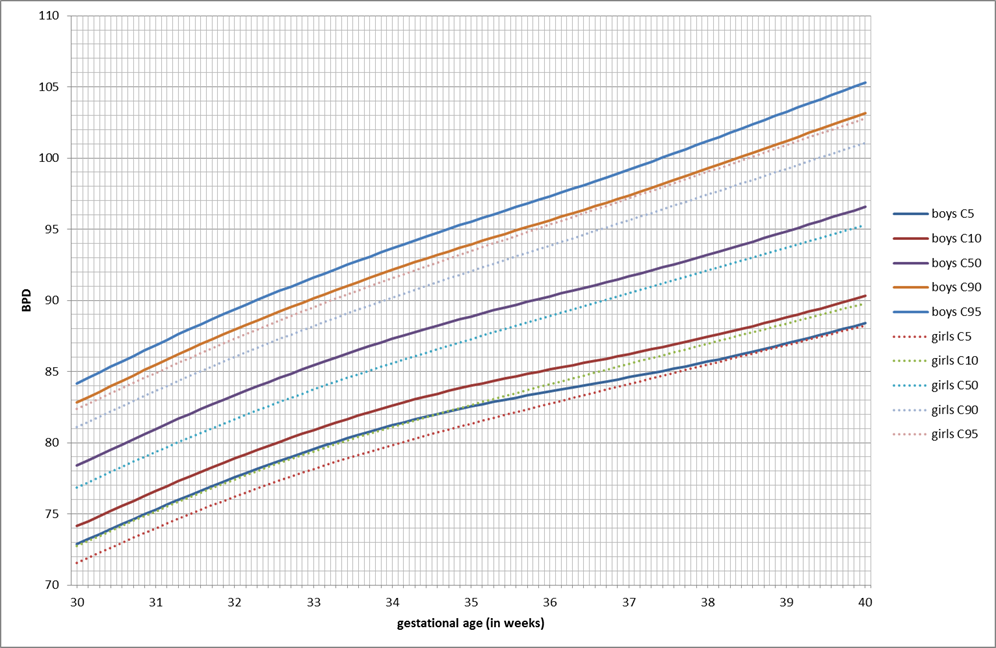


**e-Figure 7.a,b. Grid reference curves for BiParietal Diameter (BPD) in mm for Boys and Girls: a) from 20-30 weeks of gestation (upper); b) from 30-40 weeks of gestation (down). Percentiles 5, 10, 50, 90 and 95.**

**HeadCircumference (HC), *Boys & Girls***


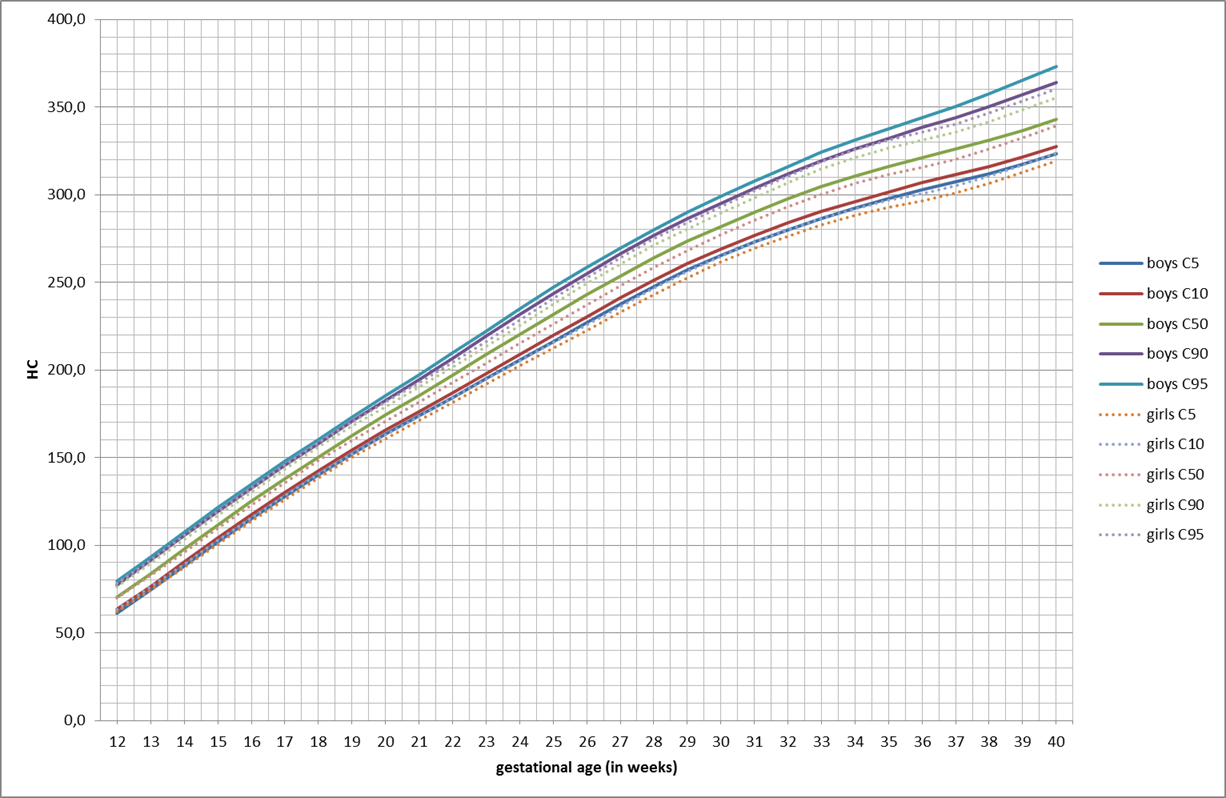


**e-Figure 3.8. Grid reference curves for Head Circumference (HC) in mm for Boys and Girls from 12-40 weeks of gestation. Percentiles 5, 10, 50, 90 and 95.**


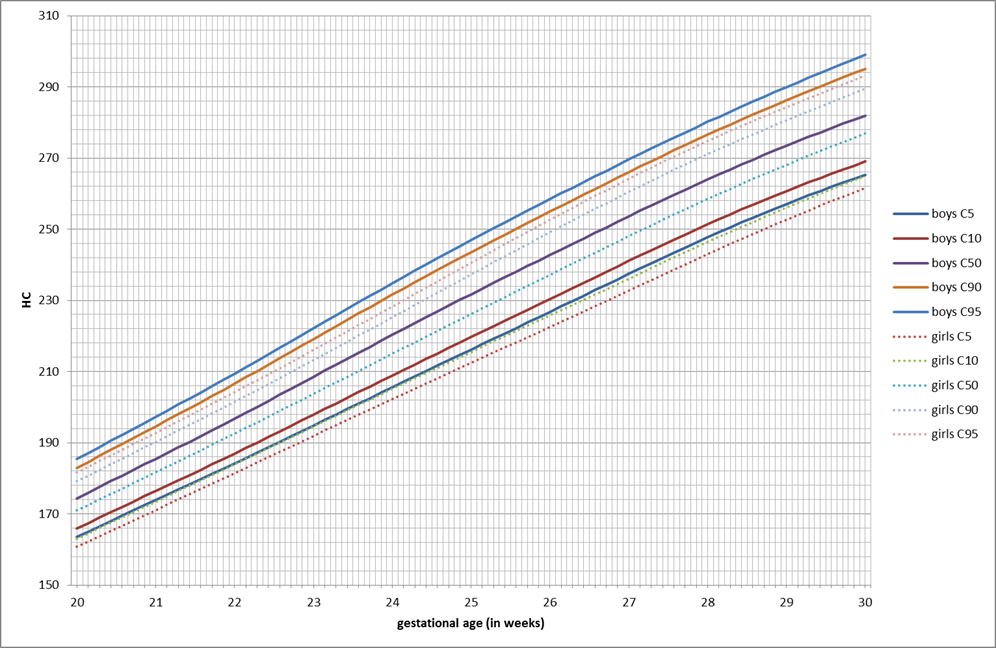


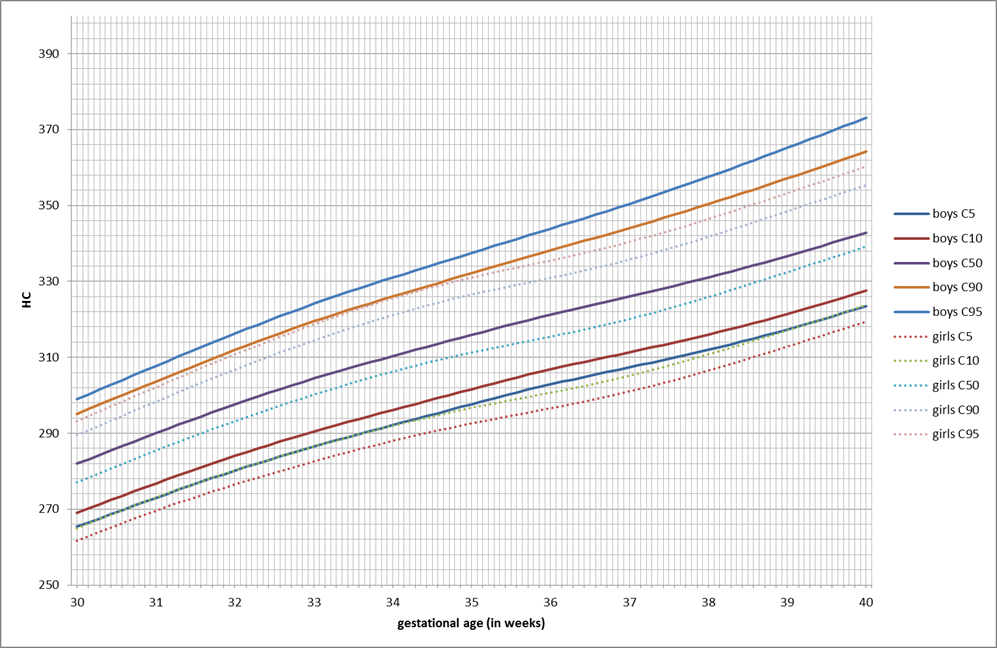


**e-Figure 9.a,b. Grid reference curves for Head Circumference (HC) in mm for Boys and Girls: a) from 20-30 weeks of gestation (upper); b) from 30-40 weeks of gestation (down). Percentiles 5, 10, 50, 90 and 95.**

**AbdominalCircumference (AC), *Boys & Girls***


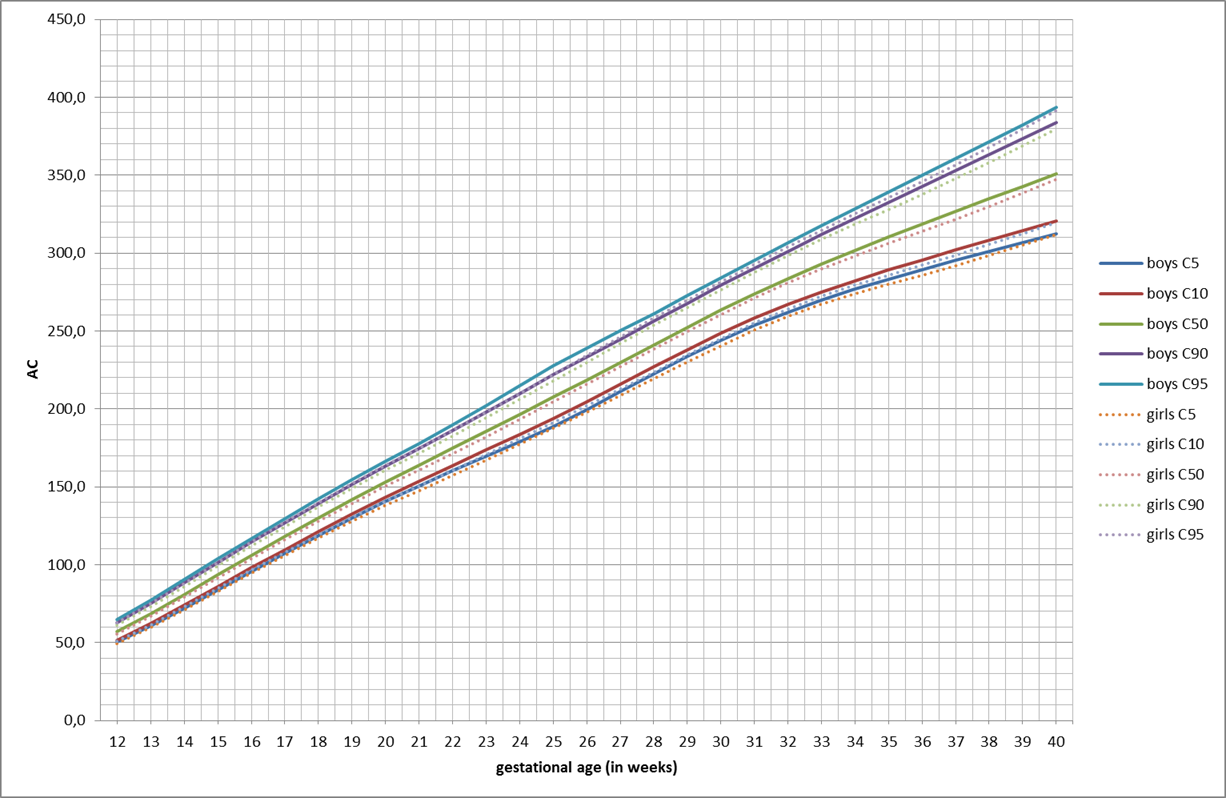


**e-Figure 3.10. Grid reference curves for Abdominal Circumference (AC) in mm for Boys and Girls from 12-40 weeks of gestation. Percentiles 5, 10, 50, 90 and 95.**


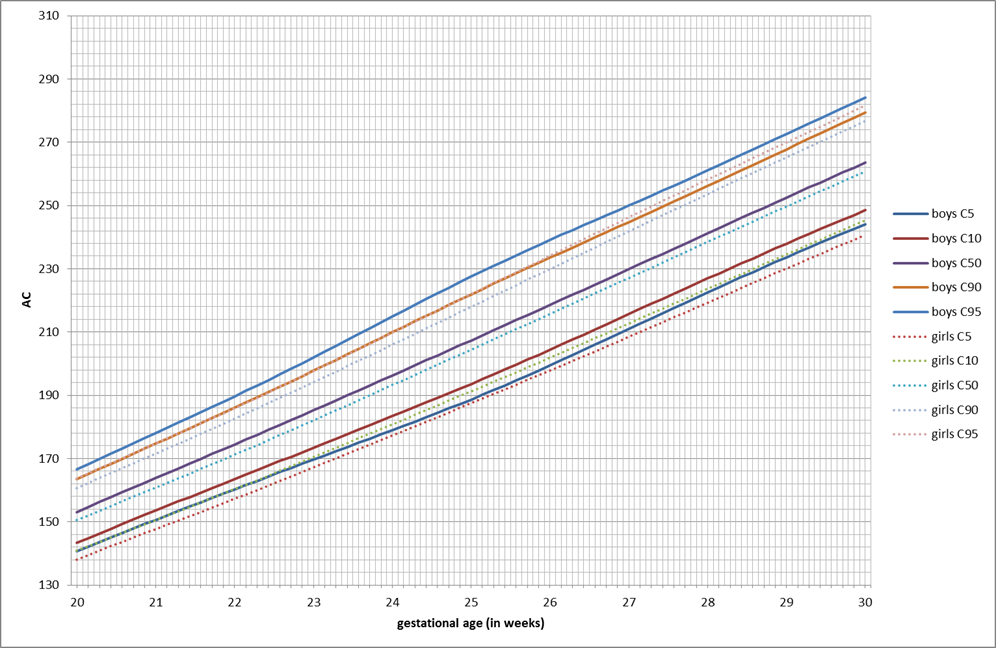


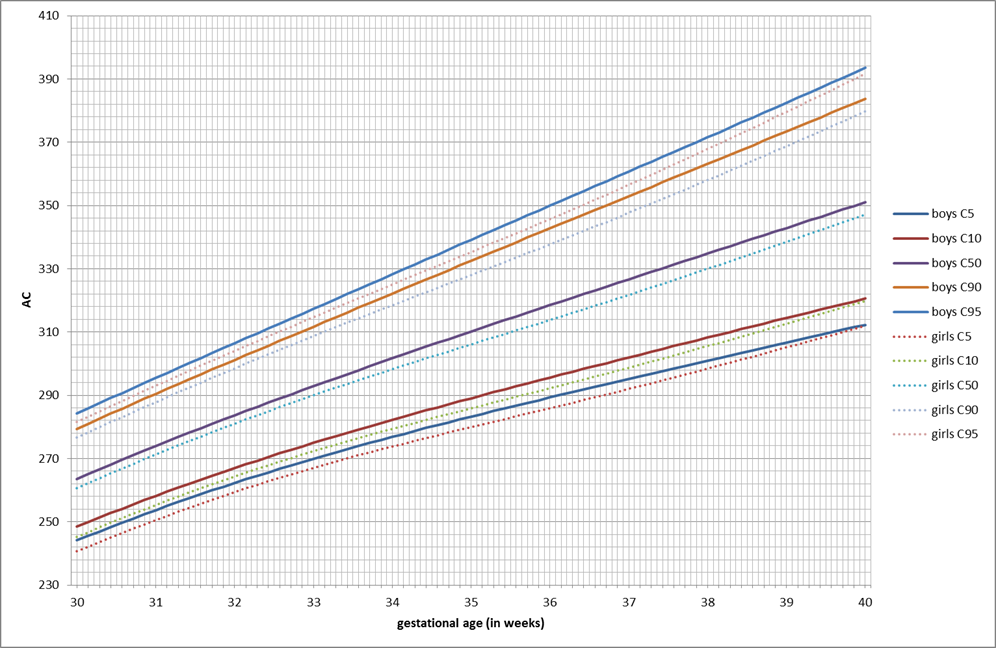


**e-Figure 3.11.a,b. Grid reference curves for Abdominal Circumference (AC) in mm for Boys and Girls: a) from 20-30 weeks (upper); b) from 30-40 weeks of gestation (down). Percentiles 5, 10, 50, 90 and 95.**

**FemurLength (FL), *Boys & Girls***


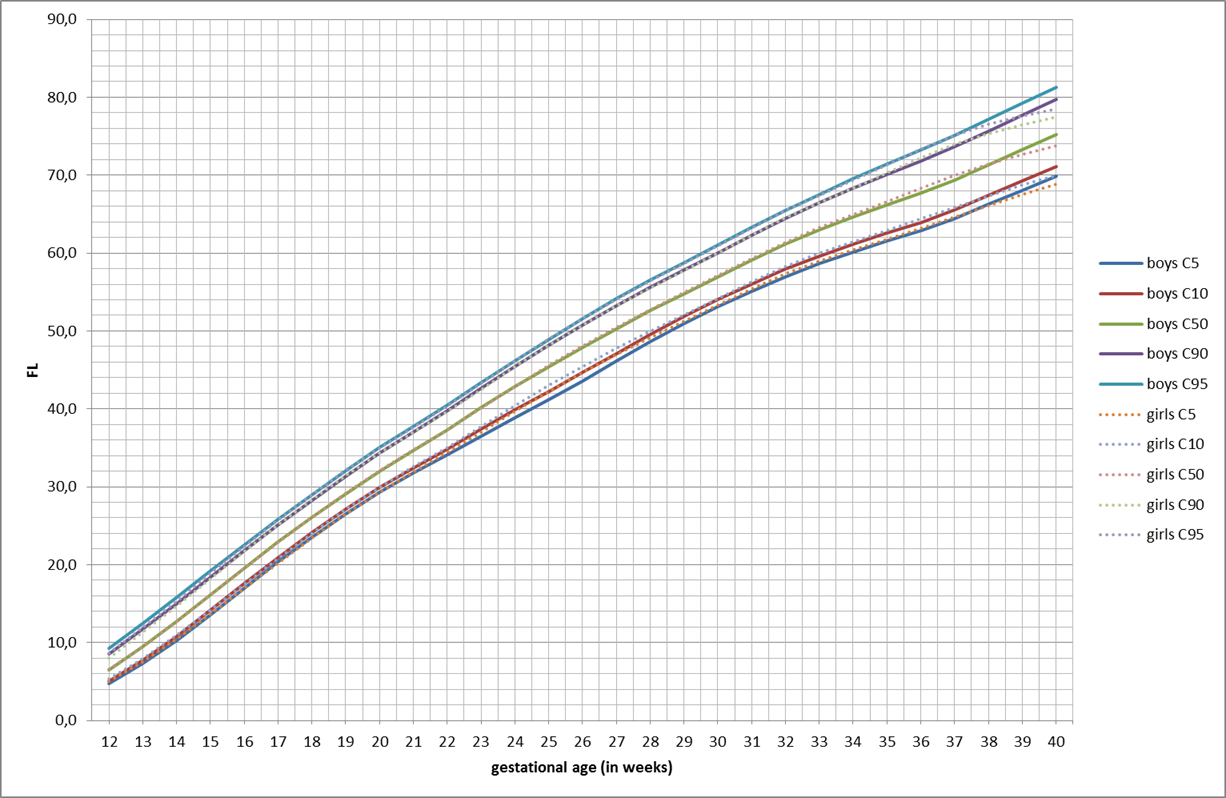


**e-Figure 3.12. Grid reference curves for Femur Length (FL) in mm for Boys and Girls from 12-40 weeks of gestation. Percentiles 5, 10, 50, 90 and 95.**


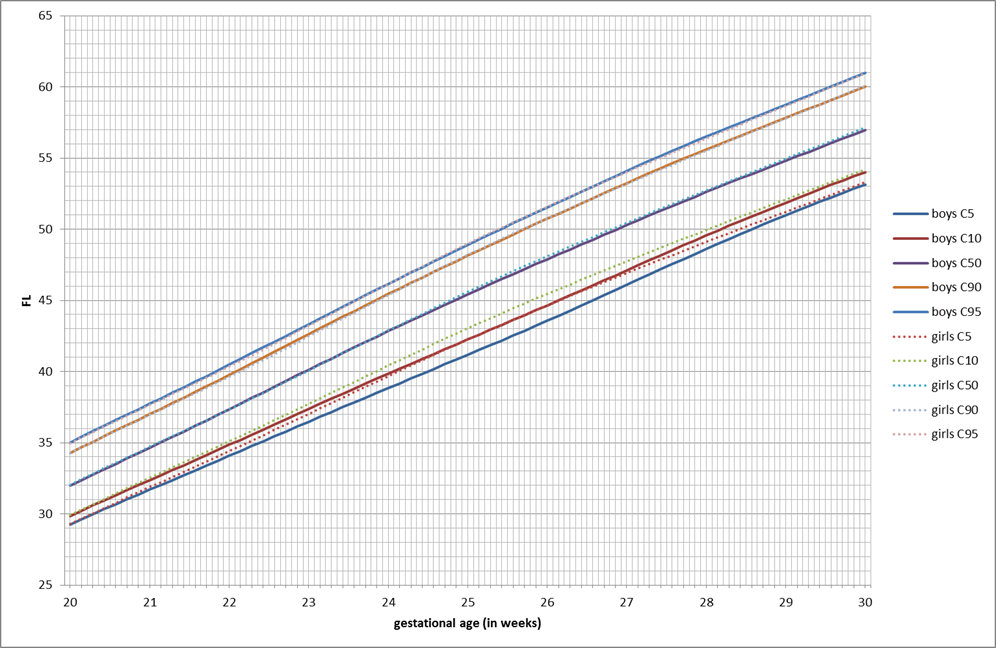


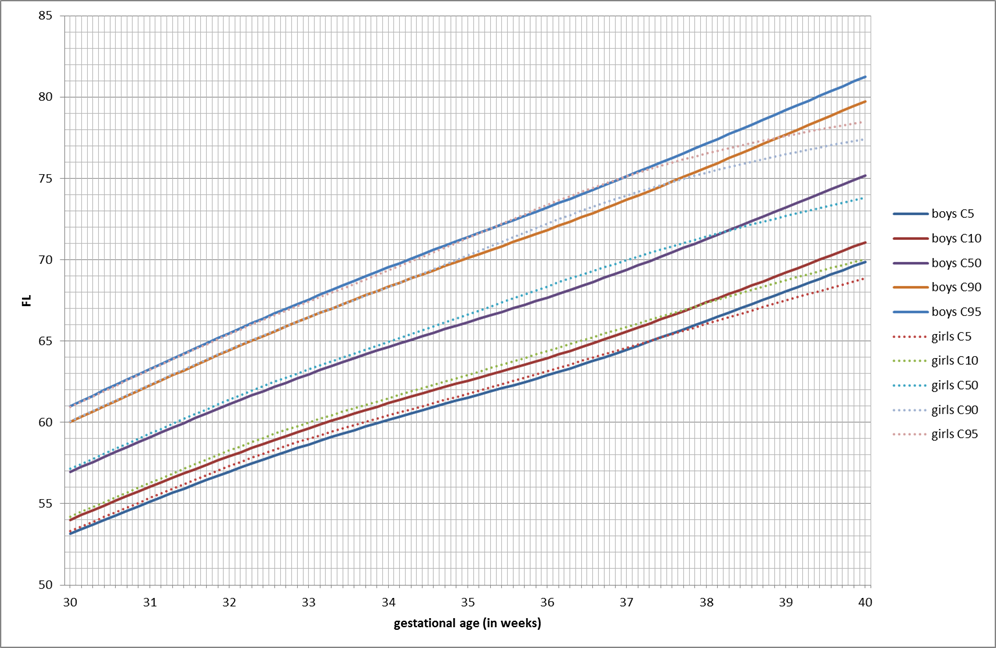


**e-Figure 3.13.a,b. Grid reference curves for Femur Length (FL) in mm for Boys and Girls: a) from 20-30 weeks of gestation (upper); b) from 30-40 weeks of gestation (down). Percentiles 5, 10, 50, 90 and 95.**

**Estimated Fetal Weight (EFW), *Boys & Girls***


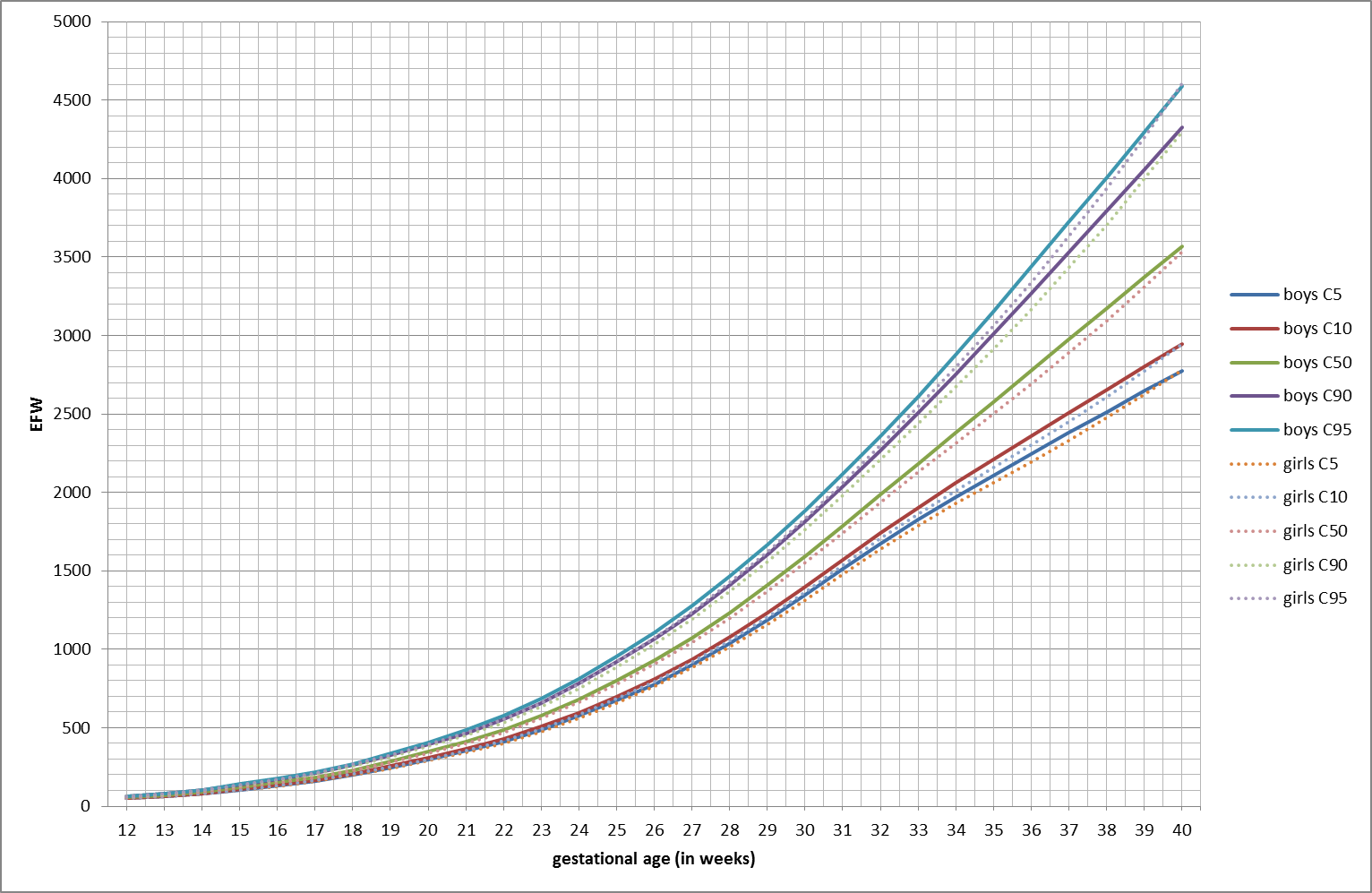


**e-Figure 3.14. Grid reference curves for Estimated Fetal Weight (EFW) in g for Boys and Girls from 12-40 weeks of gestation. Percentiles 5, 10, 50, 90 and 95.**


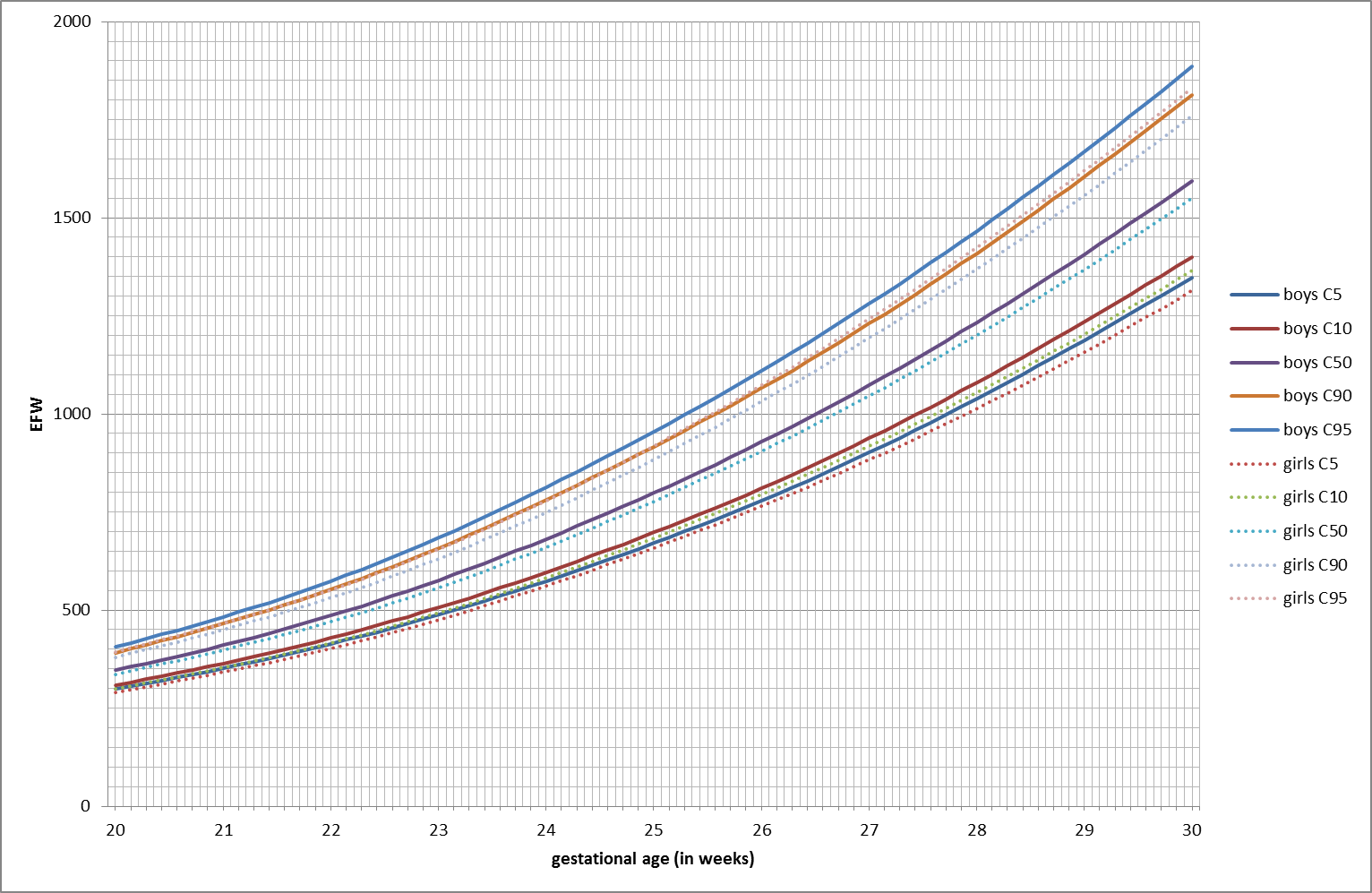


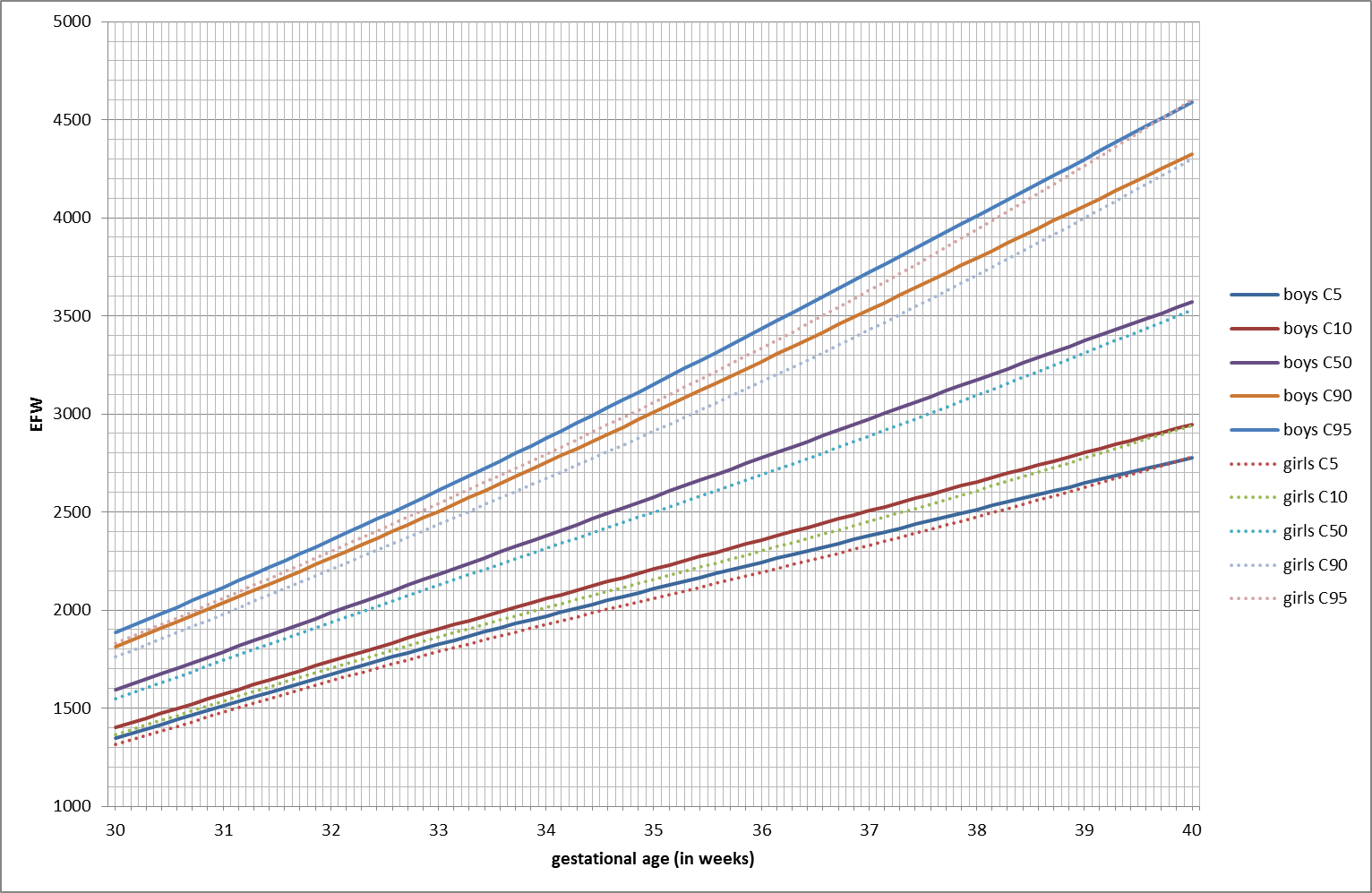


**e-Figure 3.15.a,b. Grid reference curves for Estimated Fetal Weight (EFW) in g for Boys and Girls: a) from 20-30 weeks of gestation (upper); b) from 30-40 weeks of gestation (down). Percentiles 5, 10, 50, 90 and 95.**

**Reference values for BiParietalDiameter (BPD) in mm, *Boys & Girls***

GA = Gestational Age in weeks

| **GA** | **Boys (BPD)** | | | | | **Girls (BPD)** | | | | |
| --- | --- | --- | --- | --- | --- | --- | --- | --- | --- | --- |
|  | **C5** | **C10** | **C50** | **C90** | **C95** | **C5** | **C10** | **C50** | **C90** | **C95** |
| **12** | 16,7 | 17,3 | 19,4 | 21,6 | 22,3 | 16,5 | 17,1 | 19,2 | 21,3 | 21,9 |
| **13** | 20,4 | 21,0 | 23,2 | 25,6 | 26,3 | 20,1 | 20,7 | 22,9 | 25,1 | 25,7 |
| **14** | 24,3 | 25,0 | 27,3 | 29,7 | 30,4 | 24,0 | 24,6 | 26,8 | 29,1 | 29,7 |
| **15** | 28,2 | 28,9 | 31,3 | 33,8 | 34,5 | 27,8 | 28,5 | 30,8 | 33,1 | 33,7 |
| **16** | 32,0 | 32,6 | 35,1 | 37,6 | 38,3 | 31,4 | 32,1 | 34,5 | 36,8 | 37,5 |
| **17** | 35,3 | 36,0 | 38,5 | 41,0 | 41,8 | 34,7 | 35,4 | 37,9 | 40,3 | 41,1 |
| **18** | 38,3 | 39,0 | 41,6 | 44,3 | 45,1 | 37,8 | 38,5 | 41,0 | 43,6 | 44,4 |
| **19** | 41,3 | 42,0 | 44,7 | 47,6 | 48,4 | 40,5 | 41,3 | 44,0 | 46,7 | 47,5 |
| **20** | 44,0 | 44,9 | 47,8 | 50,8 | 51,7 | 43,1 | 43,9 | 46,7 | 49,6 | 50,5 |
| **21** | 46,7 | 47,6 | 50,7 | 54,0 | 55,0 | 45,7 | 46,5 | 49,6 | 52,6 | 53,5 |
| **22** | 49,5 | 50,4 | 53,8 | 57,3 | 58,4 | 48,4 | 49,3 | 52,5 | 55,8 | 56,8 |
| **23** | 52,4 | 53,4 | 57,0 | 60,8 | 61,9 | 51,2 | 52,2 | 55,7 | 59,2 | 60,2 |
| **24** | 55,5 | 56,6 | 60,4 | 64,3 | 65,5 | 54,2 | 55,3 | 58,9 | 62,7 | 63,8 |
| **25** | 58,6 | 59,8 | 63,8 | 67,9 | 69,1 | 57,3 | 58,4 | 62,3 | 66,2 | 67,3 |
| **26** | 61,8 | 63,0 | 67,1 | 71,3 | 72,6 | 60,4 | 61,5 | 65,5 | 69,6 | 70,8 |
| **27** | 64,9 | 66,1 | 70,2 | 74,5 | 75,8 | 63,4 | 64,6 | 68,6 | 72,8 | 74,0 |
| **28** | 67,8 | 69,0 | 73,2 | 77,5 | 78,8 | 66,3 | 67,4 | 71,5 | 75,8 | 77,0 |
| **29** | 70,4 | 71,6 | 75,8 | 80,2 | 81,5 | 69,0 | 70,2 | 74,3 | 78,5 | 79,7 |
| **30** | 72,9 | 74,1 | 78,4 | 82,8 | 84,2 | 71,6 | 72,7 | 76,9 | 81,1 | 82,4 |
| **31** | 75,3 | 76,6 | 81,0 | 85,5 | 86,9 | 74,0 | 75,2 | 79,4 | 83,6 | 84,9 |
| **32** | 77,6 | 78,9 | 83,3 | 87,9 | 89,4 | 76,2 | 77,4 | 81,7 | 86,0 | 87,3 |
| **33** | 79,5 | 80,9 | 85,4 | 90,1 | 91,6 | 78,1 | 79,4 | 83,7 | 88,2 | 89,5 |
| **34** | 81,2 | 82,6 | 87,3 | 92,1 | 93,7 | 79,8 | 81,1 | 85,6 | 90,2 | 91,6 |
| **35** | 82,5 | 84,0 | 88,9 | 93,9 | 95,5 | 81,3 | 82,7 | 87,3 | 92,1 | 93,5 |
| **36** | 83,6 | 85,1 | 90,3 | 95,6 | 97,3 | 82,7 | 84,1 | 88,9 | 93,9 | 95,3 |
| **37** | 84,6 | 86,2 | 91,7 | 97,4 | 99,2 | 84,1 | 85,5 | 90,5 | 95,6 | 97,2 |
| **38** | 85,7 | 87,4 | 93,2 | 99,3 | 101,2 | 85,5 | 87,0 | 92,1 | 97,4 | 99,0 |
| **39** | 87,0 | 88,8 | 94,9 | 101,2 | 103,3 | 86,9 | 88,4 | 93,7 | 99,3 | 100,9 |
| **40** | 88,4 | 90,3 | 96,6 | 103,2 | 105,3 | 88,2 | 89,8 | 95,3 | 101,1 | 102,8 |

e-Table 8. Reference values in mm for fetal biparietal diameter (BPD) for boys and girls for each gestational week for the median and 5^th^, 10^th^, 90^th^ and 95^th^ centiles. GA = gestational weeks.

**Reference values for HeadCircumference (HC) in mm, *Boys & Girls***

GA = Gestational Age in weeks

| **GA** | **Boys (HC)** | | | | | **Girls (HC)** | | | | |
| --- | --- | --- | --- | --- | --- | --- | --- | --- | --- | --- |
|  | **C5** | **C10** | **C50** | **C90** | **C95** | **C5** | **C10** | **C50** | **C90** | **C95** |
| **12** | 61,3 | 63,3 | 70,3 | 77,4 | 79,4 | 61,9 | 63,6 | 69,7 | 76,1 | 78,1 |
| **13** | 74,4 | 76,5 | 83,8 | 91,1 | 93,2 | 74,3 | 76,1 | 82,6 | 89,5 | 91,6 |
| **14** | 88,2 | 90,3 | 97,8 | 105,3 | 107,5 | 87,3 | 89,2 | 96,1 | 103,3 | 105,4 |
| **15** | 101,9 | 104,1 | 111,6 | 119,2 | 121,4 | 100,6 | 102,6 | 109,6 | 117,0 | 119,2 |
| **16** | 115,2 | 117,4 | 124,9 | 132,6 | 134,8 | 113,6 | 115,6 | 122,8 | 130,4 | 132,6 |
| **17** | 128,0 | 130,2 | 137,8 | 145,6 | 147,8 | 126,3 | 128,3 | 135,7 | 143,3 | 145,6 |
| **18** | 140,3 | 142,5 | 150,3 | 158,2 | 160,5 | 138,5 | 140,7 | 148,2 | 156,0 | 158,3 |
| **19** | 152,2 | 154,5 | 162,6 | 170,7 | 173,1 | 150,1 | 152,3 | 160,0 | 168,0 | 170,4 |
| **20** | 163,4 | 165,8 | 174,3 | 182,9 | 185,4 | 160,7 | 163,0 | 171,0 | 179,2 | 181,6 |
| **21** | 173,9 | 176,4 | 185,5 | 194,6 | 197,3 | 171,1 | 173,4 | 181,7 | 190,2 | 192,8 |
| **22** | 184,2 | 187,0 | 196,8 | 206,6 | 209,5 | 181,5 | 183,9 | 192,6 | 201,5 | 204,2 |
| **23** | 194,8 | 197,9 | 208,5 | 219,1 | 222,2 | 191,9 | 194,5 | 203,7 | 213,2 | 216,0 |
| **24** | 205,6 | 208,9 | 220,3 | 231,6 | 234,8 | 202,3 | 205,1 | 215,0 | 225,2 | 228,3 |
| **25** | 216,2 | 219,7 | 231,7 | 243,6 | 247,0 | 212,5 | 215,5 | 226,2 | 237,3 | 240,6 |
| **26** | 226,8 | 230,4 | 242,8 | 255,0 | 258,5 | 222,5 | 225,8 | 237,2 | 249,1 | 252,7 |
| **27** | 237,4 | 241,1 | 253,6 | 266,0 | 269,6 | 232,7 | 236,1 | 248,0 | 260,5 | 264,2 |
| **28** | 247,7 | 251,4 | 264,0 | 276,5 | 280,2 | 243,0 | 246,4 | 258,5 | 271,1 | 274,8 |
| **29** | 257,1 | 260,7 | 273,4 | 286,2 | 290,0 | 252,7 | 256,1 | 268,1 | 280,6 | 284,3 |
| **30** | 265,3 | 269,0 | 282,0 | 295,1 | 299,0 | 261,6 | 265,0 | 277,0 | 289,5 | 293,2 |
| **31** | 273,0 | 276,8 | 290,0 | 303,7 | 307,7 | 269,6 | 273,1 | 285,5 | 298,3 | 302,1 |
| **32** | 280,2 | 284,0 | 297,6 | 311,9 | 316,3 | 276,5 | 280,2 | 293,2 | 306,7 | 310,8 |
| **33** | 286,5 | 290,4 | 304,4 | 319,4 | 324,1 | 282,6 | 286,4 | 300,1 | 314,4 | 318,7 |
| **34** | 292,1 | 296,1 | 310,3 | 326,0 | 331,0 | 288,0 | 292,0 | 306,2 | 321,1 | 325,5 |
| **35** | 297,7 | 301,6 | 315,9 | 332,2 | 337,5 | 292,6 | 296,7 | 311,3 | 326,5 | 331,0 |
| **36** | 302,9 | 306,8 | 321,2 | 338,2 | 343,9 | 296,6 | 300,7 | 315,5 | 330,9 | 335,5 |
| **37** | 307,3 | 311,3 | 326,1 | 344,1 | 350,4 | 301,0 | 305,2 | 320,1 | 335,8 | 340,4 |
| **38** | 311,9 | 316,0 | 331,1 | 350,4 | 357,5 | 306,5 | 310,7 | 325,9 | 341,7 | 346,4 |
| **39** | 317,3 | 321,4 | 336,7 | 357,1 | 365,2 | 312,8 | 317,1 | 332,4 | 348,5 | 353,3 |
| **40** | 323,5 | 327,6 | 342,9 | 364,2 | 373,1 | 319,4 | 323,7 | 339,2 | 355,4 | 360,2 |

e-Table 9. Reference values in mm for fetal head-circumference (HC) for boys and girls for each gestational week for the median and 5^th^, 10^th^, 90^th^ and 95^th^ centiles. GA = gestational weeks.

**Reference values for AbdominalCircumference (AC) in mm, *Boys & Girls***

GA = Gestational Age in weeks

| **GA** | **Boys (AC)** | | | | | **Girls (AC)** | | | | |
| --- | --- | --- | --- | --- | --- | --- | --- | --- | --- | --- |
|  | **C5** | **C10** | **C50** | **C90** | **C95** | **C5** | **C10** | **C50** | **C90** | **C95** |
| **12** | 50,1 | 51,6 | 57,0 | 62,9 | 64,7 | 49,4 | 50,7 | 55,5 | 61,0 | 62,7 |
| **13** | 60,7 | 62,4 | 68,6 | 75,2 | 77,2 | 59,8 | 61,2 | 66,7 | 72,9 | 74,8 |
| **14** | 72,1 | 73,9 | 80,8 | 88,1 | 90,3 | 71,1 | 72,7 | 78,9 | 85,7 | 87,8 |
| **15** | 83,9 | 86,0 | 93,4 | 101,4 | 103,7 | 82,9 | 84,7 | 91,5 | 99,0 | 101,3 |
| **16** | 95,7 | 97,9 | 105,9 | 114,4 | 116,9 | 94,5 | 96,5 | 103,9 | 112,0 | 114,4 |
| **17** | 107,4 | 109,7 | 118,1 | 127,0 | 129,6 | 105,9 | 108,1 | 116,0 | 124,6 | 127,2 |
| **18** | 118,8 | 121,2 | 130,0 | 139,4 | 142,2 | 117,1 | 119,4 | 127,9 | 137,0 | 139,7 |
| **19** | 130,0 | 132,5 | 141,8 | 151,7 | 154,6 | 127,9 | 130,4 | 139,5 | 149,1 | 152,0 |
| **20** | 140,6 | 143,3 | 153,1 | 163,4 | 166,5 | 138,1 | 140,8 | 150,5 | 160,7 | 163,7 |
| **21** | 150,6 | 153,6 | 163,9 | 174,7 | 178,0 | 147,7 | 150,6 | 160,8 | 171,6 | 174,8 |
| **22** | 160,3 | 163,5 | 174,5 | 186,0 | 189,7 | 157,3 | 160,3 | 171,2 | 182,6 | 186,0 |
| **23** | 169,7 | 173,4 | 185,2 | 197,7 | 202,0 | 167,2 | 170,5 | 182,1 | 194,1 | 197,7 |
| **24** | 179,0 | 183,4 | 196,3 | 209,9 | 214,9 | 177,3 | 180,8 | 193,2 | 206,0 | 209,8 |
| **25** | 188,7 | 193,6 | 207,4 | 221,9 | 227,5 | 187,5 | 191,3 | 204,4 | 218,0 | 222,0 |
| **26** | 199,5 | 204,4 | 218,6 | 233,5 | 239,1 | 197,8 | 201,9 | 215,7 | 229,9 | 234,2 |
| **27** | 211,0 | 215,7 | 229,8 | 244,8 | 250,0 | 208,5 | 212,7 | 227,1 | 241,8 | 246,3 |
| **28** | 222,5 | 226,9 | 241,2 | 256,2 | 261,1 | 219,3 | 223,6 | 238,5 | 253,6 | 258,2 |
| **29** | 233,6 | 237,9 | 252,5 | 267,8 | 272,6 | 230,1 | 234,6 | 249,7 | 265,2 | 269,9 |
| **30** | 244,1 | 248,5 | 263,5 | 279,3 | 284,2 | 240,7 | 245,3 | 260,7 | 276,7 | 281,6 |
| **31** | 253,7 | 258,2 | 274,0 | 290,5 | 295,5 | 250,6 | 255,3 | 271,3 | 287,8 | 293,0 |
| **32** | 262,3 | 267,0 | 283,7 | 301,2 | 306,5 | 259,4 | 264,3 | 281,0 | 298,5 | 304,0 |
| **33** | 269,9 | 274,9 | 292,8 | 311,7 | 317,3 | 267,1 | 272,3 | 290,0 | 308,7 | 314,7 |
| **34** | 276,8 | 282,2 | 301,7 | 322,1 | 328,2 | 273,8 | 279,4 | 298,2 | 318,5 | 325,0 |
| **35** | 283,2 | 289,1 | 310,2 | 332,5 | 339,2 | 279,9 | 285,9 | 306,1 | 328,0 | 335,3 |
| **36** | 289,3 | 295,6 | 318,5 | 342,8 | 350,0 | 285,9 | 292,2 | 313,8 | 337,7 | 345,7 |
| **37** | 295,2 | 302,0 | 326,7 | 353,0 | 360,8 | 292,0 | 298,7 | 321,7 | 347,7 | 356,5 |
| **38** | 301,0 | 308,2 | 334,8 | 363,2 | 371,6 | 298,4 | 305,6 | 330,0 | 358,0 | 367,8 |
| **39** | 306,7 | 314,4 | 342,9 | 373,4 | 382,5 | 305,1 | 312,6 | 338,5 | 368,8 | 379,6 |
| **40** | 312,3 | 320,5 | 351,0 | 383,8 | 393,5 | 311,9 | 319,8 | 347,1 | 379,8 | 391,7 |

e-Table 10. Reference values in mm for fetal abdominal circumference (AC) for boys and girls for each gestational week for the median and 5^th^, 10^th^, 90^th^ and 95^th^ centiles. GA = gestational weeks.

**Reference values for FemurLength (FL) in mm, *Boys & Girls***

GA = Gestational Age in weeks

| **GA** | **Boys (FL)** | | | | | **Girls (FL)** | | | | |
| --- | --- | --- | --- | --- | --- | --- | --- | --- | --- | --- |
|  | **C5** | **C10** | **C50** | **C90** | **C95** | **C5** | **C10** | **C50** | **C90** | **C95** |
| **12** | 4,7 | 5,0 | 6,5 | 8,5 | 9,3 | 5,0 | 5,3 | 6,6 | 8,1 | 8,6 |
| **13** | 7,3 | 7,7 | 9,5 | 11,7 | 12,5 | 7,5 | 8,0 | 9,5 | 11,3 | 11,9 |
| **14** | 10,3 | 10,8 | 12,8 | 15,0 | 15,8 | 10,5 | 11,0 | 12,7 | 14,7 | 15,3 |
| **15** | 13,6 | 14,1 | 16,2 | 18,4 | 19,2 | 13,6 | 14,2 | 16,1 | 18,2 | 18,9 |
| **16** | 17,0 | 17,6 | 19,6 | 21,8 | 22,5 | 16,9 | 17,5 | 19,5 | 21,6 | 22,3 |
| **17** | 20,3 | 20,9 | 22,9 | 25,1 | 25,8 | 20,2 | 20,8 | 22,8 | 25,0 | 25,7 |
| **18** | 23,5 | 24,1 | 26,1 | 28,3 | 29,0 | 23,4 | 24,0 | 26,1 | 28,3 | 29,0 |
| **19** | 26,5 | 27,1 | 29,1 | 31,4 | 32,1 | 26,5 | 27,1 | 29,2 | 31,4 | 32,1 |
| **20** | 29,3 | 29,9 | 32,0 | 34,3 | 35,0 | 29,3 | 29,9 | 32,1 | 34,3 | 35,0 |
| **21** | 31,7 | 32,4 | 34,7 | 37,0 | 37,7 | 31,9 | 32,5 | 34,7 | 37,0 | 37,7 |
| **22** | 34,1 | 34,8 | 37,3 | 39,8 | 40,5 | 34,4 | 35,1 | 37,3 | 39,6 | 40,3 |
| **23** | 36,5 | 37,4 | 40,1 | 42,6 | 43,3 | 37,0 | 37,7 | 40,1 | 42,5 | 43,2 |
| **24** | 38,8 | 39,9 | 42,8 | 45,4 | 46,2 | 39,7 | 40,4 | 42,9 | 45,4 | 46,2 |
| **25** | 41,2 | 42,3 | 45,4 | 48,1 | 48,9 | 42,2 | 43,0 | 45,6 | 48,2 | 49,0 |
| **26** | 43,6 | 44,7 | 47,9 | 50,7 | 51,5 | 44,7 | 45,5 | 48,1 | 50,8 | 51,6 |
| **27** | 46,1 | 47,1 | 50,3 | 53,2 | 54,1 | 46,9 | 47,7 | 50,4 | 53,2 | 54,0 |
| **28** | 48,6 | 49,6 | 52,6 | 55,6 | 56,5 | 49,1 | 50,0 | 52,7 | 55,5 | 56,4 |
| **29** | 51,0 | 51,9 | 54,8 | 57,8 | 58,8 | 51,2 | 52,1 | 55,0 | 57,8 | 58,7 |
| **30** | 53,1 | 54,0 | 57,0 | 60,0 | 61,0 | 53,3 | 54,2 | 57,1 | 60,1 | 60,9 |
| **31** | 55,1 | 56,0 | 59,1 | 62,3 | 63,3 | 55,3 | 56,3 | 59,3 | 62,3 | 63,2 |
| **32** | 57,0 | 57,9 | 61,1 | 64,4 | 65,5 | 57,3 | 58,3 | 61,4 | 64,5 | 65,4 |
| **33** | 58,6 | 59,6 | 63,0 | 66,4 | 67,6 | 59,0 | 60,0 | 63,3 | 66,5 | 67,4 |
| **34** | 60,2 | 61,2 | 64,6 | 68,3 | 69,5 | 60,4 | 61,5 | 64,9 | 68,3 | 69,3 |
| **35** | 61,5 | 62,6 | 66,2 | 70,1 | 71,4 | 61,8 | 62,9 | 66,6 | 70,2 | 71,3 |
| **36** | 62,9 | 64,0 | 67,7 | 71,8 | 73,2 | 63,2 | 64,4 | 68,4 | 72,2 | 73,4 |
| **37** | 64,5 | 65,6 | 69,4 | 73,7 | 75,1 | 64,6 | 65,9 | 70,0 | 73,9 | 75,1 |
| **38** | 66,2 | 67,4 | 71,3 | 75,7 | 77,1 | 66,0 | 67,3 | 71,4 | 75,3 | 76,5 |
| **39** | 68,0 | 69,2 | 73,2 | 77,7 | 79,2 | 67,5 | 68,7 | 72,7 | 76,5 | 77,6 |
| **40** | 69,9 | 71,1 | 75,2 | 79,7 | 81,3 | 68,8 | 70,0 | 73,8 | 77,4 | 78,5 |

e-Table 11. Reference values in mm for fetal femur length (FL) for boys and girls for each gestational week for the median and 5^th^, 10^th^, 90^th^ and 95^th^ centiles. GA = gestational weeks.

**Reference values for Estimated Fetal Weight (EFW) in gram, *Boys & Girls***

GA = Gestational Age in weeks

| **GA** | **Boys (EFW)** | | | | | **Girls (EFW)** | | | | |
| --- | --- | --- | --- | --- | --- | --- | --- | --- | --- | --- |
|  | **C5** | **C10** | **C50** | **C90** | **C95** | **C5** | **C10** | **C50** | **C90** | **C95** |
| **12** | 50 | 51 | 57 | 63 | 65 | 49 | 50 | 55 | 61 | 63 |
| **13** | 60 | 62 | 69 | 78 | 81 | 61 | 62 | 69 | 76 | 79 |
| **14** | 77 | 80 | 89 | 101 | 105 | 79 | 82 | 90 | 101 | 104 |
| **15** | 104 | 107 | 120 | 136 | 142 | 103 | 106 | 117 | 131 | 136 |
| **16** | 131 | 136 | 152 | 172 | 179 | 128 | 132 | 147 | 165 | 171 |
| **17** | 159 | 165 | 185 | 208 | 216 | 159 | 164 | 183 | 206 | 213 |
| **18** | 199 | 205 | 230 | 260 | 269 | 198 | 204 | 229 | 257 | 266 |
| **19** | 248 | 256 | 287 | 324 | 336 | 242 | 250 | 281 | 316 | 328 |
| **20** | 298 | 308 | 346 | 391 | 406 | 290 | 299 | 337 | 380 | 394 |
| **21** | 352 | 364 | 410 | 465 | 482 | 342 | 354 | 398 | 450 | 466 |
| **22** | 414 | 429 | 486 | 552 | 574 | 402 | 416 | 470 | 531 | 551 |
| **23** | 488 | 506 | 576 | 658 | 685 | 475 | 493 | 557 | 631 | 655 |
| **24** | 574 | 596 | 681 | 781 | 813 | 561 | 582 | 660 | 750 | 779 |
| **25** | 671 | 698 | 799 | 917 | 955 | 658 | 684 | 777 | 884 | 919 |
| **26** | 779 | 810 | 928 | 1065 | 1110 | 765 | 795 | 905 | 1032 | 1073 |
| **27** | 901 | 938 | 1073 | 1230 | 1280 | 883 | 918 | 1046 | 1193 | 1241 |
| **28** | 1038 | 1080 | 1233 | 1410 | 1467 | 1014 | 1054 | 1201 | 1369 | 1424 |
| **29** | 1188 | 1235 | 1407 | 1605 | 1669 | 1158 | 1204 | 1369 | 1558 | 1620 |
| **30** | 1348 | 1400 | 1593 | 1814 | 1886 | 1314 | 1366 | 1550 | 1761 | 1831 |
| **31** | 1511 | 1571 | 1788 | 2035 | 2116 | 1478 | 1536 | 1743 | 1979 | 2059 |
| **32** | 1672 | 1739 | 1985 | 2265 | 2357 | 1638 | 1704 | 1937 | 2206 | 2298 |
| **33** | 1825 | 1902 | 2182 | 2504 | 2610 | 1788 | 1863 | 2128 | 2437 | 2544 |
| **34** | 1970 | 2058 | 2380 | 2752 | 2876 | 1927 | 2012 | 2314 | 2671 | 2796 |
| **35** | 2108 | 2209 | 2577 | 3007 | 3152 | 2060 | 2156 | 2500 | 2912 | 3059 |
| **36** | 2244 | 2358 | 2775 | 3267 | 3434 | 2193 | 2301 | 2689 | 3163 | 3336 |
| **37** | 2379 | 2506 | 2974 | 3530 | 3720 | 2330 | 2452 | 2887 | 3428 | 3629 |
| **38** | 2513 | 2655 | 3174 | 3795 | 4009 | 2475 | 2610 | 3095 | 3707 | 3940 |
| **39** | 2647 | 2802 | 3373 | 4060 | 4299 | 2626 | 2774 | 3310 | 3999 | 4265 |
| **40** | 2776 | 2946 | 3570 | 4325 | 4591 | 2780 | 2943 | 3531 | 4300 | 4605 |

e-Table 12. Reference values in g for Estimated Fetal Weight (EFW) for boys and girls for each gestational week for the median and 5^th^, 10^th^, 90^th^ and 95^th^ centiles. GA = gestational weeks.
