# Supplementary material for: Sex differences in fetal growth and immediate birth outcomes in a low-risk Caucasian population
Source: Biol Sex Differ. 2019 Sep 9;10:48. doi: 10.1186/s13293-019-0261-7 (PMC6734449; doi:10.1186/s13293-019-0261-7)
Supplement: Supplementary file 5 — Boys vs Girls combined centiles and real values. (DOCX 35 kb) [file 13293_2019_261_MOESM5_ESM.docx]

# Additional file 5: Comparison centiles Boys & Girls in GA weeks 20, 25, 30, 35 and 40.

BiParietal Diamater (BPD) for Boys and Girls in mm

| **Centiles** | **GA** | **Boys** | **Girls** | **SE** | **p-value** |
| --- | --- | --- | --- | --- | --- |
| **C5** | 20 | 44,0 | 43,1 | 0,059 | <0,001 |
|  | 25 | 58,6 | 57,3 | 0,4 | <0,001 |
|  | 30 | 72,9 | 71,6 | 0,1 | <0,001 |
|  | 35 | 82,5 | 81,3 | 0,3 | <0,001 |
|  | 40 | 88,4 | 88,2 | 1,1 | *0,39* |
| **C10** | 20 | 44,9 | 43,9 | 0,059 | <0,001 |
|  | 25 | 59,8 | 58,4 | 0,4 | <0,001 |
|  | 30 | 74,1 | 72,7 | 0,1 | <0,001 |
|  | 35 | 84,0 | 82,7 | 0,3 | <0,001 |
|  | 40 | 90,3 | 89,8 | 1,1 | *0,36* |
| **C50** | 20 | 47,8 | 46,7 | 0,059 | <0,001 |
|  | 25 | 63,8 | 62,3 | 0,4 | <0,001 |
|  | 30 | 78,4 | 76,9 | 0,1 | <0,001 |
|  | 35 | 88,9 | 87,3 | 0,3 | <0,001 |
|  | 40 | 96,6 | 95,3 | 1,1 | *0,21* |
| **C90** | 20 | 50,8 | 49,6 | 0,059 | <0,001 |
|  | 25 | 67,9 | 66,2 | 0,4 | <0,001 |
|  | 30 | 82,8 | 81,1 | 0,1 | <0,001 |
|  | 35 | 93,9 | 92,1 | 0,3 | <0,001 |
|  | 40 | 103,2 | 101,1 | 1,1 | *0,07* |
| **C95** | 20 | 51,7 | 50,5 | 0,059 | <0,001 |
|  | 25 | 69,1 | 67,3 | 0,4 | <0,001 |
|  | 30 | 84,2 | 82,4 | 0,1 | <0,001 |
|  | 35 | 95,5 | 93,5 | 0,3 | <0,001 |
|  | 40 | 105,3 | 102,8 | 1,1 | 0,03 |

e-Table 13. Centiles = C (C5 =P5; C10=P10; C50=P50; C90=P90; C95=P95). GA = Gestational Age in weeks. SE = Standard Error

Head Circumference (HC) for Boys and Girls in mm

| **Centiles** | **GA** | **Boys** | **Girls** | **SE** | **p-value** |
| --- | --- | --- | --- | --- | --- |
| **C5** | 20 | 163,4 | 160,7 | 0,177 | <0,001 |
|  | 25 | 216,2 | 212,5 | 1,336 | 0,008 |
|  | 30 | 265,3 | 261,6 | 0,327 | <0,001 |
|  | 35 | 297,7 | 292,6 | 1,038 | <0,001 |
|  | 40 | 323,5 | 319,4 | 2,696 | *0,13* |
| **C10** | 20 | 165,8 | 163,0 | 0,177 | <0,001 |
|  | 25 | 219,7 | 215,5 | 1,336 | 0,003 |
|  | 30 | 269,0 | 265,0 | 0,327 | <0,001 |
|  | 35 | 301,6 | 296,7 | 1,038 | <0,001 |
|  | 40 | 327,6 | 323,7 | 2,696 | *0,15* |
| **C50** | 20 | 174,3 | 171,0 | 0,177 | <0,001 |
|  | 25 | 231,7 | 226,2 | 1,336 | <0,001 |
|  | 30 | 282,0 | 277,0 | 0,327 | <0,001 |
|  | 35 | 315,9 | 311,3 | 1,038 | <0,001 |
|  | 40 | 342,9 | 339,2 | 2,696 | *0,16* |
| **C90** | 20 | 182,9 | 179,2 | 0,177 | <0,001 |
|  | 25 | 243,6 | 237,3 | 1,336 | <0,001 |
|  | 30 | 295,1 | 289,5 | 0,327 | <0,001 |
|  | 35 | 332,2 | 326,5 | 1,038 | <0,001 |
|  | 40 | 364,2 | 355,4 | 2,696 | 0,002 |
| **C95** | 20 | 185,4 | 181,6 | 0,177 | <0,001 |
|  | 25 | 247,0 | 240,6 | 1,336 | <0,001 |
|  | 30 | 299,0 | 293,2 | 0,327 | <0,001 |
|  | 35 | 337,5 | 331,0 | 1,038 | <0,001 |
|  | 40 | 373,1 | 360,2 | 2,696 | <0,001 |

e-Table 14. Centiles = C (C5 =P5; C10=P10; C50=P50; C90=P90; C95=P95). GA = Gestational Age in weeks. SE = Standard Error

Abdominal Circumference (AC) for Boys and Girls in mm

| **Centiles** | **GA** | **Boys** | **Girls** | **SE** | **p-value** |
| --- | --- | --- | --- | --- | --- |
| **C5** | 20 | 140,6 | 138,1 | 0,202 | <0,001 |
|  | 25 | 188,7 | 187,5 | 1,702 | *0,32* |
|  | 30 | 244,1 | 240,7 | 0,373 | <0,001 |
|  | 35 | 283,2 | 279,9 | 1,538 | 0,04 |
|  | 40 | 312,3 | 311,9 | 6,380 | *0,40* |
| **C10** | 20 | 143,3 | 140,8 | 0,202 | <0,001 |
|  | 25 | 193,6 | 191,3 | 1,702 | *0,16* |
|  | 30 | 248,5 | 245,3 | 0,373 | <0,001 |
|  | 35 | 289,1 | 285,9 | 1,538 | 0,05 |
|  | 40 | 320,5 | 319,8 | 6,380 | *0,40* |
| **C50** | 20 | 153,1 | 150,5 | 0,202 | <0,001 |
|  | 25 | 207,4 | 204,4 | 1,702 | *0,09* |
|  | 30 | 263,5 | 260,7 | 0,373 | <0,001 |
|  | 35 | 310,2 | 306,1 | 1,538 | 0,01 |
|  | 40 | 351,0 | 347,1 | 6,380 | *0,33* |
| **C90** | 20 | 163,4 | 160,7 | 0,202 | <0,001 |
|  | 25 | 221,9 | 218,0 | 1,702 | *0,03* |
|  | 30 | 279,3 | 276,7 | 0,373 | <0,001 |
|  | 35 | 332,5 | 328,0 | 1,538 | 0,006 |
|  | 40 | 383,8 | 379,8 | 6,380 | *0,33* |
| **C95** | 20 | 166,5 | 163,7 | 0,202 | <0,001 |
|  | 25 | 227,5 | 222,0 | 1,702 | 0,002 |
|  | 30 | 284,2 | 281,6 | 0,373 | <0,001 |
|  | 35 | 339,2 | 335,3 | 1,538 | 0,02 |
|  | 40 | 393,5 | 391,7 | 6,380 | *0,38* |

e-Table 15. Centiles = C (C5 =P5; C10=P10; C50=P50; C90=P90; C95=P95). GA = Gestational Age in weeks. SE = Standard Error

Femur length (FL) for Boys and Girls in mm

| **Centiles** | **GA** | **Boys** | **Girls** | **SE** | **p-value** |
| --- | --- | --- | --- | --- | --- |
| **C5** | 20 | 29,3 | 29,3 | 0,046 | *0,36* |
|  | 25 | 41,2 | 42,2 | 0,378 | 0,007 |
|  | 30 | 53,1 | 53,3 | 0,073 | 0,03 |
|  | 35 | 61,5 | 61,8 | 0,262 | *0,27* |
|  | 40 | 69,9 | 68,8 | 1,167 | *0,27* |
| **C10** | 20 | 29,9 | 29,9 | 0,046 | *0,20* |
|  | 25 | 42,3 | 43,0 | 0,378 | 0,05 |
|  | 30 | 54,0 | 54,2 | 0,073 | 0,02 |
|  | 35 | 62,6 | 62,9 | 0,262 | *0,17* |
|  | 40 | 71,1 | 70,0 | 1,167 | *0,27* |
| **C50** | 20 | 32,0 | 32,1 | 0,046 | *0,08* |
|  | 25 | 45,4 | 45,6 | 0,378 | *0,36* |
|  | 30 | 57,0 | 57,1 | 0,073 | 0,02 |
|  | 35 | 66,2 | 66,6 | 0,262 | *0,08* |
|  | 40 | 75,2 | 73,8 | 1,167 | *0,20* |
| **C90** | 20 | 34,3 | 34,3 | 0,046 | *0,40* |
|  | 25 | 48,1 | 48,2 | 0,378 | *0,40* |
|  | 30 | 60,0 | 60,1 | 0,073 | *0,38* |
|  | 35 | 70,1 | 70,2 | 0,262 | *0,34* |
|  | 40 | 79,7 | 77,4 | 1,167 | *0,06* |
| **C95** | 20 | 35,0 | 35,0 | 0,046 | *0,26* |
|  | 25 | 48,9 | 49,0 | 0,378 | *0,39* |
|  | 30 | 61,0 | 60,9 | 0,073 | *0,31* |
|  | 35 | 71,4 | 71,3 | 0,262 | *0,39* |
|  | 40 | 81,3 | 78,5 | 1,167 | 0,02 |

e-Table 16. Centiles = C (C5 =P5; C10=P10; C50=P50; C90=P90; C95=P95). GA = Gestational Age in weeks. SE = Standard Error

Estimated Fetal Weight (EFW) for Boys and Girls in g

| **Centiles** | **GA** | **Boys** | **Girls** | **SE** | **p-value** |
| --- | --- | --- | --- | --- | --- |
| **C5** | 20 | 298,0 | 289,7 | 1,201 | <0,001 |
|  | 25 | 670,8 | 658,3 | 10,869 | 0,21 |
|  | 30 | 1347,7 | 1314,3 | 5,163 | <0,001 |
|  | 35 | 2108,1 | 2059,9 | 30,165 | 0,11 |
|  | 40 | 2776,4 | 2780,4 | 140,429 | 0,40 |
| **C10** | 20 | 308,1 | 299,5 | 1,201 | <0,001 |
|  | 25 | 697,6 | 683,6 | 10,869 | 0,17 |
|  | 30 | 1400,3 | 1365,8 | 5,163 | <0,001 |
|  | 35 | 2208,8 | 2156,3 | 30,165 | 0,09 |
|  | 40 | 2946,3 | 2943,0 | 140,429 | 0,40 |
| **C50** | 20 | 346,4 | 336,7 | 1,201 | <0,001 |
|  | 25 | 798,6 | 776,9 | 10,869 | 0,06 |
|  | 30 | 1593,4 | 1550,2 | 5,163 | <0,001 |
|  | 35 | 2577,1 | 2499,6 | 30,165 | 0,01 |
|  | 40 | 3569,6 | 3531,0 | 140,429 | 0,38 |
| **C90** | 20 | 391,3 | 379,8 | 1,201 | <0,001 |
|  | 25 | 916,5 | 884,1 | 10,869 | 0,005 |
|  | 30 | 1814,1 | 1761,1 | 5,163 | <0,001 |
|  | 35 | 3007,5 | 2911,8 | 30,165 | 0,003 |
|  | 40 | 4325,5 | 4300,3 | 140,429 | 0,39 |
| **C95** | 20 | 405,9 | 393,6 | 1,201 | <0,001 |
|  | 25 | 954,8 | 918,8 | 10,869 | 0,002 |
|  | 30 | 1885,8 | 1831,4 | 5,163 | <0,001 |
|  | 35 | 3151,6 | 3058,9 | 30,165 | 0,004 |
|  | 40 | 4590,7 | 4604,5 | 140,429 | 0,40 |

e-Table 17. Centiles = C (C5 =P5; C10=P10; C50=P50; C90=P90; C95=P95). GA = Gestational Age in weeks. SE = Standard Error
