# Supplementary material for: Sex differences in fetal growth and immediate birth outcomes in a low-risk Caucasian population
Source: Biol Sex Differ. 2019 Sep 9;10:48. doi: 10.1186/s13293-019-0261-7 (PMC6734449; doi:10.1186/s13293-019-0261-7)
Supplement: Supplementary file 6 — Table S2. Fetal charts characteristics, quality control. (DOCX 17 kb) [file 13293_2019_261_MOESM6_ESM.docx]

Fetal charts characteristics.

| **Study** | **Year** | **Country** | **N° women**^ǂ^ | **N° scans** | **Weeks** | **Measurement** | **Design** | **Data Collection** | **Research?** | **Quality score (%)*** |
| --- | --- | --- | --- | --- | --- | --- | --- | --- | --- | --- |
| **Chitty et al^13-15^** | 1994 | UK | 594-649 | 425-649 | 12-42 | BPD,HC,AC,FL | C1 | Prospective | Yes | 78 |
| **Johnsen et al^20^** | 2006 | NO | 650 | 2489-2589 | 10-42 | BPD,HC,AC,FL | LL | Prospective | Yes | 67 |
| **Kurmanavicius et al^16,17^** | 1999 | CH | 6.557 | 5462-6217 | 12-42 | BPD,HC,AC,FL | C1 | Retrospective | No | 65 |
| **Leung et al^19^** | 2008 | CN | 709 | 679-708 | 12-40 | BPD,HC,AC,FL | C1 | Prospective | Yes | 70 |
| **Paladini et al^18^** | 2005 | IT | 626 | 623-625 | 16-40 | BPD,HC,AC,FL | C1 | Prospective | Yes | 65 |
| **Snijders & Nicolaides^12^** | 1994 | UK | 1.040 | 1040 | 14-40 | BPD,HC,AC,FL | C1 | Retrospective | No | 61 |
| **Verburg et al^21^** | 2008 | NL | 3.760 | 20.277-22.271 | 10-40 | BPD,HC,AC,FL | LL | Prospective | Yes | 75 |
| ***Papageorghiou et al^42^*** | *2014* | *INT* | *4.321* | *17.261* | *14-40* | *BPD,HC,AC,FL* | *LL* | *Prospective* | *Yes* | *79** |
| ***Galjaard et al*** | *2019* | *BE* | *9.413* | *27.680* | *12-40* | *BPD,HC,AC,FL* | *LL* | *Retrospective* | *No* | *75** |

Table S2. Characteristics of the highest ranked fetal growth studies (*calculation according to scoring system by Ioannou C et al. BJOG 2012; 119:1425-1439)^37^. ^ǂ^= actual number of women analyzed after exclusion. C1 = cross-sectional -each fetus measured ones-. L = Longitudinal data -each fetus measured longitudinal-. UK = United Kingdom; NO = Norwegian; CH = Suisse; CN = China; IT = Italy; NL = the Netherlands; INT = International; BE = Belgium.

References

12. Snijders RJM and Nicolaides KH. Fetal biometry at 14-40 weeks’ gestation. Ultrasound Obstet Gynecol 1994; 4:34-48.

13. Chitty LS, Altman DG, Henderson A, et al. Charts of fetal size: 2. Head measurements. Br J Obstet Gynaecol 1994; 101:35-43.

14. Chitty LS, Altman DG, Henderson A, et al. Charts of fetal size: 3. Abdominal measurements. Br J Obstet Gynaecol 1994; 101:125-31.

15. Chitty LS, Altman DG, Henderson A, et al. Charts of fetal size: 4. Femur length. Br J Obstet Gynaecol 1994; 101:132-5.

16. Kurmanavicius J, Wright EM, Royston P, et al. Fetal ultrasound biometry: 1. Head reference values. Br J Obstet Gynaecol 1999; 106:126-35.

17. Kurmanavicius J, Wright EM, Royston P, et al. Fetal ultrasound biometry: 2. Abdomen and femur length reference values. Br J Obstet Gynaecol 1999; 106:136-43.

18. Paladini D, Rustico M, Viora E, et al. Fetal size for the Italian population. Normative curves of head, abdomen and long bones. Prenat Diagn 2005; 25:456-64.

19. Leung TN, Pang MW, Daljit SS, et al. Fetal biometry in ethnic Chinese: biparietal diameter, head circumference, abdominal circumference and femur length. Ultrasound Obstet Gynecol 2008; 31:321-7.

20. Johnsen SL, Wilsgaard T, Rasmussen S, et al. Longitudinal reference ranges for estimated fetal weight. Acta Obstet Gynecol Scand 2006; 85:286-97.

21. Verburg BO, Steegers EA, De Ridder M, et al. New charts for ultrasound dating of pregnancy and assessment of fetal growth: longitudinal data from a population-based cohort study. Ultrasound Obstet Gynecol 2008; 31:388-96.

37. Ioannou C, Talbot K, Ohuma E, et al. Systematic review of methodology used in ultrasound studies aimed at creating charts of fetal size. BJOG 2012; 119:1425-1439.

42. Papageorghiou AT, Ohuma EO, Altman DG et al. International standards for fetal growth based on serial ultrasound measurements: the Fetal Growth Longitudinal Study of the INTERGROWTH-21st Project. Lancet 2014; 384:869-79
